# Dietary Supplementation of Inulin Ameliorates Subclinical Mastitis via Regulation of Rumen Microbial Community and Metabolites in Dairy Cows

**DOI:** 10.1128/Spectrum.00105-21

**Published:** 2021-09-08

**Authors:** Yue Wang, Xuemei Nan, Yiguang Zhao, Linshu Jiang, Hui Wang, Fan Zhang, Dengke Hua, Jun Liu, Junhu Yao, Liang Yang, Qingyao Luo, Benhai Xiong

**Affiliations:** a State Key Laboratory of Animal Nutrition, Institute of Animal Science, Chinese Academy of Agricultural Sciences, Beijing, China; b College of Animal Science and Technology, Northwest A&F University, Yangling, China; c Beijing Key Laboratory for Dairy Cow Nutrition, Beijing University of Agriculture, Beijing, China; d Langfang Academy of Agriculture and Forestry, Langfang, China; University of Nevada Reno

**Keywords:** inulin, subclinical mastitis, ruminal microorganisms, rumen metabolites, dairy cow

## Abstract

Subclinical mastitis (SCM) is one of the highly infectious diseases in dairy cows with the characteristics of high incidence and nonvisible clinical symptoms. The gastrointestinal microbiota is closely related to mastitis. Inulin is a prebiotic fiber with functions in improving intestinal microbial communities and enhancing the host’s immunity. However, the impact of dietary inulin on the rumen inner environment remains unknown. The current study investigated whether inulin could relieve SCM by affecting the profiles of ruminal bacterial and metabolites in dairy cows. Inulin inclusion rates were 0, 100, 200, 300, and 400 g/day per cow, respectively. Inulin increased milk yield, milk protein, and lactose and reduced the somatic cell counts (SCC) in milk. In serum, the concentration of proinflammatory cytokines, such as interleukin-6 (IL-6), IL-8, tumor necrosis factor α (TNF-α), and malondialdehyde (MDA) were decreased, and IL-4 and superoxide dismutase (SOD) were increased. Meanwhile, inulin increased the concentration of propionate, butyrate, and lactic acid (LA), while it decreased NH_3_-N in rumen. The propionate- and butyrate-producing bacteria (e.g., *Prevotella* and *Butyrivibrio*) and several beneficial commensal bacteria (e.g., *Muribaculaceae* and *Bifidobacterium*) as well as metabolites related to energy and amino acid metabolism (e.g., melibiose and l-glutamate) were increased. However, several proinflammatory bacteria (e.g., *Clostridia UCG-014*, Streptococcus, and Escherichia*-Shigella*) were decreased, accompanied by the downregulation of lipid proinflammatory metabolites, for example, ceramide(d18:0/15:0) [Cer(d18:0/15:0)] and 17-phenyl-18,19,20-trinor-prostaglandin E_2_. In the current study, the above indicators showed the best response in the 300 g/day inulin group. Overall, dietary supplementation of inulin could alleviate inflammatory responses in cows with SCM through improving the rumen inner environment.

**IMPORTANCE** The correlation between mastitis and the gastrointestinal microbiome in dairy cows has been demonstrated. Regulating the profile of rumen microorganisms may contribute to remission of subclinical mastitis (SCM). Supplementation of inulin in the diets of cows with SCM could increase the abundance of short-chain fatty acid (SCFA)-producing bacteria and beneficial commensal bacteria in rumen and meanwhile the levels of amino acids and energy metabolism. Conversely, the abundance of ruminal bacteria and metabolites with proinflammatory effects were decreased. Our study suggests that the improvement of the rumen internal environment by inulin supplementation could ameliorate inflammatory responses during SCM in dairy cows and thus improve lactation performance and milk quality. Our results provide a theoretical basis for regulation measures of SCM in dairy cows.

## INTRODUCTION

Bovine mastitis (BM) is one of the principal diseases resulting in severe economic losses in the global dairy industry (1). In practical dairy cow production, due to high concealment and a long latency period, the incidence of subclinical mastitis (SCM) is much higher than that of clinical mastitis (CM), accounting for approximately 90 to 95% of BM (2). In addition, dairy cows with SCM in the herd are very likely to become the source of infection for other healthy individuals, resulting in the occurrence of secondary infections in the herd (2). Although milk and udders of cows with SCM do not have visible changes in appearance, damage in lactation performance, immune function, and normal metabolic activities occur (3). Therefore, implementing necessary prevention and control measures in the SCM stage is the key to preventing further aggravation of inflammation.

Recent studies suggested that the gastrointestinal microbiota could affect the occurrence of systemic inflammatory diseases (4–6). Intestinal immune responses induced by commensal populations could regulate the composition of the microbiota (5, 6). Conversely, destruction of the normal microbial community increases the risk of pathogen infection, overgrowth of harmful pathogens, and inflammatory diseases (4). Ma et al. (7) transplanted the feces of cows with mastitis into the intestines of sterile mice, which induced symptoms of mastitis in mice. Our previous studies found that, compared with healthy cows, ruminal bacteria and metabolites related to inflammation were significantly increased while the commensal population, including short-chain fatty acid (SCFA)-producing bacteria, was reduced in cows with SCM and CM (8). In addition, the decrease in SCFAs in intestines caused by intestinal dysbacteriosis could further increase permeability of the blood-milk barrier, which thereby aggravates mastitis (9). The impact of intestinal microbes on mastitis is based on the endogenous entero-mammary pathway of microbes (10). However, unlike in monogastric animals, rumen microbes are the largest microbial community in dairy cows, and the rumen is the main organ where SCFAs are produced (11). Therefore, we attempted to modulate the rumen inner environment (the profile of microbes and metabolites) through nutritional regulation to relieve SCM in dairy cows.

Inulin is a kind of dietary fiber and is abundantly present in the family *Asteraceae*, among which Jerusalem artichoke tubers are one of the main sources of inulin (about 60 to 75% of dry matter [DM]) (12). Inulin is composed of β-d-fructopyranose connected by β-(1-2)-glycosidic bonds, which can be hydrolyzed into d-fructose by inulase. However, inulase in both humans and animals is generally absent (13). Inulin has been reported as a prebiotic with functions of promoting the proliferation of *Bifidobacterium* and *Lactobacilli*, reducing intestinal pathogens through competitive inhibition, and further promoting the host immune and defense system (12, 13). The mechanism may involve changes in microbial metabolic pathways and production of beneficial microbial metabolites, especially SCFAs.

The profile of the rumen microbial community and the abundance of metabolites, including SCFAs, play key roles in the health status and production performance of dairy cows (11, 14). Hence, the objective of our study is to investigate the effects of inulin on the profile of ruminal microbiota and their metabolites, lactation performance, and inflammatory response in SCM cows and to further explore whether dietary inulin supplementation could alleviate the inflammatory symptoms of SCM.

## RESULTS

### Effect of inulin supplementation on production performance, milk yield, and compositions.

Production and lactation performance measures of SCM cows are listed in Table 1. Compared with the control group, milk yield (*P = *0.031), energy-corrected milk (ECM) (*P = *0.040), fat-corrected milk (FCM) (*P = *0.042), milk protein (*P = *0.034), and milk lactose (*P = *0.017) were significantly increased in the I-3 group, except that ECM and FCM had no significant difference between the I-2 and I-3 groups. However, milk electrical conductivity (EC) (*P = *0.083) presented a downward trend with the addition of inulin, which was lowest in the I-3 group. Meanwhile, milk urea nitrogen (MUN) (*P = *0.046), blood urea nitrogen (BUN) (*P = *0.035), and milk somatic cell count (SCC) (*P < *0.01) values were significantly decreased and lowest in the I-3 group.

**TABLE 1:** Effects of inulin addition on production and lactation performance in dairy cows with SCM

Item^*a*^	Group (*n* = 6)^*a*^					SEM^*a*^	*P* value
	Con	I-1	I-2	I-3	I-4		
DMI, kg/day	23.1	22.3	23.9	24.5	23.2	0.15	0.142
Milk yield, kg/day	31.4^c^	31.7^c^	33.6^b^	34.2^a^	32.6^b^	0.35	0.031
ECM, kg/day^*b*^	29.9^c^	30.0^c^	32.0^a^	32.7^a^	31.2^bc^	0.45	0.040
FCM, kg/day^*c*^	30.5^c^	30.6^c^	32.3^a^	32.8^a^	31.6^bc^	0.48	0.042
Milk fat, %	3.81	3.76	3.74	3.72	3.80	0.047	0.175
Milk protein, %	3.07^c^	3.11^c^	3.22^ab^	3.29^a^	3.18^b^	0.018	0.034
F/P	1.24	1.21	1.16	1.13	1.19	0.019	0.183
Milk lactose, %	4.01^c^	4.02^c^	4.18^b^	4.20^a^	4.12^b^	0.031	0.017
MUN, mmol/liter	6.02^a^	5.81^ab^	5.89^ab^	5.75^b^	5.78^b^	0.089	0.046
BUN, mmol/liter	4.52^a^	4.48^ab^	4.33^b^	3.98^c^	4.15^bc^	0.069	0.035
SCC, ×10^3^/ml	683^a^	660^a^	527^b^	421^c^	491^bc^	13.8	<0.01
EC, mS/cm	7.85	7.88	7.11	7.07	7.35	0.102	0.083
FCR^*d*^	1.32	1.37	1.35	1.34	1.36	0.009	0.247

a DMI, dry matter intake; ECM, energy-corrected milk; FCM, fat-corrected milk; F/P, fat-to-protein ratio; MUN, milk urea nitrogen; BUN, blood urea nitrogen; SCC, somatic cell counts; EC, electrical conductivity of milk; FCR, feed conversion ratio; SEM, standard error of mean; Con, control group; I-1, inulin-1 group with an inulin addition level of 100 g/day per cow; I-2, inulin-2 group with an inulin addition level of 200 g/day per cow; I-3, inulin-3 group with an inulin addition level of 300 g/day per cow; I-4, inulin-4 group with an inulin addition level of 400 g/day per cow; ^a, b, c^, within a row, different letters differed significantly (*P *< 0.05).

b ECM (kg/day) = milk yield (kg/day) × (383 × fat [%] + 242 × protein [%] + 783.2)/3,140 (60).

c FCM (kg/day) = 0.4 × milk yield (kg/day) + 15 × milk yield (kg/day) × fat (%) (60).

d FCR (%) = FCM (kg/day)/DMI (kg/day) (60).

### Inflammatory response and lipopolysaccharide concentration.

**FIG 1:**
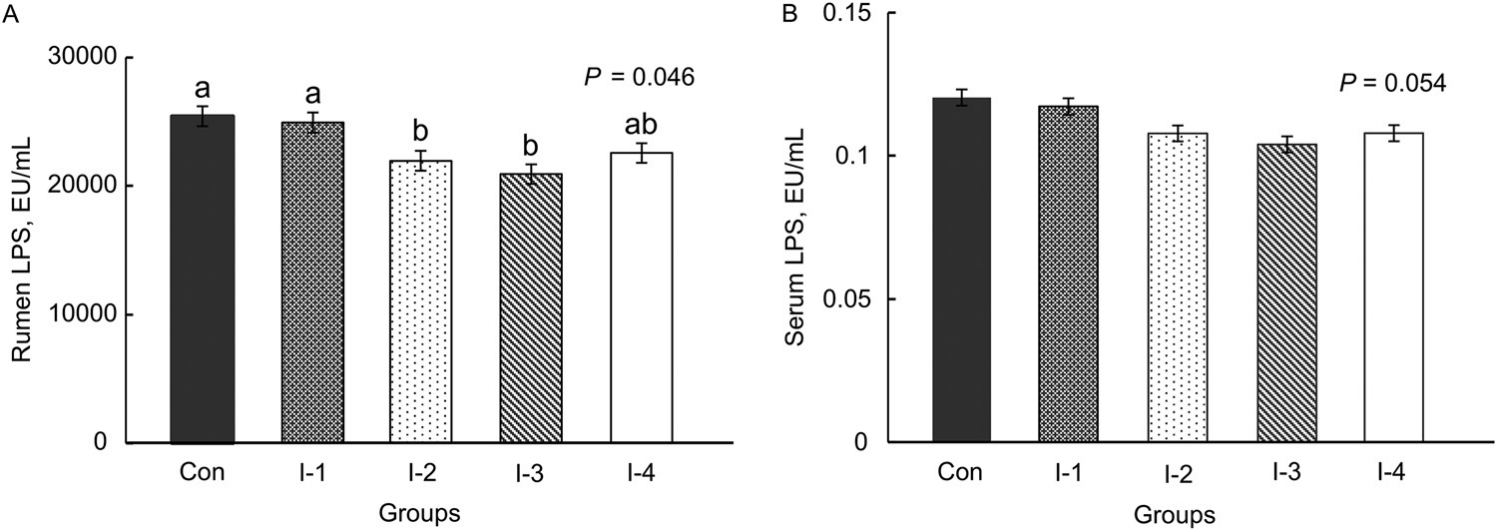
Concentrations of lipopolysaccharide (LPS) in rumen fluid (A) and serum (B) in control animals and the different inulin treatment groups. Con, control group; I-1, inulin-1 group with an inulin addition level of 100 g/day per cow; I-2, inulin-2 group with an inulin addition level of 200 g/day per cow; I-3, inulin-3 group with an inulin addition level of 300 g/day per cow; I-4, inulin-4 group with an inulin addition level of 400 g/day per cow; EU/ml, endotoxin units per ml; ^a, b, ab^, different letters differed significantly (*P *< 0.05).

As shown in Table 2, inulin supplementation elevated the concentrations of interleukin-4 (IL-4) (*P = *0.042) and superoxide dismutase (SOD) (*P = *0.041) and decreased the levels of IgG (*P = *0.013), IL-6 (*P = *0.035), IL-8 (*P = *0.034), tumor necrosis factor-α (TNF-α) (*P = *0.042), prostaglandin E_2_ (PGE_2_) (*P = *0.036), and malondialdehyde (MDA) (*P = *0.022) in serum. Among them, the highest value of SOD, as well as the lowest values of IgG, TNF-α, and MDA, appeared in the I-2 and I-3 groups. The lowest value of PGE_2_ was found in the I-3 and I-4 groups. Meanwhile, the greatest concentration of IL-4 and least concentrations of IL-6 and IL-8 were found in the I-3 group. Compared with the control group, the lipopolysaccharide (LPS) concentration in rumen (*P = *0.046) was reduced and the concentration in serum (*P = *0.054) demonstrated a downtrend (*P = *0.054) in the I-3 group (Fig. 1).

**TABLE 2:** Effects of inulin addition on the inflammatory cytokine and oxidative stress indexes in serum of dairy cows with SCM

Item^*a*^	Group (*n* = 6)^*a*^					SEM^*a*^	*P* value
	Con	I-1	I-2	I-3	I-4		
TP, g/liter	62.1	63.4	63.7	63.8	62.6	0.15	0.152
ALB, g/liter	40.2	42.8	42.6	42.7	41.0	0.33	0.274
GLB, g/liter	21.9	20.6	21.1	21.1	21.6	0.31	0.692
IgA, μg/ml	11.7	10.6	13.8	11.3	10.2	0.46	0.284
IgG, mg/ml	6.04^a^	5.73^ab^	4.99^b^	4.87^b^	5.72^ab^	0.053	0.013
IgM, mg/ml	72.2	72.7	70.3	71.1	69.6	0.81	0.220
IL-6, ng/liter	425^a^	325^b^	300^bc^	294^c^	341^b^	7.7	0.035
IL-8, ng/liter	322^a^	325^a^	294^bc^	277^c^	302^bc^	4.9	0.034
IL-10, pg/ml	15.1	14.2	17.6	16.7	16.1	0.40	0.075
IL-4, pg/ml	34.8^c^	35.7^c^	39.5^b^	42.3^a^	38.2^b^	0.93	0.042
IL-2, pg/ml	56.7	54.5	51.3	52.7	53.2	0.68	0.067
TNF-α, ng/liter	217^a^	206^b^	182^c^	180^c^	205^b^	4.4	0.042
PGE_2_, pg/ml	94.4^a^	85.1^b^	84.6^b^	80.1^c^	81.2^c^	1.43	0.036
GSH-Px, μmol/liter	4.33	4.28	4.30	4.56	4.48	0.083	0.174
SOD, U/ml	45.8^c^	50.9^b^	52.7^a^	53.2^a^	50.2^b^	0.35	0.041
MDA, nmol/ml	2.57^a^	2.51^a^	1.87^c^	1.85^c^	2.01^b^	0.053	0.022

a TP, total protein; ALB, albumin; GLB, globulin; IL, interleukin; TNF-α, tumor necrosis factor-α; PGE_2_, prostaglandin E_2_; GSH-Px, glutathione peroxidase; MDA, malondialdehyde; SOD, superoxide dismutase; SEM, standard error of the mean; Con, control group; I-1, inulin-1 group with an inulin addition level of 100 g/day per cow; I-2, inulin-2 group with an inulin addition level of 200 g/day per cow; I-3, inulin-3 group with an inulin addition level of 300 g/day per cow; I-4, inulin-4 group with an inulin addition level of 400 g/day per cow; ^a, b, c^, within a row, different letters differed significantly (*P *< 0.05).

### Effects of inulin addition on rumen fermentation parameters.

As shown in Table 3, the concentrations of propionate (*P = *0.021), butyrate (*P = *0.034), total volatile fatty acids (TVFA) (*P = *0.041), and lactic acid (LA) (*P = *0.037) in the rumen of SCM cows were increased after inulin addition and were highest in the I-3 group (the concentration of LA demonstrated no significant difference between the I-2 and I-3 groups). The concentration of isobutyrate (*P = *0.084) had a tendency to increase, while the concentration of NH_3_-N (*P < *0.01) and the ratio of acetate to propionate (A/P) (*P = *0.041) were reduced and lowest in the I-3 group (A/P demonstrated no significant difference between the I-2 and I-3 groups). Additionally, the pH value in rumen (*P = *0.083) and the concentration of rumen urea nitrogen (RUN) (*P = *0.064) both demonstrated a decreasing trend with inulin supplementation.

**TABLE 3:** Effects of inulin addition on rumen fermentation in dairy cows with SCM

Item^*a*^	Group (*n* = 6)^*a*^					SEM^*a*^	*P* value
	Con	I-1	I-2	I-3	I-4		
pH	6.76	6.77	6.64	6.69	6.67	0.019	0.083
NH_3_-N, mg/dl	10.5^a^	9.41^b^	9.13^b^	8.93^bc^	9.13^b^	0.096	<0.01
RUN, mmol/liter	2.73	2.62	2.40	2.42	2.50	0.073	0.055
LA, mmol/liter	1.33^c^	1.37^c^	1.54^a^	1.57^a^	1.48^b^	0.023	0.037
Acetate, mmol/liter	71.4	70.8	72.6	73.1	72.3	0.35	0.115
Propionate, mmol/liter	20.1^c^	20.9^c^	22.8^b^	23.2^a^	22.3^b^	0.19	0.021
A/P	3.55^a^	3.39^b^	3.18^c^	3.15^c^	3.24^b^	0.029	0.041
Butyrate, mmol/liter	10.4^c^	12.6^c^	13.5^b^	14.2^a^	13.1^b^	0.20	0.034
Isobutyrate, mmol/liter	0.68	0.71	0.75	0.73	0.74	0.012	0.084
Valerate, mmol/liter	1.39	1.34	1.43	1.46	1.46	0.023	0.311
Isovalerate, mmol/liter	1.38	1.31	1.39	1.44	1.4	0.022	0.164
TVFA, mmol/liter	105^c^	108^c^	112^b^	114^a^	111^b^	0.54	0.041

a RUN, rumen urea nitrogen; LA, lactic acid; A/P, the ratio of acetate to propionate; TVFA, total volatile fatty acids; SEM, standard error of the mean; Con, control group; I-1, inulin-1 group with an inulin addition level of 100 g/day per cow; I-2, inulin-2 group with an inulin addition level of 200 g/day per cow; I-3, inulin-3 group with an inulin addition level of 300 g/day per cow; I-4, inulin-4 group with an inulin addition level of 400 g/day per cow; ^a, b, c^, within a row, different letters differed significantly (*P *< 0.05).

### Effect of inulin on diversity, richness, taxonomic annotation, and composition of the ruminal microflora community.

**FIG 2:**
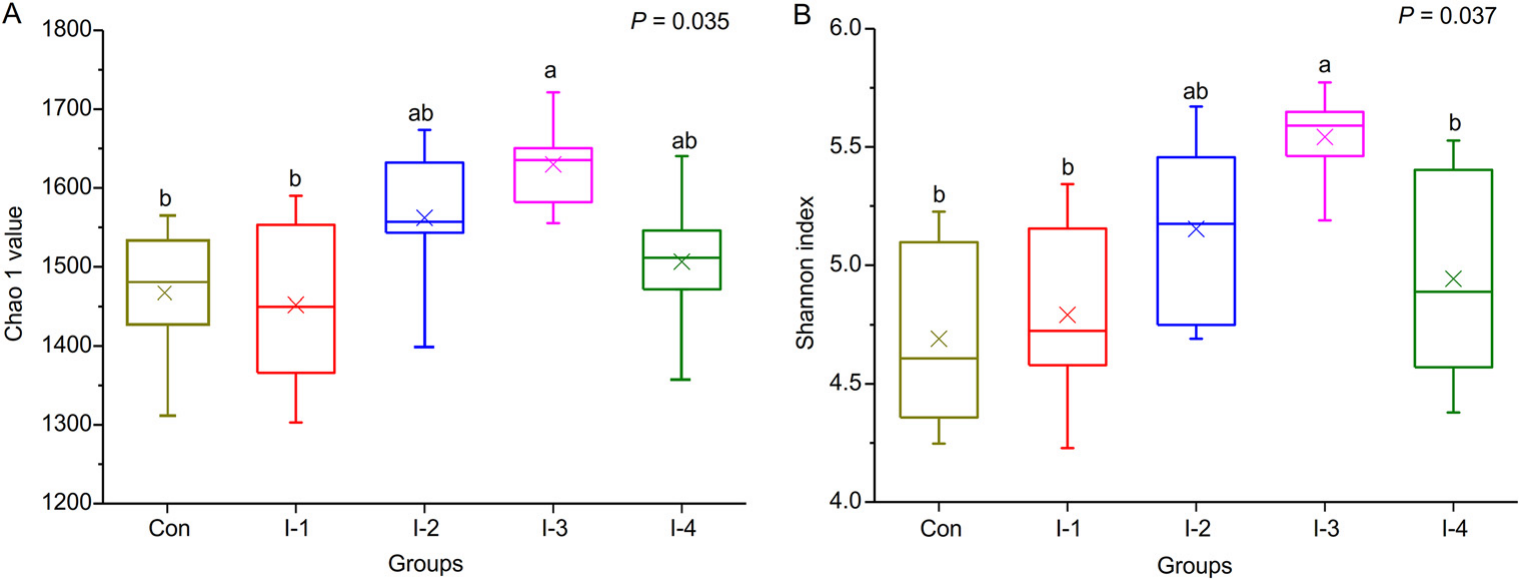
Effects of inulin supplementation on rumen microbial richness (A) and diversity (B) in dairy cows with SCM. The richness of ruminal bacteria is evaluated by the Chao 1 value, and the diversity of ruminal bacteria is estimated by the Shannon index. Con, control group; I-1, inulin-1 group with an inulin addition level of 100 g/day per cow; I-2, inulin-2 group with an inulin addition level of 200 g/day per cow; I-3, inulin-3 group with an inulin addition level of 300 g/day per cow; I-4, inulin-4 group with an inulin addition level of 400 g/day per cow; ^a, b, ab^, different letters differed significantly (*P *< 0.05).

A total of 1,303,222 high-quality 16S rRNA gene sequences were obtained from 30 rumen fluid samples with Good’s coverage of 99% in all samples. Meanwhile, the rarefaction curves revealed that the increment of the number of species and diversity index gradually tended to be flat with an increase in the number of reads sampled (Fig. S1 in the supplemental material), which implied that the amount of sequencing data was sufficient to reflect most of the microbial diversity information in the samples. As shown in Table S2 and Fig. 2, compared with the control group, the Sobs (*P = *0.041) and Chao (*P = *0.035) indexes, which reflect the microbial richness, as well as Shannon (*P = *0.037) and Simpson (*P = *0.021) indexes, which indicate bacterial diversity, were significantly increased in SCM cows fed inulin and peaked in the I-3 group.

**FIG 3:**
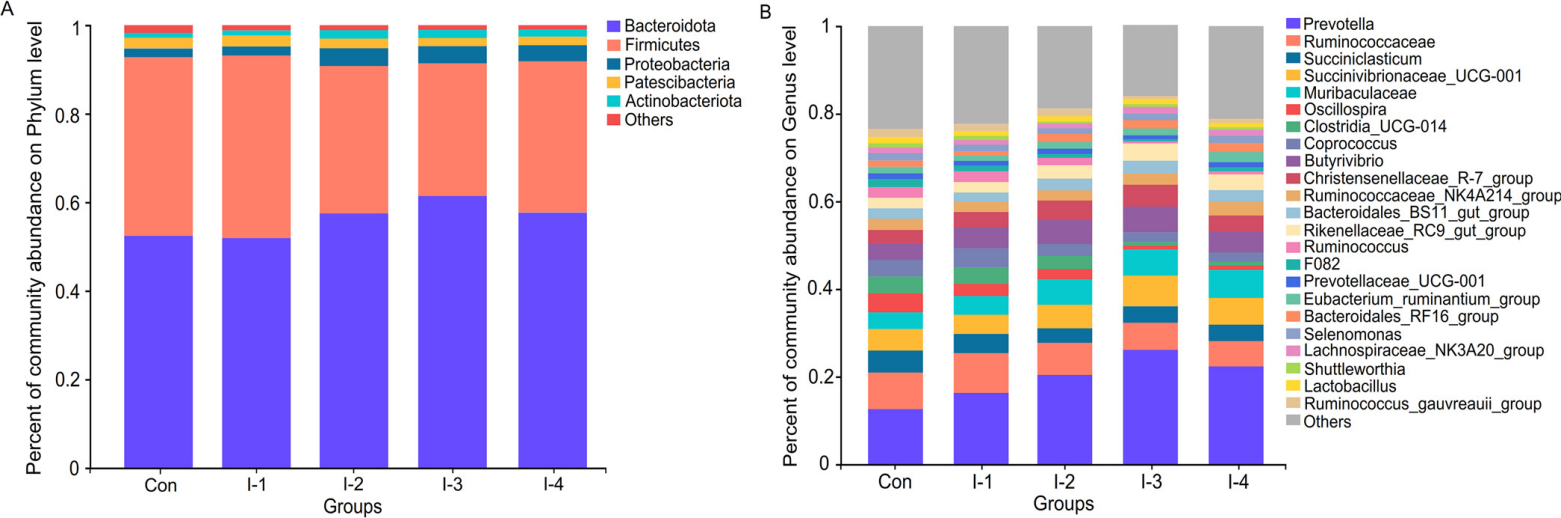
Composition of ruminal bacteria among control and different inulin addition groups at the phylum (A) and genus (B) levels. Con, control group; I-1, inulin-1 group with an inulin addition level of 100 g/day per cow; I-2, inulin-2 group with an inulin addition level of 200 g/day per cow; I-3, inulin-3 group with an inulin addition level of 300 g/day per cow; I-4, inulin-4 group with an inulin addition level of 400 g/day per cow.

After sequence clustering and quality filtering, a total of 2,151 operational taxonomic units (OTUs) with >97% similarity were identified. Using the RDP classifier Bayesian algorithm to perform taxonomic analysis on 97% similar OTU representative sequences, 18 phyla and 266 genera of bacteria were obtained (Table S3 and S4). At the phylum level, *Bacteroidota* (51.5 ± 1.65%), *Firmicutes* (36.8 ± 1.47%), *Proteobacteria* (6.85 ± 0.94%), *Patescibacteria* (2.16 ± 0.10%), and *Actinobacteriota* (1.49 ± 0.07%) were predominant across the 5 groups (Fig. 3A). At the genus level, *Prevotella* (18.6 ± 1.30%), *Ruminococcaceae* (7.29 ± 0.21%), *Succinivibrionaceae*_UCG-001 (4.80 ± 0.27%), *Succiniclasticum* (3.99 ± 0.34%), *Muribaculaceae* (3.93 ± 0.13%), *Oscillospira* (2.29 ± 0.07%), etc., were the most abundant (Fig. 3B).

**FIG 4:**
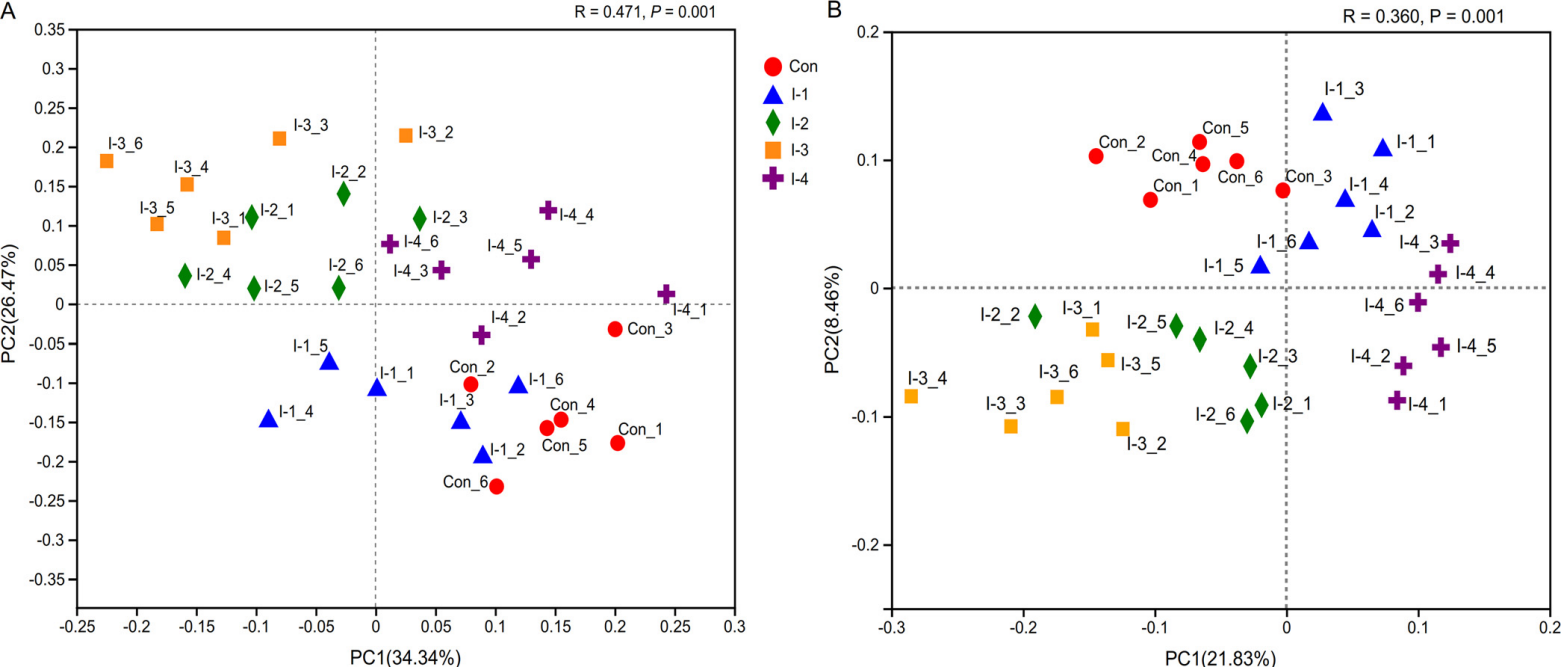
Principal coordinate analysis (PCoA) of rumen microbial communities among control and different inulin addition groups based on weighted normalized UniFrac (A) and unweighted UniFrac distance algorithm (B). Con, control group; I-1, inulin-1 group with an inulin addition level of 100 g/day per cow; I-2, inulin-2 group with an inulin addition level of 200 g/day per cow; I-3, inulin-3 group with an inulin addition level of 300 g/day per cow; I-4, inulin-4 group with an inulin addition level of 400 g/day per cow.

Weighted normalized UniFrac and unweighted UniFrac distance algorithms were used to perform the principal coordinates analysis (PCoA) of rumen microbial communities (Fig. 4A and B). Difference tests among groups were analyzed via the analysis of similarities (ANOSIM) method, with *R* acting as a statistic. In Fig. 4, an *R* of >0 indicated that the intragroup distance was less than the intergroup distance, which meant the grouping was valid. Furthermore, the difference of microbial composition among control and inulin treatment groups was significant (*P = *0.001). Hierarchical clustering analysis of the top 50 bacteria in relative abundance was conducted to further investigate the effects of inulin on ruminal microbial profiles (Fig. S2). In the present study, the distribution of ruminal bacteria was divided into two major categories. Similar to the results of the PCoA, the profiles of ruminal bacteria in the control and I-1 groups could not be clearly distinguished, with Streptococcus, *Ruminococcus*, *Neisseriaceae*, *Coprococcus*, *Ruminococcaceae*, Escherichia*-Shigella*, Staphylococcus, etc., being the most abundant. However, with the increase of inulin addition levels, the ruminal bacterial profile was significantly changed. Compared with the control group, *Prevotella*, *Muribaculaceae*, *Christensenellaceae* R-7 group, *Butyrivibrio*, *Bifidobacterium*, *Lactobacillus*, etc., were the predominant bacteria genera in the I-3 and I-2 groups. Meanwhile, the relative abundances of Treponema, *Candidatus Saccharimonas*, *Succiniclasticum*, *Prevotellaceae*_UCG-001, *Lachnospiraceae* NK4A136 group, and Eubacterium hallii group in the I-4 group were higher than in the control group.

### Differentially abundant ruminal bacteria among control and different inulin addition groups.

The differentially abundant bacterial communities among control and inulin treatment groups are presented in Table 4. At the phylum level, *Bacteroidota* (false-discovery rate [FDR]-adjusted *P *= 0.033), *Proteobacteria* (FDR-adjusted *P *= 0.048), and *Actinobacteriota* (FDR-adjusted *P *= 0.037) were increased in the inulin supplementation groups compared with the control group and were mostly abundant in the I-3 group (except for *Proteobacteria*, which demonstrated no significant difference between the I-2 and I-3 groups). The relative abundance of *Firmicutes* (FDR-adjusted *P *= 0.037) was decreased with the addition of inulin and was lowest in the I-3 group.

**TABLE 4:** Significantly different ruminal bacteria composition at phylum and genus levels in cows with SCM fed different inulin dosages

Bacterium	Group (*n* = 6)^*a*^					SEM^*a*^	*P* value	FDR^*a*^
	Con	I-1	I-2	I-3	I-4			
*Bacteroidota*	46.6^c^	46.7^c^	52.2^b^	56.4^a^	52.6^b^	1.65	0.004	0.033
*Prevotella*	14.2^c^	16.7^c^	19.3^b^	22.4^a^	20.4^b^	1.299	<0.001	0.013
*Muribaculaceae*	3.55^c^	3.67^c^	3.80^c^	5.04^a^	4.20^b^	0.126	0.005	0.033
*Bacteroidales* BS11 gut group	2.33^b^	2.40^b^	3.81^a^	3.46^a^	3.65^a^	0.234	0.011	0.042
*Rikenellaceae* RC9 gut group	2.22^c^	2.47^c^	3.72^b^	4.23^a^	4.04^a^	0.168	0.006	0.039
*Bacteroidales* RF16 group	1.42^b^	1.37^b^	2.22^a^	2.60^a^	2.35^a^	0.112	0.017	0.045
*Firmicutes*	42.5^a^	42.9^a^	36.0^b^	31.3^c^	35.4^b^	1.47	0.009	0.037
*Ruminococcaceae*	8.24^a^	8.52^a^	6.78^b^	6.13^b^	6.60^b^	0.214	0.004	0.035
*Oscillospira*	3.25^a^	2.95^b^	2.61^b^	1.07^c^	1.46^c^	0.067	0.011	0.042
*Clostridia*_UCG-014	2.97^a^	2.69^a^	2.26^a^	1.58^b^	1.65^b^	0.078	0.008	0.035
*Coprococcus*	2.89^a^	2.54^a^	1.54^b^	1.18^c^	1.30^bc^	0.124	0.004	0.036
*Butyrivibrio*	2.70^c^	3.11^b^	4.35^a^	4.71^a^	3.89^b^	0.197	0.002	0.031
*Christensenellaceae* R-7 group	2.69^c^	2.77^c^	3.84^b^	4.29^a^	3.75^b^	0.182	0.004	0.038
Eubacterium ruminantium group	1.47^c^	1.32^c^	2.53^b^	2.46^b^	3.16^a^	0.137	0.002	0.031
*Lachnospiraceae* NK3A20 group	1.33^b^	1.23^b^	1.56^ab^	1.89^a^	1.80^a^	0.106	0.015	0.046
*Lactobacillus*	1.23^c^	1.40^c^	2.36^a^	2.35^a^	1.98^b^	0.111	0.013	0.042
Streptococcus	0.94^a^	0.73^a^	0.43^b^	0.39^b^	0.41^b^	0.022	0.017	0.048
Staphylococcus	0.17^a^	0.12^b^	0.11^b^	0.09^c^	0.07^c^	0.014	0.014	0.045
*Proteobacteria*	6.38^b^	6.23^b^	7.17^a^	7.39^a^	7.09^ab^	0.937	0.018	0.048
*Succinivibrionaceae*_UCG-001	3.89^b^	4.12^b^	4.93^ab^	5.44^a^	5.21^a^	0.270	0.007	0.038
*Neisseriaceae*	0.60^a^	0.48^a^	0.19^b^	0.11^b^	0.12^b^	0.012	0.015	0.046
Escherichia *-Shigella*	0.64^a^	0.53^a^	0.25^b^	0.18^c^	0.21^b^	0.041	0.014	0.045
*Acetobacter*	0.08^b^	0.14^a^	0.14^a^	0.17^a^	0.17^a^	0.005	0.017	0.048
*Actinobacteriota*	1.10^c^	1.06^c^	1.62^b^	1.85^a^	1.54^b^	0.07	0.006	0.037
*Bifidobacterium*	0.96^c^	0.97^c^	1.41^b^	1.65^a^	1.25^b^	0.080	0.013	0.043

a Con, control group; I-1, inulin-1 group with an inulin addition level of 100 g/day per cow; I-2, inulin-2 group with an inulin addition level of 200 g/day per cow; I-3, inulin-3 group with an inulin addition level of 300 g/day per cow; I-4, inulin-4 group with an inulin addition level of 400 g/day per cow; SEM, standard error of the mean; FDR, false-discovery rate; ^a, b, c^, within a row, different letters differed significantly (*P *< 0.05).

Genus-level classification of rumen microbial communities showed higher abundances of *Prevotella* (FDR-adjusted *P *= 0.013), *Muribaculaceae* (FDR-adjusted *P *= 0.033), *Bacteroidales* BS11 gut group (FDR-adjusted *P *= 0.042), *Bacteroidales* RF16 group (FDR-adjusted *P *= 0.045), *Butyrivibrio* (FDR-adjusted *P = *0.031), *Lactobacillus* (FDR-adjusted *P = *0.042), and *Bifidobacterium* (FDR-adjusted *P = *0.043) in inulin groups than in the control group, and most of these bacteria were most abundant in the I-3 group. However, the relative abundances of *Ruminococcaceae* (FDR-adjusted *P = *0.035), *Oscillospira* (FDR-adjusted *P = *0.042), *Clostridia*_UCG-014 (FDR-adjusted *P = *0.035), *Coprococcus* (FDR-adjusted *P = *0.036), Streptococcus (FDR-adjusted *P = *0.048), Staphylococcus (FDR-adjusted *P = *0.045), *Neisseriaceae* (FDR-adjusted *P = *0.046), and Escherichia*-Shigella* (FDR-adjusted *P = *0.045) were reduced with the supplementation of inulin, and the lowest abundances of the above bacteria were also detected in the I-3 group.

### Correlation analysis between differentially abundant bacteria and lactation performance as well as rumen fermentation parameters.

**FIG 5:**
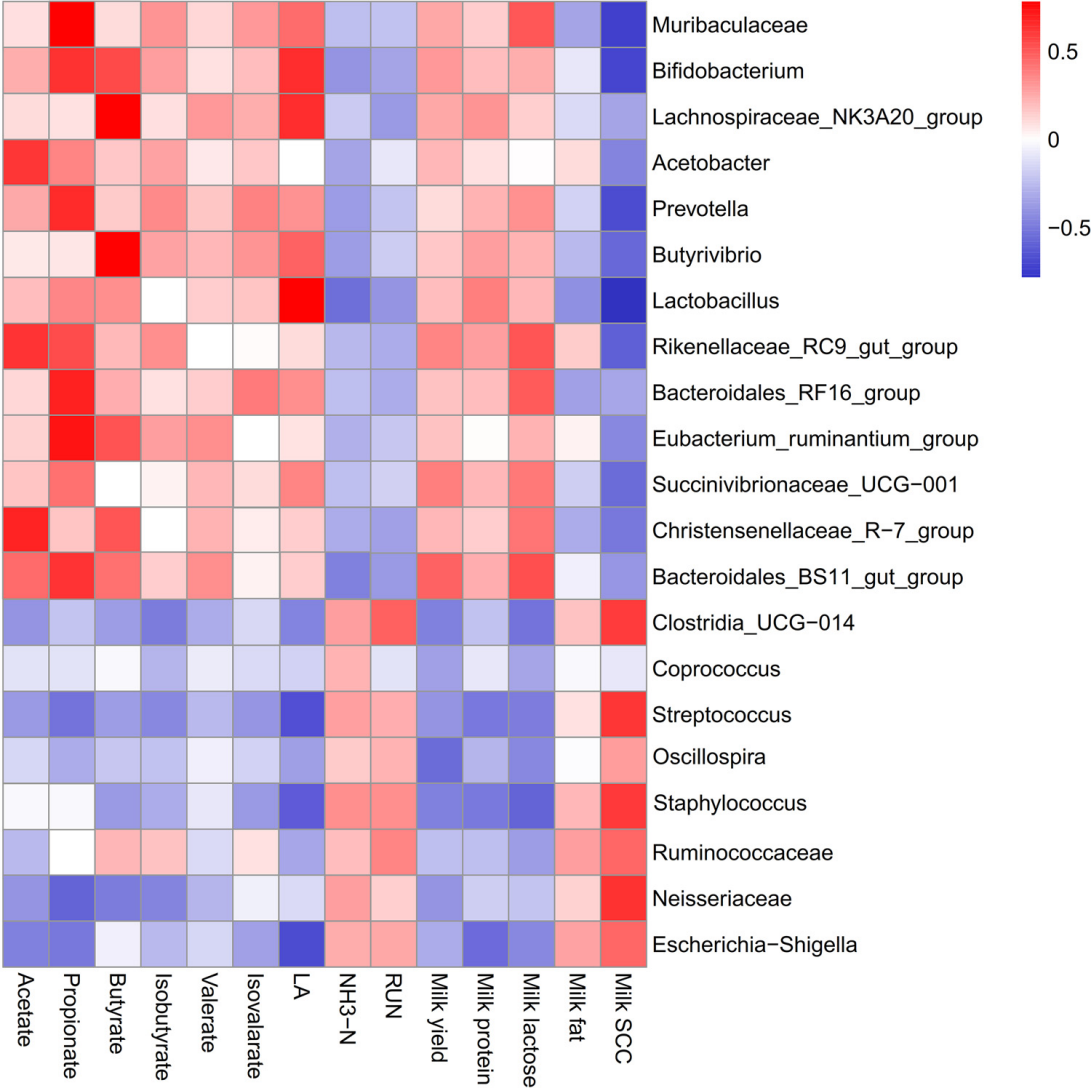
Correlation analysis between significantly differentially abundant ruminal microorganisms, milk compositions, and rumen fermentation parameters among control animals and animals treated with different levels of inulin. Each row in the graph represents a genus, each column represents the milk composition or rumen fermentation parameter, and each lattice represents a Spearman correlation coefficient between a bacterium and a metabolite. Red represents a positive correlation, while blue represents a negative correlation.

The ruminal microbe profile in dairy cows plays a decisive role on the rumen fermentation pattern, milk composition, and health status of the gastrointestinal tract. As shown in Fig. 5 and Table S5, *Acetobacter* (*r* = 0.625, FDR-adjusted *P = *0.028), *Rikenellaceae* RC9 gut group (*r* = 0.430, FDR-adjusted *P = *0.038), and *Christensenellaceae* R-7 group (*r* = 0.547, FDR-adjusted *P = *0.044) were positively correlated with acetate in the rumen. *Muribaculaceae* (*r* = 0.546, FDR-adjusted *P = *0.021), *Bifidobacterium* (*r* = 0.637, FDR-adjusted *P = *0.015), *Prevotella* (*r* = 0.589, FDR-adjusted *P = *0.029), *Rikenellaceae* RC9 gut group (*r* = 0.615, FDR-adjusted *P = *0.030), Eubacterium ruminantium group (*r* = 0.527, FDR-adjusted *P = *0.028), *Bacteroidales* RF16 group (*r* = 0.615, FDR-adjusted *P < *0.01), and *Bacteroidales* BS11 gut group (*r* = 0.551, FDR-adjusted *P < *0.01) were positively correlated with propionate. *Bifidobacterium* (*r* = 0.541, FDR-adjusted *P = *0.040), *Lachnospiraceae* NK3A20 group (*r* = 0.574, FDR-adjusted *P = *0.021), *Butyrivibrio* (*r* = 0.533, FDR-adjusted *P = *0.022), Eubacterium ruminantium group (*r* = 0.425, FDR-adjusted *P = *0.043), and *Christensenellaceae* R-7 group (*r* = 0.475, FDR-adjusted *P* = 0.043) were positively correlated with butyrate. *Bifidobacterium* (*r* = 0.603, FDR-adjusted *P = *0.042), *Lachnospiraceae* NK3A20 group (*r* = 0.553, FDR-adjusted *P = *0.039), and *Lactobacillus* (*r* = 0.577, FDR-adjusted *P < *0.01) were positively correlated with LA. By contrast, Streptococcus (*r* = −0.621, FDR-adjusted *P = *0.025), Staphylococcus (*r* = −0.561, FDR-adjusted *P = *0.033), and Escherichia*-Shigella* (*r* = −0.583, FDR-adjusted *P = *0.032) were negatively correlated with LA.

For milk composition, *Muribaculaceae* (*r* = 0.486, FDR-adjusted *P = *0.043), *Rikenellaceae* RC9 gut group (*r* = 0.549, FDR-adjusted *P = *0.040), *Bacteroidales* RF16 group (*r* = 0.535, FDR-adjusted *P = *0.022), and *Bacteroidales* BS11 gut group (*r* = 0.501, FDR-adjusted *P = *0.036) were positively associated with milk lactose. However, *Clostridia*_UCG-014 (*r* = −0.521, FDR-adjusted *P = *0.041) and Staphylococcus (*r* = −0.514, FDR-adjusted *P = *0.039) were negatively correlated with milk lactose. In the current study, *Muribaculaceae* (*r* = −0.575, FDR-adjusted *P = *0.041), *Butyrivibrio* (*r* = −0.563, FDR-adjusted *P = *0.034), and *Lactobacillus* (*r* = −0.607, FDR-adjusted *P = *0.037) were negatively correlated with milk SCC. However, *Clostridia*_UCG-014 (*r* = 0.497, FDR-adjusted *P = *0.048), Streptococcus (*r* = 0.621, FDR-adjusted *P = *0.025), Staphylococcus (*r* = 0.547, FDR-adjusted *P = *0.022), *Ruminococcaceae* (*r* = 0.462, FDR-adjusted *P = *0.048), *Neisseriaceae* (*r* = 0.530, FDR-adjusted *P = *0.042), and Escherichia*-Shigella* (*r* = 0.463, FDR-adjusted *P = *0.040) were positively associated with milk SCC.

### Metabolic profiling of ruminal fluid samples.

**FIG 6:**
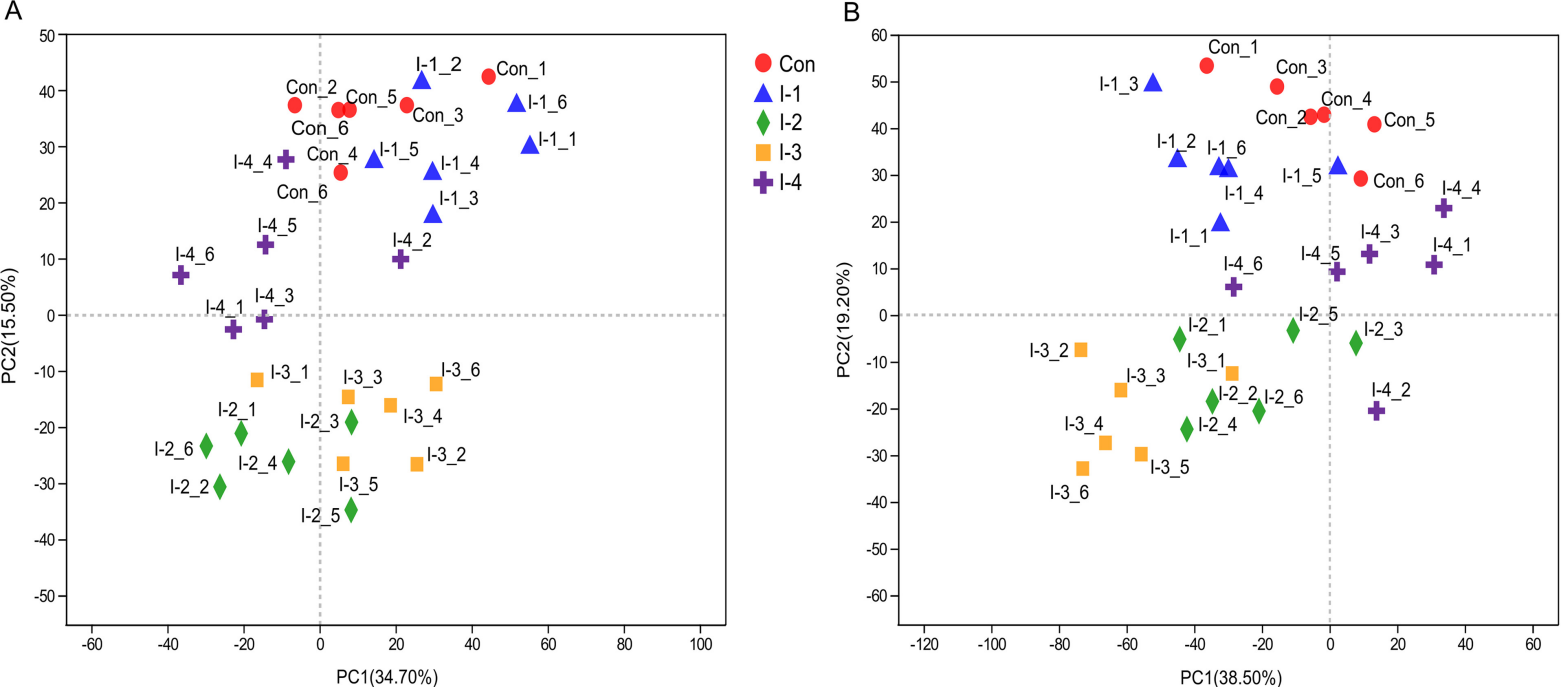
Principal-component analysis (PCA) of the rumen metabolome among control and different inulin addition groups in positive (A) and negative (B) ion mode. Con, control group; I-1, inulin-1 group with an inulin addition level of 100 g/day per cow; I-2, inulin-2 group with an inulin addition level of 200 g/day per cow; I-3, inulin-3 group with an inulin addition level of 300 g/day per cow; I-4, inulin-4 group with an inulin addition level of 400 g/day per cow.

An untargeted metabolomics analysis based on liquid chromatography-mass spectrometry (LC-MS) technology was used to investigate the metabolite profiles in rumen fluid samples. Total ion chromatograms (TICs) of quality control (QC) samples at positive and negative ion modes are shown in Fig. S3A and B, respectively. Through overlapping comparison, the response intensity and retention time of each chromatographic peak was found to be basically overlapped, which reflected the reliability of the experiment data quality. To reflect the overall difference among different groups and the degree of variability among samples within the group, a principal-component analysis (PCA) was conducted based on an unsupervised multivariate statistical analysis method. As shown in Fig. 6A and B, in both positive and negative ion modes, the different inulin treatment groups and control group could be separated except for the I-1 group, which was closer to the control group than other treatment groups.

To further visualize the differences in metabolites among different groups, partial least squares discriminant analysis (PLS-DA) and orthogonal partial least squares discriminant analysis (OPLS-DA) were performed based on a supervised discriminant analysis method to conduct multigroup variance analysis and pairwise comparison at positive and negative ion modes, respectively (Fig. S4, S5, and S6). In PLS-DA and OPLS-DA score plots, the data at positive and negative ionization analysis were separated distinctly among control and inulin treatment groups. Additionally, *R*^2^*X*(cum) and *R*^2^*Y*(cum) represent the cumulative interpretation rates to the *X* and *Y* matrices of the model, respectively; *Q*^2^(cum) represents the predictive ability of the model. The closer these three indicators are to 1, the higher the predicting ability of the model would be. The values of *R*^2^*X*(cum), *R*^2^*Y*(cum), and *Q*^2^(cum) were all above 0.7. Moreover, permutation testing indicated good stability and reliability of the model with an *R*^2^*Y* of >0.7 and a *Q*^2^*Y* of <0.

After filtering and optimizing, a total of 1,637 metabolites (748 and 889 in positive and negative ion modes, respectively) were identified from the 30 rumen fluid samples. Classification information of metabolites was obtained via comparing with the Human Metabolome Database (HMDB) 4.0. As shown in Fig. S7, at the superclass level, metabolites belonging to lipids and lipid-like molecules (28.45%), organic acids and derivatives (19.86%), organoheterocyclic compounds (14.95%), phenylpropanoids and polyketides (14.19%), and organic oxygen compounds (10.43%) were predominant (Fig. S7A). At the subclass level, amino acids, peptides and analogues (16.95%), carbohydrates and carbohydrate conjugates (7.59%), and fatty acids and conjugates (3.99%) were the main components, and 11.81% of metabolites were not identified according to the taxonomic information (Fig. S7B).

### Significantly differentially abundant rumen metabolites and their identification.

**FIG 7:**
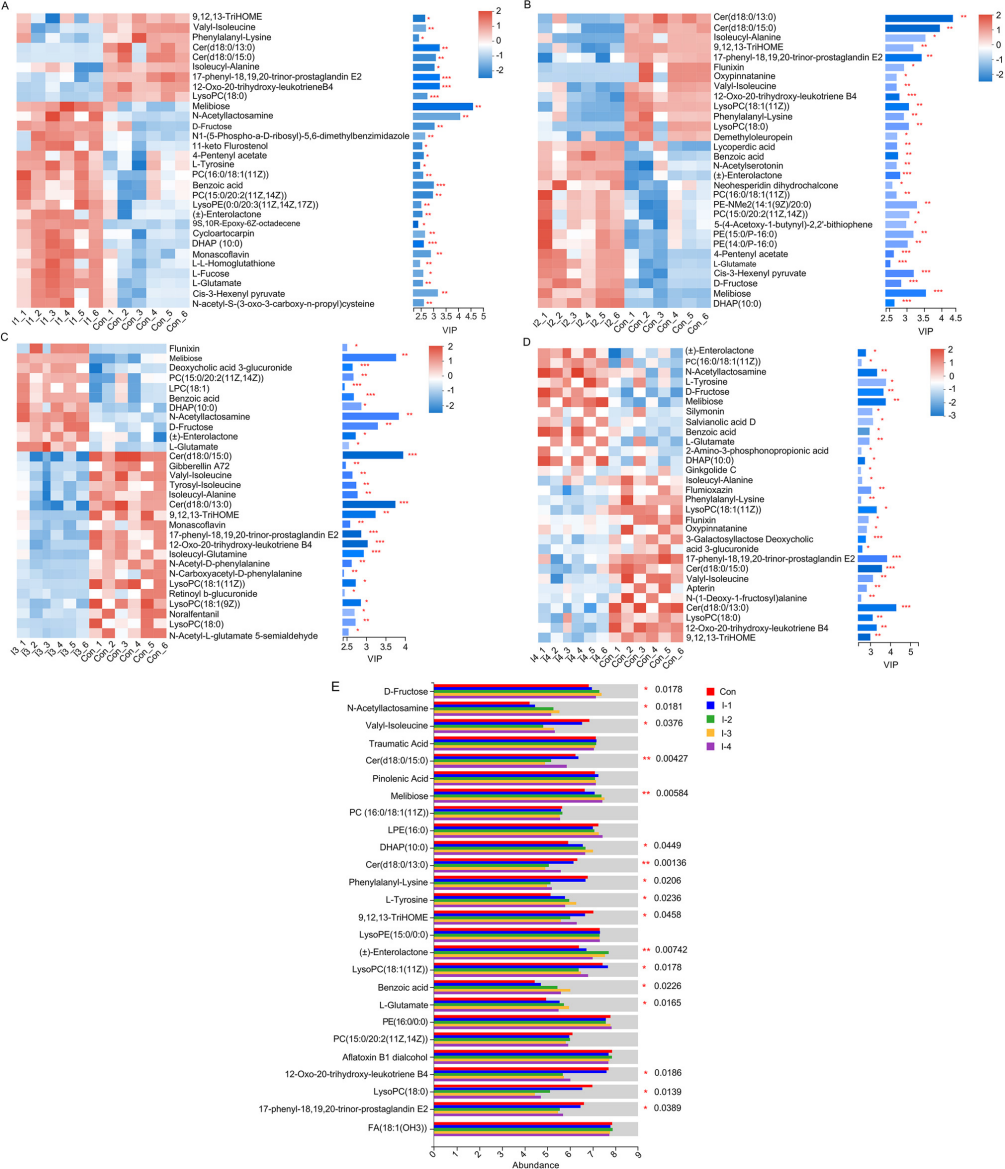
The significantly differentially abundant rumen metabolites between the control and I-1 groups (A), control and I-2 groups (B), control and I-3 groups (C), and control and I-4 groups (D) based on variable important in projection (VIP) values (top 30) and among control and 4 inulin groups (E). (A to D) Each column represents a sample, each row represents a metabolite, each color represents the relative expression of metabolites in the sample, and the corresponding relationship between the color gradient and the value is shown in the gradient color block. VIP bar graphs of metabolites are on the right. The length of the bar represents the contribution value of the metabolite to the difference between the two groups. The larger the value, the greater the difference between the two groups. The color of the bar indicates the significance of the metabolite difference between the two groups, that is, the FDR-adjusted *P* value (*, 0.01 < FDR-adjusted *P* value ≤ 0.05; **, 0.001 < FDR-adjusted *P* value ≤ 0.01; ***, FDR-adjusted *P* value ≤ 0.001). Con, control group; I-1, inulin-1 group with an inulin addition level of 100 g/day per cow; I-2, inulin-2 group with an inulin addition level of 200 g/day per cow; I-3, inulin-3 group with an inulin addition level of 300 g/day per cow; I-4, inulin-4 group with an inulin addition level of 400 g/day per cow.

Differential metabolites with a variable important in projection (VIP) value of >1, a fold change of >1.5 or <0.67, and an FDR-adjusted *P *value of <0.05 among the control group and the 4 inulin groups are listed in Table S5 to S8. The significantly differentially abundant rumen metabolites selected through OPLS-DA in a manner of pairwise comparisons (top 30 of VIP value) between control versus the I-1 group, control versus the I-2 group, control versus the I-3 group, and control versus the I-4 group are shown in Fig. 7A to D. According to the pairwise comparison, the common difference metabolites in the 4 pairs (control versus I-1 group, control versus I-2 group, control versus I-3 group, and control versus I-4 group) were subjected to further multigroup variance analysis through the Kruskal-Wallis H test (Fig. 7E; Table S9). Compared with the control group, the average relative abundances of d-fructose (FDR-adjusted *P = *0.0178), melibiose (FDR-adjusted *P = *0.0058), *N*-acetyllactosamine (FDR-adjusted *P = *0.0181), l-glutamate (FDR-adjusted *P = *0.0163), l-tyrosine (FDR-adjusted *P = *0.0236), benzoic acid (FDR-adjusted *P = *0.0226), dihydroxyacetone phosphate(10:0) (DHAP[10:0]) (FDR-adjusted *P = *0.0449), (±)-enterolactone (FDR-adjusted *P = *0.0074), etc., were increased in the cows fed inulin. Among them, the abundances of d-fructose, melibiose, *N*-acetyllactosamine, phosphatidyl choline (PC)[15:0/20:2(11Z,14Z)], PC[16:0/18:1(11Z)], and (±)-enterolactone were highest in the I-2 and I-3 groups with no significant difference between each other. l-Glutamate, l-tyrosine, benzoic acid, and DHAP(10:0) were most abundant in the I-3 group.

However, the levels of ceramide(d18:0/15:0) [Cer(d18:0/15:0)] (FDR-adjusted *P = *0.0043), Cer(d18:0/13:0) (FDR-adjusted *P = *0.0014), 12-oxo-20-trihydroxy-leukotriene B4 (FDR-adjusted *P = *0.0186), 17-phenyl-18,19,20-trinor-prostaglandin E_2_ (FDR-adjusted *P = *0.0389), lysophosphatidycholine(18:1[11Z]) [LysoPC(18:1[11Z])] (FDR-adjusted *P = *0.0178), 9,12,13-trihydroxy-10-octadecenoic acid (9,12,13-TriHOME) (FDR-adjusted *P = *0.0458), valyl-isoleucine (FDR-adjusted *P = *0.0376), and phenylalanyl-lysine (FDR-adjusted *P = *0.0206) were reduced in the inulin treatment groups compared with the control group. Among the I-2, I-3, and I-4 groups, 17-phenyl-18,19,20-trinor-prostaglandin E_2_, 9,12,13-TriHOME, valyl-isoleucine, and phenylalanyl-lysine demonstrated no significant difference. By contrast, the content of Cer(d18:0/15:0), Cer(d18:0/13:0), 12-oxo-20-trihydroxy-leukotriene B4, and LysoPC(18:1[11Z]) were lower in the I-2 and I-3 groups. The lowest abundance of LysoPC(18:0) was found in the I-3 and I-4 groups.

### Metabolic pathway analysis of differentially abundant metabolites.

The KEGG database was used to annotate the metabolic pathways of differentially abundant metabolites involved (Table 5). The major metabolic pathways that the increased metabolites in inulin groups involved were galactose metabolism, amino acid metabolism and biosynthesis, protein digestion and absorption, the glycolytic pathway, etc. However, sphingolipid metabolism, arachidonic acid metabolism, choline metabolism, glycerophospholipid metabolism, amino acid/peptide hydrolysis, etc., were the main metabolic pathways that the high-abundance metabolites in the control group were involved in.

**TABLE 5:** Metabolic pathway enrichment analysis of significantly differentially abundant rumen metabolites among control and inulin groups

Metabolite^*a*^	KEGG pathway	*P* value	FDR^*a*^
Upregulated in inulin treatment groups			
d-Fructose	Carbohydrate digestion and absorption	0.0001	0.020
	Fructose and mannose metabolism	0.005	0.045
	ABC transporters	0.003	0.046
	Galactose metabolism	0.002	0.023
Melibiose	Galactose metabolism	0.005	0.005
	ABC transporters	0.013	0.048
*N*-Acetyllactosamine	Galactose metabolism	0.007	0.039
DHAP(10:0)	Glycolytic pathway	0.003	0.042
l-Glutamate	Protein digestion and absorption	0.0004	0.013
	Aminoacyl-tRNA biosynthesis	0.0017	0.017
	Alanine, aspartate, and glutamate metabolism	0.005	0.036
	Glutathione metabolism	0.014	0.048
	d-Glutamine and d-glutamate metabolism	0.003	0.024
l-Tyrosine	Prolactin signaling pathway	0.002	0.043
	Thiamine metabolism	0.002	0.030
	Phenylalanine, tyrosine, and tryptophan biosynthesis	0.002	0.038
	Tyrosine metabolism	0.013	0.035
	Aminoacyl-tRNA biosynthesis	0.002	0.017
	Protein digestion and absorption	0.000	<0.001
	Phenylalanine metabolism	0.000	<0.001
Benzoic acid	Phenylalanine metabolism	0.000	<0.001
(±)-Enterolactone	–	0.008	0.048
Downregulated in inulin treatment groups			
Cer(d18:0/15:0)	Sphingolipid metabolism	0.001	0.035
Cer(d18:0/13:0)	Sphingolipid metabolism	0.004	0.042
12-Oxo-20-trihydroxy-leukotriene B4	Arachidonic acid metabolism	0.006	0.036
17-phenyl-18,19,20-trinor-prostaglandin E2	Arachidonic acid metabolism	0.003	0.042
LysoPC(18:1[11Z])	Choline metabolism in cancer	0.002	0.019
	Glycerophospholipid metabolism	0.014	0.036
LysoPC(18:0)	Choline metabolism in cancer	0.002	0.017
	Glycerophospholipid metabolism	0.002	0.038
9,12,13-TriHOME	Linoleic acid metabolism	0.002	0.017
Valyl-isoleucine	Amino acid/peptide hydrolysis	0.014	0.036
Phenylalanyl-lysine	Amino acid/peptide hydrolysis	0.012	0.035

a DHAP, dihydroxyacetone phosphate; PC, phosphatidyl choline; Cer, ceramide; LysoPC, lysophosphatidycholine; ABC transporters, ATP binding cassette transporters; FDR, false-discovery rate; –, unannotated KEGG pathway.

### Correlation analysis between significantly differentially abundant metabolites and ruminal bacteria.

**FIG 8:**
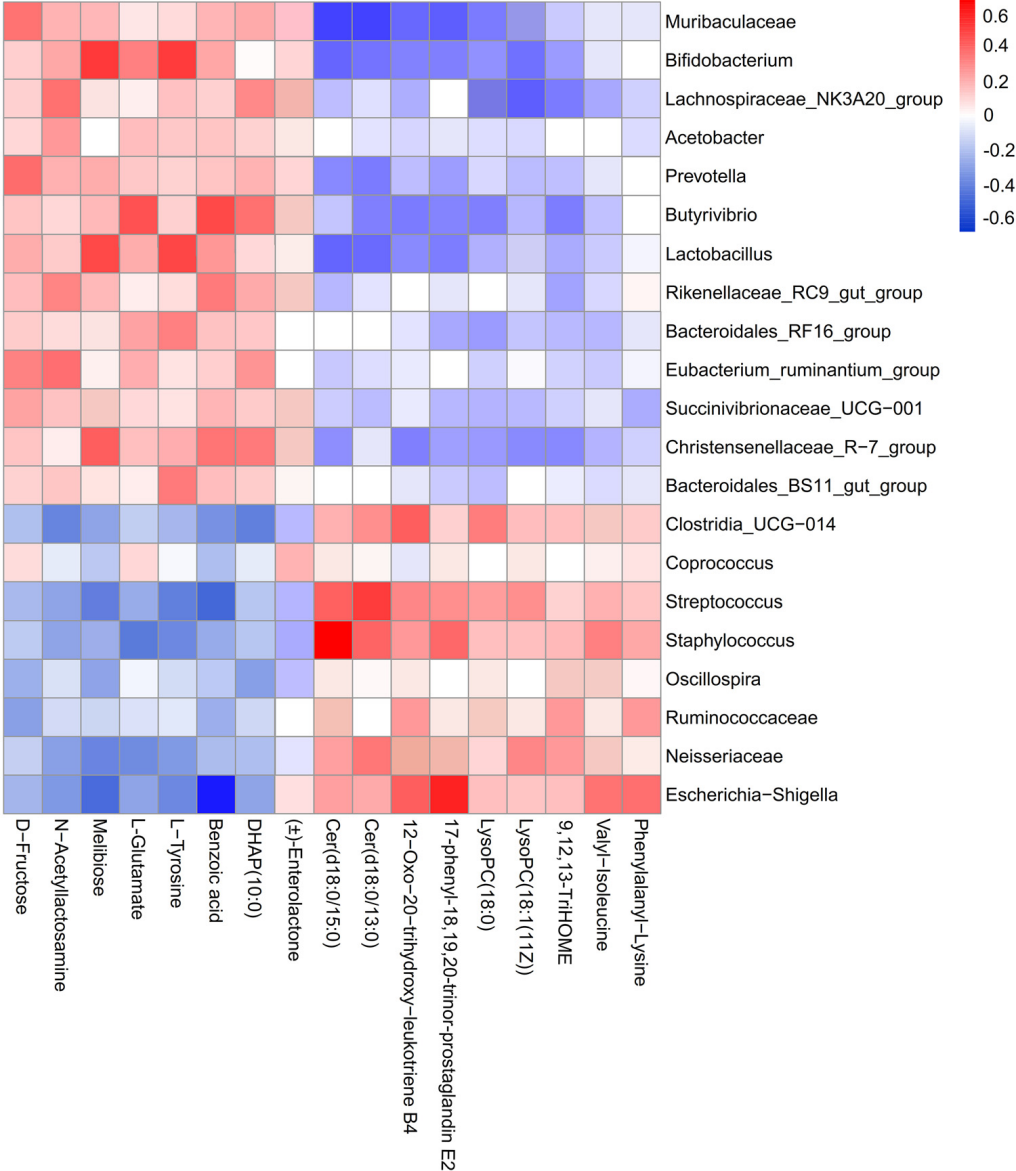
Correlation analysis between significantly differentially abundant ruminal microorganisms and metabolites among control and inulin-treated groups. Each row in the graph represents a genus, each column represents a metabolite, and each lattice represents a Spearman correlation coefficient between a bacterium and a metabolite. Red represents a positive correlation, while blue represents a negative correlation.

Based on Spearman’s correlation coeﬃcients, the correlations between significantly differentially abundant metabolites and bacteria in rumen are shown in Fig. 8, and correlation coefficients (*r*) and FDR-adjusted *P* values are listed in Table S10. Melibiose was positively associated with *Bifidobacterium* (*r* = 0.610, FDR-adjusted *P = *0.014) and *Lactobacillus* (*r* = 0.581, FDR-adjusted *P = *0.035) but negatively associated with Streptococcus (*r* = −0.524, FDR-adjusted *P = *0.036) and Escherichia*-Shigella* (*r* = −0.594, FDR-adjusted *P = *0.025). l-Glutamate was positively correlated with *Butyrivibrio* (*r* = 0.607, FDR-adjusted *P = *0.020) but negatively associated with Staphylococcus (*r* = −0.445, FDR-adjusted *P = *0.048). l-Tyrosine was significantly positively correlated with *Bifidobacterium* (*r* = 0.614, FDR-adjusted *P = *0.031) and *Lactobacillus* (*r* = 0.594, FDR-adjusted *P = *0.027). Benzoic acid was significantly associated with *Butyrivibrio* (*r* = 0.581, FDR-adjusted *P = *0.036) but negatively associated with Escherichia*-Shigella* (*r* = −0.607, FDR-adjusted *P = *0.020). Additionally, Cer(d18:0/15:0) and Cer(d18:0/13:0) were positively correlated with Streptococcus (*r* = 0.562, FDR-adjusted *P = *0.037, and *r* = 0.585, FDR-adjusted *P = *0.035, respectively) and Staphylococcus (*r* = 0.577, FDR-adjusted *P = *0.034, and *r* = 0.484, FDR-adjusted *P = *0.048, respectively). 12-Oxo-20-trihydroxy-leukotriene B4 and 17-phenyl-18,19,20-trinor-prostaglandin E_2_ were mainly positively related to Escherichia*-Shigella* (*r* = 0.540, FDR-adjusted *P = *0.030, and *r* = 0.605, FDR-adjusted *P = *0.030, respectively). Moreover, valyl-isoleucine and phenylalanyl-lysine were also positively correlated with Escherichia*-Shigella* (*r* = 0.541, FDR-adjusted *P = *0.042, and *r* = 0.527, FDR-adjusted *P = *0.047, respectively).

## DISCUSSION

BM is not merely a local inflammatory response that occurs in the udder (7–10, 15). It can a strong immune response throughout the body and changes physiology and behavior, including impaired lactation function, slowed rumen motility, less feeding time, and reduced rumination frequency (16). Currently, little information is available on the effect of inulin on lactation performance in dairy cows. The large amount of volatile fatty acids (VFAs) produced by rumen microbial fermentation of inulin might provide energy substrates for lactation (16, 17). Decreased milk lactose secretion was considered to be one of the indicators of mastitis (18). Increased permeability of the blood-milk barrier caused by inflammation promotes certain ions entering the milk from the blood, which might cause an osmotic gradient that is not conducive to the secretion of milk lactose (18). However, as the precursor of lactose synthesis, the increased concentration of propionate produced by inulin fermentation in the rumen might promote the synthesis of milk lactose (19). Additionally, inulin increased the diversity and richness of rumen microbes in this study; thus, the elevated synthesis of microbial protein in rumen may contribute to the synthesis of milk protein in the mammary gland (20). Further evidence for the increase of rumen microbial protein content after inulin supplementation was the decrease of rumen NH_3_-N concentration. Consistent with previous studies, inulin can be used as a substrate by hemicellulose-degrading bacteria in rumen to support their growth and nitrogen utilization, so reduced NH_3_-N concentrations have been observed in rumen with inulin supplementation (21–23). Moreover, Gressley et al. (23) found that the concentrations of MUN and BUN in lactating dairy cows were decreased after inulin supplementation, which also reflected the reduction of rumen NH_3_-N. The concentrations of RUN, MUN, and BUN could reflect the utilization of protein in the rumen (23). However, RUN only presented a downward trend after inulin treatment in our experiment.

During mastitis, the transport of LPS from the rumen to blood could increase the release of proinflammatory cytokines and elevate the content of immunoglobulin in the blood (24, 25). In the present study, inulin treatment reduced the concentration of LPS in rumen and blood. It’s reported that inulin supplementation could decrease gut permeability and reduce LPS translocation to ameliorate systematic inflammation (26), but direct evidence of gut permeability and integrity still needs further research. Additionally, decreased levels of proinflammatory factors (PGE_2_, IL-6, IL-8, and TNF-α) and increased levels of the anti-inflammatory factor IL-4 might indicate that inulin supplementation could alleviate inflammation via reducing proinflammatory cytokines (26). Meanwhile, it might also explain the reduced levels of milk SCC in the inulin groups.

For ruminants, the rumen is the dominant organ housing the largest number of microorganisms, which directly affects the digestion and metabolism of nutrients and immune function (27). Previous studies reported that rumen microbial diversity and concentration of SCFAs in cows with SCM were significantly reduced, which might be due to the changed structure in the rumen microbial community (8, 15). Consistent with previous studies, inulin supplementation significantly increased the concentration of TVFAs in the rumen of dairy cows with SCM, especially butyrate, propionate, and LA. The concentration of acetate did not change significantly, and A/P decreased significantly (27, 28). Acetate is regarded as the precursor of milk fat synthesis (29); thus, milk fat was also not observed to be significantly changed after inulin treatment in the current study.

Changes in concentration of SCFAs were closely related to the inulin effect on the ruminal bacteria profile. In the present study, supplementation of inulin significantly increased diversity and richness of the rumen microbiome. Inulin could be converted to microbial glycogen by ruminal bacteria and protozoa, which would affect VFAs and microbial production (30). At the phylum level, inulin addition increased the relative abundance of *Bacteroidota* and *Proteobacteria* yet decreased *Firmicutes* and *Actinobacteriota*. *Bacteroidota*, such as *Prevotella*, *Bacteroidales* BS11 gut group, *Bacteroidales* RF16 group, and *Muribaculaceae*, produce mainly propionate by fermenting dietary fiber (31–33). *Muribaculaceae* was the member of the beneficial commensal bacteria in the rumen and intestine, and participated in the formation of the mucus layer, maintaining of barrier function and being related to the innate immune system (34). Zhong et al. (15) found that compared with high-SCC dairy cows, *Proteobacteria* were enriched in the rumen of cows with low SCC, among which *Succinivibrionaceae* were the main cause of the difference. This was consistent with our findings. *Succinivibrionaceae* are also succinate-producing bacteria (35). Thus, the above increased propionate-producing bacteria in inulin groups might explain the positive correlation with milk lactose (19). Importantly, several studies suggested that propionate plays an important role in hosts’ susceptibility to bacterial and fungal infections (9, 36) and could prevent LPS-induced mastitis by regulating the blood-milk barrier (11).

*Firmicutes*, such as *Butyrivibrio*, *Lachnospiraceae* NK3A20 group, *Lactobacillus*, and Eubacterium ruminantium group, could effectively ferment inulin to produce butyrate and LA (31, 37). In addition, inulin could promote the proliferation of *Bifidobacterium* in the gastrointestinal tract, which may inhibit the secretion of IL-8 and stimulate the production of IL-10 (38). Although *Bifidobacterium* could not directly produce butyrate through fermentation of inulin, it could stimulate other butyrate-producing bacteria, such as the Eubacterium ruminantium group (39). Butyrate was the crucial nutrient inducing the expression of tight junction proteins and could affect the inflammatory response by regulating the release of inflammatory cytokines (40). Meanwhile, LA produced by *Lactobacillus* through fermentation of inulin could prevent the invasion and colonization of pathogens in the intestinal tract (41).

As the common pathogens in milk of cows with mastitis, Streptococcus, Staphylococcus, Escherichia*-Shigella*, and the conditional pathogen *Clostridia* have also been identified in the rumen of cows with SCM in this study. Recently, the entero-mammary endogenous pathway of microorganisms has been increasingly reported (10). Gastrointestinal microorganisms have been demonstrated to affect inflammation in distal intestinal tissues, including the udder (4–6). Studies reported that changes in fecal microbial communities of cows with mastitis were similar to those in the milk, with increases in *Enterococcus*, Streptococcus, and Staphylococcus and decreases in *Lactobacillus* (7, 10). Consistent with Wang et al. (8), the conditional pathogen *Neisseriaceae* in the intestine was significantly enriched in the rumen of cows with SCM. An important virulence mechanism of *Neisseriaceae* is the ability to shed outer membrane blebs containing LPS (42). In this study, we observed higher abundance of *Ruminococcaceae* and *Oscillospira* in the rumen of cows with SCM. Ma et al. (7) also found that these two bacteria at high levels in the feces of cows with mastitis. The upregulated *Ruminococcaceae* and *Oscillospira* in the rumen of cows with mastitis might be related to infection and inflammation, which still needs to be further explored. In this study, these inflammation-related ruminal bacteria were positively correlated with milk SCC and negatively correlated with milk components, highlighting their adverse effect on lactation performance.

Correlations between the rumen microbial profile and mastitis have been confirmed by several studies (7–9, 15). In the current study, dietary supplementation of inulin mainly increased the abundance of propionate- and butyrate-producing bacteria and beneficial commensal bacteria in the rumen of cows with SCM and reduced the abundance of inflammation-inducing bacteria within the rumen. Consistent with Li et al. (26), our results suggest that the anti-inflammation effect of inulin might closely relate to the modulation of ruminal microbiota.

Variations in the rumen microbial community might further lead to changes in metabolic activities. The increase in d-fructose, melibiose, *N*-acetyllactosamine, and DHAP(10:0) after inulin addition upregulated a series of metabolic pathways related to energy metabolism, such as carbohydrate digestion and absorption, galactose metabolism, fructose and mannose metabolism, and the glycolytic pathway. Xi et al. (1) detected a significant decrease in carbohydrates and energy-related metabolites in milk at the peak of IMI. Ma et al. (7) found a consumed glycolytic metabolism pathway in the fecal metabolites of cows with mastitis. In addition, the inflammatory response caused by pathogens in the rumen might lead to local energy consumption (1, 7). Inulin supplementation elevated the energy levels of cows with SCM, which was essential for maintaining the synthesis of milk components and lactation. Of note, melibiose is also reported to participate in immune responses, which could strongly affect the response of Th cells to ingested antigens (43). Moreover, the increase of l-glutamate, l-tyrosine, and benzoic acid in the inulin groups facilitated glutathione metabolism, tyrosine metabolism, phenylalanine metabolism, protein digestion and absorption, thiamine metabolism. Elevated amino acid metabolism in the rumen of cows with SCM fed inulin might be attributed to the degradation of increased rumen microbial protein. The thiamine metabolic pathway was also previously observed to be inhibited in the feces and milk of dairy cows with mastitis (7, 44). As a cofactor for many biochemical reactions, vitamin B might participate in the inhibition of inflammation (7). Furthermore, in the gastrointestinal tract amino acids could be used by microbiota activated by inulin to synthesize SCFAs (45). For instance, butyrate could be derived from glutamate, which could maintain intestinal barrier function (46). During mastitis, tyrosine and phenylalanine are also decreased in milk (1). l-Tyrosine could be produced by *Bifidobacterium* and *Lactobacillus* (1). Benzoic acid is the precursor of hippuric acid, which inhibits the growth of E. coli (1, 47).

On the other side, the downregulated pathways were mainly linked to lipid metabolism, including sphingolipids, arachidonic acid, choline metabolism, and glycerophospholipid metabolism. Decreased lipid metabolites contained ceramide, 17-phenyl-18,19,20-trinor-prostaglandin E_2_, 12-oxo-20-trihydroxy-leukotriene B4, LysoPC, and 9,12,13-TriHOME, which may be involved in the host proinflammatory response. Specifically, ceramide is a sphingolipid inflammatory mediator that participates in LPS-mediated mitochondrial destruction to induce apoptosis (48). 17-Phenyl-18,19,20-trinor-prostaglandin E_2_ was classified as a member of the prostaglandins and related compounds (49). PGE_2_ could accelerate E. coli-induced mastitis in dairy cows during early lactation (49). Moreover, in the present study, a high concentration of PGE_2_ was also detected in the serum of cows with SCM after inulin treatment. 12-Oxo-20-trihydroxy-leukotriene B4, LysoPC, and 9,12,13-TriHOME were all capable of aggregating neutrophils. 12-Oxo-20-trihydroxy-leukotriene B4 was the lipid omega-oxidation metabolite of leukotriene B4 (LTB4), which was the main metabolite of neutrophil polymorphonuclear leukocytes. During BM, LTB4 could induce the infiltration of neutrophils in the bovine mammary gland and lead to a surge in SCC (50). Despite the fact that LysoPC and 9,12,13-TriHOME were reported to induce the recruitment of monocytes and the production of proinflammatory cytokines, limited information regarding their association with BM has been reported (51, 52).

At present, limited studies have investigated the effect of inulin on these lipid proinflammatory metabolites in the rumen of cows with SCM. We speculated that it might be related to the regulation of lipid metabolism by inulin. Consistent with those studies on humans and other animals, consumption of inulin could cut down the concentration of triglycerides and cholesterol, which is mediated through the reduced activities of hepatic lipogenic enzymes, such as ATP citrate lyase, acetyl coenzyme A (acetyl-CoA) carboxylase, and fatty acid synthase (12, 13).

Based on the correlation between BM and the rumen inner environment, modulating the microbial community and the abundance of metabolites in the rumen may contribute to SCM remission to a certain extent. Throughout the study, modulation of inulin in the microbial community and metabolites in rumen affected the level of inflammatory cytokines and LPS as well as milk composition in cows with SCM. Moreover, during the period of SCM, increased milk SCC indicates elevated neutrophils in milk which result in oxidation reactions and the increase of liquid peroxidation products (53). In the present study, the antioxidant activity of inulin might relate to the scavenging of reactive oxygen species and hydroxyl free radicals (54), which need further study. On the other hand, the results of lactation performance, immunity indices, rumen fermentation, and microbial diversity in cows with SCM did not increase linearly with an increase of inulin supplementation in the present study. When the addition level was over 300 g/day per cow, most of the indicators showed a downward trend, which might be due to the metabolic adaptability of dairy cows to inulin. Currently, limited information is available on the appropriate dosages of inulin in the diets of dairy cows. Overall, in the current study, dietary supplementation of 300 g/day of inulin per cow has a better effect in alleviating inflammation reactions in cows with SCM.

In summary, our findings revealed that dietary supplementation of inulin could increase the abundance of propionate- and butyrate-producing bacteria and beneficial commensal bacteria, promote the level of metabolites associated with energy and amino acid metabolism, and lower the abundance of inflammation-related bacteria and levels of lipid proinflammatory metabolites in the rumen of cows with SCM. Thereby, inulin attenuates inflammatory and oxidative stress responses in the serum, increases the concentration of propionate and butyrate in the rumen, and improves the lactation performance of cows with SCM. Overall, the mechanism of inulin in alleviating SCM in dairy cows might be through the improvement of the rumen microbial community and their metabolites.

## MATERIALS AND METHODS

### Ethics statement.

All experimental designs and protocols were approved by the Animal Ethics Committee of the Chinese Academy of Agricultural Sciences (Beijing, China; approval number IAS-2020-92) and were in accordance with the recommendations of the academy’s guidelines for animal research.

### Animals, feedstuffs, and experiment design.

The current study was conducted in an intensively managed dairy farm located in Beijing, China (39°91′N, 116°66′E). According to milk somatic cell counts (SCC), California mastitis test (CMT), milk electric conductivity (EC), and clinical symptoms in udders (55) and by combining with the basic body condition (milk SCC = 684,000 ± 27.7 cells/ml; the result of CMT was weakly positive and positive; milk EC = 7.83 ± 1.07; no clinical symptoms in udders; days in milk = 137 ± 8.7 days; parity = 3.43 ± 0.38; milk yield = 29.7 ± 1.96 kg/day), a total of 30 Holstein dairy cows with SCM in mid-lactation were selected and randomly divided into 5 groups (*n* = 6). Inulin addition levels of 0 (control group), 100 (I-1 group), 200 (I-2 group), 300 (I-3 group), and 400 (I-4 group) g/day per cow, respectively, were used. The criteria for CMT results are referenced in Wang et al. (8). Inulin used in the current study was supplied by Langfang Academy of Agriculture and Forestry Sciences (Hebei, China). All groups were fed the same total mixed rations (TMR) as basic diet, with a concentrate-to-forage ratio of 40:60. The TMR ingredients and nutritional compositions are shown in Table S1 in the supplemental material. Cows were housed in individual tie stalls and offered TMR *ad libitum* 3 times a day at 07:30, 13:30, and 19:30 with free access to food and water. Due to the hygroscopicity of inulin, it was weighed and rolled into pills with the same shape and size. A feeder (Boehringer-Ingelheim, Biblach, Germany) was used to dose the pills via the mouth 3 times a day at the time of offering TMR. Feed residuals were collected to calculate dry matter intake (DMI). The experiment lasted for 8 weeks, with 2 weeks for adaptation and 6 weeks for the trial.

### Milk sampling and analysis.

Cows were milked 3 times a day at 06:30, 12:30, and 18:30 through an automatic milking system (Afimilk Ltd., Kibbutz Afikim, Israel). Milk yield data were recorded every day by a dairy herd management software program (Afimilk Ltd., Kibbutz Afikim, Israel). The milk yield reported was the average of each week. Milk samples from each cow were sampled in the morning, at 12:00, and in the evening and mixed with a ratio of 4:3:3 on the last day of the experiment (day 56) (56). Mixed milk samples from each cow were collected into two 50-ml sterile centrifuge tubes and stored at 4°C. Milk composition, including milk fat, milk protein, lactose, and SCC, was determined by near infrared spectroscopy using a milk composition analyzer (PRO40SEC, Daimaker Technology Co., Ltd., Beijing, China). The milk EC was measured by a conductivity meter (MIK-EC8.0, Meikong Automation Technology Co., Ltd., Hangzhou, China). The concentration of milk urea nitrogen (MUN) was analyzed using a MUN test kit (SK081, Bio Lebo Technology Co., Ltd., Beijing, China).

### Serum collection and analysis.

Blood sampling was conducted 1h before morning feeding on the last day of the experiment (day 56). Two tubes of blood samples (5 ml per tube) from each cow were sampled through the tail vein. The blood samples were placed on ice for 30 min. After coagulating, the blood samples were centrifuged for 15 min at 3,000 × *g* at 4°C to separate the serum. The serum sample from each cow was collected and stored at −20°C for the further detection of blood urea nitrogen (BUN), total protein (TP), albumin (ALB), globulin (GLB), inflammatory cytokines, and lipopolysaccharide (LPS). BUN was analyzed using a BUN test kit (Meilian Biotechnology Co., Ltd., Shanghai, China). The concentrations of TP and ALB were analyzed by a TP colorimetric test kit (biuret method) (Elarite Biological Technology Co., Ltd., Wuhan, China) and an ALB assay kit (bromocresol green colorimetry) (Ameijie Technology Co., Ltd., Wuhan, China), respectively. The concentration of GLB was calculated as the difference between the TP and ALB. The concentration of IL-6, IL-8, IL-10, IL-4, IL-2, TNF-α, prostaglandin E_2_ (PGE_2_), glutathione peroxidase (GSH-Px), superoxide dismutase (SOD), and malondialdehyde (MDA) were measured by corresponding bovine enzyme-linked immunosorbent assay (ELISA) kits (Enzyme Link Biotechnology Co. Ltd., Shanghai, China). The concentrations of IgA, IgG, and IgM were determined by immunoturbidimetry with bovine immunoglobulin ELISA kits (Cloud-Clone Technology Co., Ltd., Wuhan, China). The concentration of LPS in serum was detected by a general LPS ELISA kit (ZY-LPS-Ge, Ze Ye Biotechnology Co., Ltd., Shanghai, China). The above measurements were acquired according to the manufacturers’ instructions.

### Rumen fluid sampling and measurement.

On day 56, ruminal fluid samples from each cow were sampled 3 h after morning feeding using a rumen fluid sampler with a 1.5-m hose and a metal filter (GCYQ-1-A, Shanghai SDI Scientific Instrument Co., Ltd., Shanghai, China) and a 200-ml syringe. Ruminal fluid was drawn with the syringe by inserting the rumen sampler through the mouth into the rumen. The first two tubes of ruminal fluid were discarded to prevent saliva contamination. Approximately 150 ml of rumen fluid from each cow was sampled. Four layers of sterile gauze were used to filter the rumen fluid samples. The pH value of the filtrate was measured immediately using a portable pH meter (WLB-TC-PH, Wailaibo Biotechnology Co., Ltd., Beijing, China). The filtrate from each cow was divided into five 10-ml sterile centrifuge tubes to determine the concentrations of volatile fatty acids (VFAs), lactic acid (LA), NH_3_-N, rumen urea nitrogen (RUN), LPS, and ruminal microorganisms and metabolites. The ruminal fluid samples used to analyze the microbiome and metabolites were stored at −80°C, and the others were stored at −20°C. The concentrations of VFAs, including acetate, propionate, butyrate, isobutyrate, valerate, and isovalerate, were analyzed by gas chromatography (Agilent 7890A, Agilent Technologies, CA, USA). The concentration of NH_3_-N was detected via alkaline sodium hypochlorite-phenol spectrophotometry. The concentration of LA was measured by a lactate assay kit (MAK064, Sigma-Aldrich, St. Louis, MO, USA). The concentration of RUN was measured by the diacetyl-anthracene colorimetric method using a urea test kit (CJT77101, Chejeter Technology CO., Ltd., Beijing, China). The concentration of LPS in rumen was detected by a general LPS ELISA kit (ZY-LPS-Ge, Ze Ye Biotechnology Co., Ltd., Shanghai, China) (17). Analysis of the ruminal microbiota and metabolites was performed by 16S rRNA sequencing and untargeted metabolomics based on liquid chromatography-mass spectrometry (LC-MS), respectively (8).

### DNA extraction, PCR amplification, and sequencing.

Rumen microbiome genomic DNA from the 30 samples was extracted according to the instructions of the FastDNA spin kit for soil (MPbio, CA, USA) in accordance with the manufacturer’s instructions, and the details were referred to in our previous study (9). To extract total DNA, 1 ml of rumen fluid, 978 μl of sodium phosphate buffer, and 122 μl of MT buffer were added to the lysing matrix E tube, which was centrifuged at 14,000 rpm for 10 min. The supernatant was transferred to a 1.5-ml centrifuge tube, and 250 μl of polyphenylene sulfide (PPS) was added, which was mixed well and centrifuged at 14,000 rpm at ambient temperature for 5 min. The supernatant was transferred into a 2-ml tube with 900 μl of binding matrix and mixed thoroughly, then 500 μl of 5.5 M guanidine isothiocyanate solution was added. The solution was transferred to a SPIN filter and 500 μl of SEWS-M was added, which was then centrifuged at 14,000 rpm for 1 min. The residual solution was removed and dried for 3 min. After adding 100 μl of 55°C preheated DES (DNase/pyrogen free water) eluate for 5 min, the solution was centrifuged at 14,000 rpm at ambient temperature for 2 min. Total DNA was obtained after discarding the SPIN filter. The purity and concentration of DNA were detected by a spectrophotometer (NanoDrop2000, Thermo Fisher Scientific, Waltham, USA). DNA integrity was determined by 1% agarose gel electrophoresis at 5 V/cm for 20 min.

The primer pairs 338F (5′-ACTCCTACGGGAGGCAGCAG-3′) and 806R (5′-GGACTACHVGGGT WTCTAAT-3′) targeting the hypervariable region V3-V4 of the ruminal bacterial 16S rRNA gene were used to conduct PCR amplification. The PCR system and program were the same as in our previous study (8). PCRs were performed in triplicate. The PCR products were detected by 2% agarose gel electrophoresis. Purification and quantification of PCR products were performed using an AxyPrep DNA gel extraction kit (Axygen Biosciences, Union City, CA, USA) and a Quantus fluorometer (Promega, Madison, USA), respectively. Subsequently, the libraries were processed using the purified amplicons using a NEXTFLEX rapid DNA-seq kit (Bioo Scientific, USA) and were further sequenced on a MiSeq PE300 platform (Illumina, San Diego, CA, USA) (57).

### Processing of sequencing data.

Quality control (QC) for the raw sequencing data was performed using Trimmomatic software (version 0.39). Paired-end reads were merged using FLASH (V1.2.11, http://ccb.jhu.edu/software/FLASH/). The Uparse software (version 7.0.1090 http://www.drive5.com/uparse/) was used for operational taxonomic unit (OTU) clustering: (i) extracting nonrepetitive sequences from optimized sequences (http://drive5.com/usearch/manual/dereplication.html); (ii) removing single sequences without repetitions (http://drive5.com/usearch/manual/singletons.html); (iii) performing OTU clustering for nonrepetitive sequences (excluding single sequences) according to 97% similarity, removing chimeras, and obtaining representative sequences of OTUs; and (iv) selecting sequences with >97% similarity to the representative sequence and generating OTU form. The RDP classifier (version 2.11) Bayesian algorithm (https://sourceforge.net/projects/rdp-classifier/) was used to perform taxonomic analysis for OTU representative sequences with greater than 97% similarity. The microbial community composition was counted at different taxonomic levels, which were then compared with the 16s rRNA database Silva (release 132, http://www.arb-silva.de). Alpha diversity indexes were calculated by Mothur software (version 1.30.2, http://www.mothur.org/wiki/Calculators). Based on the alpha diversity indexes (Shannon and Sobs), the rarefaction curves were drawn using the vegan package in R software (version 2.12.1). The distance algorithms of the principal coordinates analysis (PCoA) were weighted normalized UniFrac and unweighted UniFrac, with analysis of similarities (ANOSIM) as difference tests among groups. According to the relative abundance of ruminal bacteria, a Kruskal-Wallis H test was used to conduct significance tests of differences among groups at phylum and genus levels, respectively. The *P* value was corrected for false-discovery rate (FDR). Hierarchical clustering analysis (HCA) of bacteria with a top 50 relative abundance was conducted using Scipy packages of Python (Version1.0.0), and the clustering manner was average (58).

### LC-MS metabolomics analysis.

Prior to LC-MS, the rumen fluid samples were thawed and mixed thoroughly. The sample preparation steps were consistent with our previous research (10). The supernatant was transferred to an autosampler vial for LC-MS analysis. In addition, 20 μl of supernatant was transferred from each sample in each group to mix as a QC sample in this group. The platform for LC-MS analysis was the UHPLC-Q Exactive HF-X system (Thermo Fisher, MA, USA). Each rumen fluid sample (10 μl) was separated by an Acquity UPLC HSS T3 chromatographic column (100 mm × 2.1 mm inside diameter [i.d.], 1.8 μm; Waters, Milford, USA) to perform mass spectrometry detection. The following chromatographic conditions were used: mobile phase A, 95% water + 5% acetonitrile, containing 0.1% formic acid; mobile phase B, 47.5% acetonitrile + 47.5% isopropanol + 5% water, containing 0.1% formic acid. The following separation gradient parameters were used: 0 to 3.5 min, mobile phase A was decreased from linear 100% to 75.5%, mobile phase B was increased from linear 0% to 24.5%; 3.5 to 5 min, mobile phase A was decreased from linear 75.5% to 35%, mobile phase B was linearly risen from 24.5% to 65%; 5 to 5.5 min, mobile phase A was decreased from linear 55% to 0%, mobile phase B was linearly risen from 65% to 100%; 5.5 to 7.4 min, mobile phase A was maintained at 0%, mobile phase B was maintained at 100%; 7.4 to 7.6 min, mobile phase A was linearly risen from 0% to 48.5%, mobile phase B was linearly decreased from 100% to 51.5%; 7.6 to 10 min, mobile phase A was linearly risen from 48.5% to 100% and maintained for 2.2 min, mobile phase B was linearly decreased from 51.5% to 0% and maintained for 2.2 min. The flow rate was 0.40 ml/min, injection volume was 2 μl, and the column temperature was 40°C. The samples were ionized by electrospray, and mass spectrometry signals were collected in positive and negative ion scanning modes, respectively. The following mass spectrometry conditions were used: mass scan range (*m*/*z*), 70 to 1,050; heater temperature, 425°C; capillary temperature, 352°C; aux gas flow rate, 13 arbitrary units (arb). The spray voltages for positive and negative ion mode were 3,500 and −3,500 V, respectively, and the cyclic collision energy was 20 to 60 eV.

### Data processing in metabolomics.

The LC-MS raw data were imported into the metabolomics processing software Progenesis QI (Waters Corporation, Milford, USA) for baseline filtering, peak identification, integration, retention time correction, peak alignment, etc., and a data matrix, including the information of retention time, mass-to-charge ratio, and peak intensity were obtained. The sum normalization method was used to normalize the response intensity of the samples’ mass spectrum peaks to obtain a normalized data matrix. Meanwhile, the MS and MS-MS data were annotated with the public metabolism database HMDB (http://www.hmdb.ca/) to obtain information about the metabolites.

The R package ropls (version1.6.2) was used to perform principal-component analysis (PCA), partial least squares discriminant analysis (PLS-DA), and orthogonal least partial squares discriminant analysis (OPLS-DA). The 7-fold cross validation and response permutation testing were conducted to evaluate the predictive ability and stability of the model. In addition, Student’s *t* test and multiple of difference analysis were used for difference testing. The selection of significantly different metabolites between two groups was determined based on the variable weight value (VIP) obtained from the OPLS-DA model and the Student’s *t* test *P* value. The *P* value was corrected by FDR adjustment. The metabolites with a VIP value of >1 and an FDR-adjusted *P *value of <0.05 were defined as significantly different metabolites. The clustering and distance calculations for metabolites were analyzed by hierarchical clustering and Euclidean distance, respectively. The selection of significantly different metabolites among the five groups was accomplished through a Kruskal-Wallis H test. Metabolic pathway annotation was performed by using the KEGG database (https://www.kegg.jp/kegg/pathway.html). Spearman’s correlation analysis was performed using Scipy packages of Python (version1.0.0). The *P* value was corrected by FDR adjustment. FDR-adjusted *P* values of <0.05 signified a significant correlation. The range of coefficient of association (*r*) was from −1.0 to 1.0, and an *r* of >0 or an *r* of <0 represented a positive and negative correlation, respectively (59).

### Statistical analysis.

DMI, lactation performance, rumen fermentation parameters, inflammatory cytokines, and oxidative stress indexes data in serum were analyzed by one-way analysis of variance (ANOVA) and Student’s *t* test in SPSS statistics 22 (IBM, New York, USA). Significance was declared at a *P *value of ≤0.05; a tendency was declared at 0.05 < *P *<* *0.10.

### Data availability.

16S rRNA raw data from the study were deposited in the NCBI Sequence Read Archive (SRA) under accession no. PRJNA746433.

## References

[1] Xi X, Kwok LY, Wang Y, Ma C, Mi Z, Zhang H. 2017. Ultra-performance liquid chromatography-quadrupole-time of flight mass spectrometry MS^E^-based untargeted milk metabolomics in dairy cows with subclinical or clinical mastitis. J Dairy Sci 100:4884–4896. doi:10.3168/jds.2016-11939. 28342601

[2] Youssif NH, Hafiz NM, Halawa MA, Saad MF. 2019. Influence of some hygienic measures on the prevalence of subclinical mastitis in a dairy farm. Int J Dairy Science 15:38–47. doi:10.3923/ijds.2020.38.47.

[3] Batavani RA, Asri S, Naebzadeh H. 2007. The effect of subclinical mastitis on milk composition in dairy cows. Iran J Vet Res 8:205–211. doi:10.22099/IJVR.2007.925.

[4] Kamada N, Seo S-U, Chen GY, Núñez G. 2013. Role of gut microbiota in immunity and inflammatory disease. Nat Rev Immunol 13:321–335. doi:10.1038/nri3430. 23618829

[5] Honda K, Littman DR. 2012. The microbiome in infectious disease and inflammation. Annu Rev Immunol 30:759–795. doi:10.1146/annurev-immunol-020711-074937. 22224764 PMC4426968

[6] Littman D, Pamer E. 2011. Role of the commensal microbiota in normal and pathogenic host immune responses. Cell Host Microbe 10:311–323. doi:10.1016/j.chom.2011.10.004. 22018232 PMC3202012

[7] Ma C, Sun Z, Zeng B, Huang S, Zhao J, Zhang Y, Su X, Xu J, Wei H, Zhang H. 2018. Cow-to-mouse fecal transplantations suggest intestinal microbiome as one cause of mastitis. Microbiome 6:200. doi:10.1186/s40168-018-0578-1. 30409169 PMC6225715

[8] Wang Y, Nan X, Zhao Y, Jiang L, Wang M, Wang H, Zhang F, Xue F, Hua D, Liu J, Yao J, Xiong B. 2021. Rumen microbiome structure and metabolites activity in dairy cows with clinical and subclinical mastitis. J Anim Sci Biotechnol 12:36. doi:10.1186/s40104-020-00543-1. 33557959 PMC7869221

[9] Hu X, Guo J, Zhao C, Jiang P, Maimai T, Yanyi L, Cao Y, Fu Y, Zhang N. 2020. The gut microbiota contributes to the development of *Staphylococcus aureus*-induced mastitis in mice. ISME J 14:1897–1910. doi:10.1038/s41396-020-0651-1. 32341472 PMC7305118

[10] Addis MF, Tanca A, Uzzau S, Oikonomou G, Bicalho RC, Moroni P. 2016. The bovine milk microbiota: insights and perspectives from -omics studies. Mol Biosyst 12:2359–2372. doi:10.1039/c6mb00217j. 27216801

[11] Aschenbach JR, Penner GB, Stumpff F, Gäbel G. 2011. Ruminant nutrition symposium: role of fermentation acid absorption in the regulation of ruminal pH. J Anim Sci 89:1092–1107. doi:10.2527/jas.2010-3301. 20952531

[12] Khuenpet K, Jittanit W, Sirisansaneeyakul S, Srichamnong W. 2017. Inulin powder production from Jerusalem artichoke (*Helianthus tuberosus* L.) tuber powder and its application to commercial food products. J Food Process Preserv 41:e13097. doi:10.1111/jfpp.13097.

[13] Samanta AK, Jayapal N, Senani S, Kolte AP, Sridhar Manpal. 2013. Prebiotic inulin: useful dietary adjuncts to manipulate the livestock gut microflora. Braz J Microbiol 44:1–14. doi:10.1590/S1517-83822013005000023. 24159277 PMC3804171

[14] Bergman EN. 1990. Energy contributions of volatile fatty acids from the gastrointestinal tract in various species. Physiol Rev 70:567–590. doi:10.1152/physrev.1990.70.2.567. 2181501

[15] Zhong Y, Xue M, Liu J. 2018. Composition of rumen bacterial community in dairy cows with different levels of somatic cell counts. Front Microbiol 9:3217. doi:10.3389/fmicb.2018.03217. 30619238 PMC6312127

[16] Yeiser EE, Leslie KE, Mcgilliard ML, Petersson-Wolfe CS. 2012. The effects of experimentally induced *Escherichia coli* mastitis and flunixin meglumine administration on activity measures, feed intake, and milk parameters. J Dairy Sci 95:4939–4949. doi:10.3168/jds.2011-5064. 22916898

[17] Yun SK, Han JD. 1989. Effect of feeding frequence of concentrate to milking cow in early lactation on pH and VFA-concentration in rumen fluid and on milk composition and milk yield. Asian-Australas J Anim Sci 2:418–420. doi:10.5713/ajas.1989.418.

[18] Fetherston CM, Lai CT, Mitoulas LR, Hartmann PE. 2006. Excretion of lactose in urine as a measure of increased permeability of the lactating breast during inflammation. Acta Obstet Gynecol Scand 85:20–25. doi:10.1080/00016340500324514. 16521675

[19] Lemosquet S, Rigout S, Bach A, Rulquin H, Blum JW. 2004. Glucose metabolism in lactating cows in response to isoenergetic infusions of propionic acid or duodenal glucose. J Dairy Sci 87:1767–1777. doi:10.3168/jds.S0022-0302(04)73332-9. 15453491

[20] Xue M, Sun H, Wu X, Guan L, Liu J. 2019. Assessment of rumen bacteria in dairy cows with varied milk protein yield. J Dairy Sci 102:5031–5041. doi:10.3168/jds.2018-15974. 30981485

[21] Tian K, Liu J, Sun Y, Wu Y, Chen J, Zhang R, He T, Dong G. 2019. Effects of dietary supplementation of inulin on rumen fermentation and bacterial microbiota, inflammatory response and growth performance in finishing beef steers fed high or low-concentrate diet. Anim Feed Sci Technol 258:114299. doi:10.1016/j.anifeedsci.2019.114299.

[22] Öztürk H. 2008. Effects of inulin on rumen metabolism in vitro. Ankara Üniv Vet Fak Derg 55:79–82. doi:10.1501/Vetfak_0000000302.

[23] Gressley TF, Armentano LE. 2007. Effects of low rumen-degradable protein or abomasal fructan infusion on diet digestibility and urinary nitrogen excretion in lactating dairy cows. J Dairy Sci 90:1340–1353. doi:10.3168/jds.S0022-0302(07)71621-1. 17297109

[24] Giovannini AEJ, van den Borne BHP, Wall SK, Wellnitz O, Bruckmaier RM, Spadavecchia C. 2017. Experimentally induced subclinical mastitis: are lipopolysaccharide and lipoteichoic acid eliciting similar pain responses? Acta Vet Scand 59:40. doi:10.1186/s13028-017-0306-z. 28615028 PMC5471899

[25] Dong G, Liu S, Wu Y, Lei C, Zhou J, Zhang S. 2011. Diet-induced bacterial immunogens in the gastrointestinal tract of dairy cows: impacts on immunity and metabolism. Acta Vet Scand 53:48. doi:10.1186/1751-0147-53-48. 21824438 PMC3161887

[26] Li K, Zhang L, Xue J, Yang X, Dong X, Sha L, Lei H, Zhang X, Zhu L, Wang Z, Li X, Wang H, Liu P, Dong Y, He L. 2019. Dietary inulin alleviates diverse stages of type 2 diabetes mellitus via anti-inflammation and modulating gut microbiota in db/db mice. Food Funct 10:1915–1927. doi:10.1039/c8fo02265h. 30869673

[27] Zhao XH, Gong JM, Zhou S, Liu CJ, Qu MR. 2014. The effect of starch, inulin, and degradable protein on ruminal fermentation and microbial growth in rumen simulation technique. Ital J Anim Sci 13:3121. doi:10.4081/ijas.2014.3121.

[28] Umucalilar HD, Glen N, Hayirli A, Alata MS. 2010. Potential role of inulin in rumen fermentation. Revue Méd Vét 161:3–9.

[29] Smith GH, Mccarthy S, Rook JA. 1974. Synthesis of milk fat from β-hydroxybutyrate and acetate in lactating goats. J Dairy Res 41:175–191. doi:10.1017/s0022029900019609. 4837851

[30] Hall MB, Weimer PJ. 2016. Divergent utilization patterns of grass fructan, inulin, and other nonfiber carbohydrates by ruminal microbes. J Dairy Sci 99:245–257. doi:10.3168/jds.2015-10417. 26601577

[31] Li J, Sung CYJ, Lee N, Ni Y, Pihlajamäki J, Panagiotou G, El-Nezami H. 2016. Probiotics modulated gut microbiota suppresses hepatocellular carcinoma growth in mice. Proc Natl Acad Sci USA 113:E1306–1315. doi:10.1073/pnas.1518189113. 26884164 PMC4780612

[32] Macy JM, Ljungdahl LG, Gottschalk G. 1978. Pathway of succinate and propionate formation in *Bacteroides fragilis*. J Bacteriol 134:84–91. doi:10.1128/jb.134.1.84-91.1978. 148460 PMC222221

[33] Smith BJ, Miller RA, Ericsson AC, Harrison DC, Strong R, Schmidt TM. 2019. Changes in the gut microbiome and fermentation products concurrent with enhanced longevity in acarbose-treated mice. BMC Microbiol 19:130. doi:10.1186/s12866-019-1494-7. 31195972 PMC6567620

[34] Bunker JJ, Flynn TM, Koval JC, Shaw DG, Meisel M, McDonald BD, Ishizuka IE, Dent AL, Wilson PC, Jabri B, Antonopoulos DA, Bendelac A. 2015. Innate and adaptive humoral responses coat distinct commensal bacteria with immunoglobulin A. Immunity 43:541–513. doi:10.1016/j.immuni.2015.08.007. 26320660 PMC4575282

[35] Stackebrandt E, Hespell RB. 2006. The family *Succinivibrionaceae*, p 419–429. *In* Dworkin M, Falkow S, Rosenberg E, Schleifer K-H, Stackebrandt E (ed), The prokaryotes, vol 3. Springer, New York, NY. doi:10.1007/0-387-30743-5_20.

[36] Ciarlo E, Heinonen T, Herderschee J, Fenwick C, Mombelli M, Roy DL, Roger T. 2016. Impact of the microbial derived short chain fatty acid propionate on host susceptibility to bacterial and fungal infections *in vivo*. Sci Rep 6:37944. doi:10.1038/srep37944. 27897220 PMC5126587

[37] Lepage P, Häsler R, Spehlmann ME, Rehman A, Zvirbliene A, Begun A, Ott S, Kupcinskas L, Doré J, Raedler A, Schreiber S. 2011. Twin study indicates loss of interaction between microbiota and mucosa of patients with ulcerative colitis. Gastroenterology 141:227–236. doi:10.1053/j.gastro.2011.04.011. 21621540

[38] Imaoka A, Shima T, Kato K, Mizuno S, Uehara T, Matsumoto S, Setoyama H, Hara T, Umesaki Y. 2008. Anti-inflammatory activity of probiotic *Bifidobacterium*: enhancement of IL-10 production in peripheral blood mononuclear cells from ulcerative colitis patients and inhibition of IL-8 secretion in HT-29 cells. World J Gastroenterol 14:2511–2516. doi:10.3748/wjg.14.2511. 18442197 PMC2708361

[39] Belenguer A, Duncan SH, Calder AG, Holtrop G, Louis P, Lobley GE, Flint HJ. 2006. Two routes of metabolic cross-feeding between *Bifidobacterium adolescentis* and butyrate-producing anaerobes from the human gut. Appl Environ Microbiol 72:3593–3599. doi:10.1128/AEM.72.5.3593-3599.2006. 16672507 PMC1472403

[40] Couto MR, Gonçalves P, Magro F, Martel F. 2020. Microbiota-derived butyrate regulates intestinal inflammation: focus on inflammatory bowel disease. Pharmacol Res 159:104947. doi:10.1016/j.phrs.2020.104947. 32492488

[41] Bouchard DS, Seridan B, Taous S, Lucie R, Pierre G, Gonzalez-Moreno C, Nader-Macias FME, Baud D, Francois P, Chuat V, Chain F, Langella P, Nicoli J, Le Loir Y, Even S. 2015. Lactic acid bacteria isolated from bovine mammary microbiota: potential allies against bovine mastitis. PLoS One 10:e0144831. doi:10.1371/journal.pone.0144831. 26713450 PMC4694705

[42] Humphries HE, Triantafilou M, Makepeace BL, Heckels JE, Triantafilou K, Christodoulides M. 2005. Activation of human meningeal cells is modulated by lipopolysaccharide (LPS) and non-LPS components of *Neisseria* meningitidis and is independent of Toll-like receptor (TLR)4 and TLR2 signalling. Cell Microbiol 7:415–430. doi:10.1111/j.1462-5822.2004.00471.x. 15679844

[43] Tomita K, Nagura T, Okuhara Y, Adachi HN, Shigematsu N, Aritsuka T, Kaminogawa S, Hachimura S. 2007. Dietary melibiose regulates Th cell response and enhances the induction of oral tolerance. Biosci Biotechnol Biochem 71:2774–2780. doi:10.1271/bbb.70372. 17986780

[44] Wang Y, Nan X, Zhao Y, Wang H, Wang M, Jiang L, Zhang F, Xue F, Hua D, Li K, Liu J, Yao J, Xiong B. 2020. Coupling 16S rDNA sequencing and untargeted mass spectrometry for milk microbial composition and metabolites from dairy cows with clinical and subclinical mastitis. J Agric Food Chem 68:8496–8508. doi:10.1021/acs.jafc.0c03738. 32633125

[45] Davila AM, Blachier F, Gotteland M, Andriamihaja M, Benetti PH, Sanz Y, Tome D. 2013. Re-print of “intestinal luminal nitrogen metabolism: role of the gut microbiota and consequences for the host”. Pharmacol Res 69:114–126. doi:10.1016/j.phrs.2013.01.003. 23318949

[46] Drabińska N, Krupa-Kozak U, Ciska E, Jarocka-Cyrta E. 2018. Plasma profile and urine excretion of amino acids in children with celiac disease on gluten-free diet after oligofructose-enriched inulin intervention: results of a randomised placebo-controlled pilot study. Amino Acids 50:1451–1460. doi:10.1007/s00726-018-2622-7. 30043079 PMC6153951

[47] Knarreborg A, Miquel N, Granli T, Jensen BB. 2002. Establishment and application of an *in vitro* methodology to study the effects of organic acids on coliform and lactic acid bacteria in the proximal part of the gastrointestinal tract of piglets. Anim Feed Sci Technol 99:131–140. doi:10.1016/S0377-8401(02)00069-X.

[48] Hansen ME. 2014. PhD dissertation. The role of ceramides in mediating endotoxin-induced mitochondrial disruption. Brigham Young University, Provo, UT.

[49] Pezeshki A, Stordeur P, Wallemacq H, Schynts F, Stevens M, Boutet P, Peelman LJ, Spiegeleer BD, Duchateau L, Bureau F, Burvenich C. 2011. Variation of inflammatory dynamics and mediators in primiparous cows after intramammary challenge with *Escherichia coli*. Vet Res 42:15. doi:10.1186/1297-9716-42-15. 21314974 PMC3037895

[50] Boutet P, Bureau F, Degand G, Lekeux P. 2003. Imbalance between lipoxin A4 and leukotriene B4 in chronic mastitis-affected cows. J Dairy Sci 86:3430–3439. doi:10.3168/jds.S0022-0302(03)73947-2. 14672172

[51] Olofsson KE, Andersson L, Nilsson J, Björkbacka H. 2008. Nanomolar concentrations of lysophosphatidylcholine recruit monocytes and induce pro-inflammatory cytokine production in macrophages. Biochem Biophys Res Commun 370:348–352. doi:10.1016/j.bbrc.2008.03.087. 18371300

[52] Nording ML, Yang J, Hegedus CM, Bhushan A, Hammock BD. 2009. Endogenous levels of five fatty acid metabolites in exhaled breath condensate to monitor asthma by high-performance liquid chromatography: electrospray tandem mass spectrometry. IEEE Sens J 10:123–130. doi:10.1109/JSEN.2009.2035736. PMC2981865 21103452

[53] Karyak OG, Safi S, Froushani AR, Bolourchi M. 2011. Study of the relationship between oxidative stress and subclinical mastitis in dairy cattle. Iran J Vet Res 12:350–353. doi:10.1016/S0009-9120(98)00065-4.

[54] Stoyanova S, Geuns J, Hideg É, Ende WVD. 2011. The food additives inulin and stevioside counteract oxidative stress. Int J Food Sci Nutr 62:207–214. 21043580 10.3109/09637486.2010.523416

[55] Kaşikçi G, Çetın Ö, y0Bıngöl EB, Gündüz MC. 2012. Relations between electrical conductivity, somatic cell count, California mastitis test and some quality parameters in the diagnosis of subclinical mastitis in dairy cows. Turk J Vet Anim Sci 36:49–55. doi:10.3906/vet-1103-4.

[56] Wang B, Mao S, Yang H, Wu Y, Wang J, Li S, Shen Z, Liu J. 2014. Effects of alfalfa and cereal straw as a forage source on nutrient digestibility and lactation performance in lactating dairy cows. J Dairy Sci 97:7706–7715. doi:10.3168/jds.2014-7961. 25262188

[57] Caporaso JG, Lauber CL, Walters WA, Berg-Lyons D, Huntley J, Fierer N, Owens SM, Betley J, Fraser L, Bauer M, Gormley N, Gilbert JA, Smith G, Knight R. 2012. Ultra-high-throughput microbial community analysis on the Illumina Hiseq and Miseq platforms. ISME J 6:1621–1624. doi:10.1038/ismej.2012.8. 22402401 PMC3400413

[58] Caporaso JG, Kuczynski J, Stombaugh J, Bittinger K, Bushman FD, Costello EK, Fierer N, Peña AG, Goodrich JK, Gordon JI, Huttley GA, Kelley ST, Knights D, Koenig JE, Ley RE, Lozupone CA, McDonald D, Muegge BD, Pirrung M, Reeder J, Sevinsky JR, Turnbaugh PJ, Walters WA, Widmann J, Yatsunenko T, Zaneveld J, Knight R. 2010. Qiime allows analysis of high-throughput community sequencing data. Nat Methods 7:335–336. doi:10.1038/nmeth.f.303. 20383131 PMC3156573

[59] Liu C, Wu H, Liu S, Chai S, Meng Q, Zhou Z. 2019. Dynamic alterations in yak rumen bacteria community and metabolome characteristics in response to feed type. Front Microbiol 10:1116. doi:10.3389/fmicb.2019.01116. 31191470 PMC6538947

[60] Subcommittee on Dairy Cattle Nutrition, Committee on Animal Nutrition, National Research Council. 2001. Nutrient requirements of dairy cattle, 7th ed. National Academies Press, Washington DC.

