# Supplementary material for: Dietary Supplementation of Inulin Ameliorates Subclinical Mastitis via Regulation of Rumen Microbial Community and Metabolites in Dairy Cows
Source: Microbiol Spectr. 2021 Sep 8;9(2):e00105-21. doi: 10.1128/Spectrum.00105-21 (PMC8557905; doi:10.1128/Spectrum.00105-21)
Supplement: SUPPLEMENTAL FILE 1 — Supplemental material. Download SPECTRUM00105-21_Supp_1_seq5.pdf, PDF file, 4.8 MB [file spectrum00105-21_supp_1_seq5.pdf]

Table S1 Ingredients and chemical composition of basal diet (% DM)

Ingredients	%	Chemical composition	%
Corn silage	44.21	DM, % of fresh	48.0
Alfalfa hey	4.03	CP	16.5
Oat grass	2.30	NDF	30.0
Alfalfa silage	2.88	ADF	17.6
Steam-flaked corn	4.23	EE [4]	5.30
Corn	8.99	Ca	0.21
Corn husk of spray	0.58	P	0.11
Extruded soybean	1.39	Ash	7.90
Beet pellets	2.56	NEL, Mcal/kg DM	1.74
Brewer's grains	11.52		
DDGS	0.58		
Cottonseed	3.30		
Corn bran	3.38		
Soya meal	4.29		
Double-low rapeseed meal	2.88		
Megalac	0.66		
Fatty powder	0.30		
5% Premix	1.92		

DDGS, distillers dried grains with solubles.
Megalac, a rumen protected fatty acid calcium (VOLAC International Ltd., UK).
5% Premix, including (per kg of DM) 350 KIU of vitamin A, 150 KIU of vitamin D_3, 680 IU of vitamin E, 700 mg of Cu, 3,000 mg of Zn, 50,000 mg of Fe, 24,000 mg of Mn, 10 mg of Se, 100 mg of I, 20 mg of Co, ≥ 14.5 % of Ca and ≥ 2.5 % of P.
EE = Ether extract.

Table S2 Effects of inulin addition on α-abundance of ruminal microbiota

Items	Groups (n = 6)					SEM	*P*-value
	Con	I-1	I-1	I-3	I-4		
Sobs	1168^{c}	1245^{b}	1180^{c}	1303^{a}	1217^{b}	24.0	0.041
Chao	1480^{b}	1452^{b}	1562^{ab}	1630^{a}	1506^{ab}	19.2	0.035
Ace	1444	1457	1514	1584	1491	25.5	0.293
Shannon	4.69^{b}	4.79^{b}	5.15^{ab}	5.54^{a}	4.94^{b}	0.087	0.037
Simpson	0.010^{c}	0.012^{c}	0.012^{c}	0.018^{a}	0.015^{b}	0.0012	0.021
Good's coverage	0.99	0.99	0.99	0.99	0.99	0.0004	0.211

Con = control group; I-1 = inulin-1 group, the inulin addition level was 100 g/d per cow; I-2 = inulin-2 group, the inulin addition level was 200 g/d per cow; I-3 = inulin-3 group, the inulin addition level was 300 g/d per cow; I-4 = inulin-4 group, the inulin addition level was 400 g/d per cow. $^{a, b, c}$ = within a row, different letters differed significantly ($P < 0.05$).

Table S3 Effects of inulin on the relative abundances of ruminal microbiota at phyla level.

NO.	Items	Con	I-1	I-2	I-3	I-4	SEM	*P*-value	Corrected *P*-value
1	*Bacteroidota*	46.6^{c}	46.7^{c}	52.2^{b}	56.4^{a}	52.6^{b}	1.65	0.004	0.033
2	*Firmicutes*	42.5^{a}	42.9^{a}	36.0^{b}	31.3^{c}	35.4^{b}	1.47	0.009	0.037
3	*Proteobacteria*	6.38^{b}	6.23^{b}	7.17^{a}	7.39^{a}	7.09^{a}	0.937	0.018	0.048
4	*Patescibacteria*	2.08	2.06	1.86	1.92	2.07	0.104	0.035	0.086
5	*Actinobacteriota*	1.10^{c}	1.06^{c}	1.62^{b}	1.85^{a}	1.54^{b}	0.07	0.006	0.037
6	*Spirochaetota*	0.45	0.48	0.40	0.49	0.57	0.038	0.032	0.082
7	*Cyanobacteria*	0.30	0.29	0.27	0.28	0.27	0.038	0.027	0.082
8	*Desulfobacterota*	0.18	0.10	0.12	0.11	0.13	0.01	0.092	0.185
9	*Bacteria*	0.17	0.12	0.13	0.11	0.14	0.014	0.356	0.458
10	*Verrucomicrobiota*	0.08	0.07	0.12	0.05	0.08	0.01	0.413	0.465
11	*Synergistota*	0.06	0.04	0.04	0.02	0.02	0.005	0.219	0.329
12	*Fibrobacterota*	0.04	0.04	0.03	0.05	0.03	0.004	0.413	0.465
13	*Elusimicrobiota*	0.024	0.016	0.021	0.018	0.024	0.0026	0.076	0.170
14	*WPS-2*	0.015	0.012	0.008	0.014	0.015	0.0021	0.548	0.548
15	*Campilobacterota*	0.012^{a}	0.007^{b}	0.006^{b}	0.007^{b}	0.009^{ab}	0.0012	0.025	0.117
16	*Chloroflexi*	0.006^{b}	0.006^{b}	0.012^{a}	0.001^{c}	0.001^{c}	0.0012	0.011	0.098
17	*Armatimonadota*	0.001	0.000	0.001	0.000	0.001	0.0003	0.446	0.472
18	*Fusobacteriota*	0.000	0.002	0.001	0.000	0.001	0.0004	0.158	0.284

Con = control group; I-1 = inulin-1 group, the inulin addition level was 100 g/d per cow; I-2 = inulin-2 group, the inulin addition level was 200 g/d per cow; I-3 = inulin-3 group, the inulin addition level was 300 g/d per cow; I-4 = inulin-4 group, the inulin addition level was 400 g/d per cow; FDR = false discovery rate; $^{a, b, c}$ = within a row, different letters differed significantly (FDR-

adjusted $P < 0.05$).

Table S4 Effects of inulin on the relative abundances of ruminal microbiota at genus level.

NO.	Items	Con	I-1	I-2	I-3	I-4	SEM	*P*-value	FDR
1	*Prevotella*	14.2^{c}	16.7^{c}	19.3^{b}	22.4^{a}	20.4^{b}	1.30	<0.001	0.013
2	*Ruminococcaceae*	8.24^{a}	8.52^{a}	6.78^{b}	6.13^{b}	6.60^{b}	0.214	0.004	0.035
3	*Succiniclasticum*	4.69	4.92	3.08	3.53	3.73	0.335	0.065	0.216
4	*Succinivibrionaceae_UCG-001*	3.89^{b}	4.12^{b}	4.93^{ab}	5.44^{a}	5.21^{a}	0.270	0.007	0.038
5	*Muribaculaceae*	3.55^{c}	3.67^{c}	3.80^{c}	5.04^{a}	4.20^{b}	0.126	0.005	0.033
6	*Oscillospira*	3.25^{a}	2.95^{b}	2.61^{b}	1.07^{c}	1.46^{c}	0.067	0.011	0.042
7	*Clostridia_UCG-014*	2.97^{a}	2.69^{a}	2.26^{a}	1.58^{b}	1.65^{b}	0.078	0.008	0.035
8	*Coprococcus*	2.89^{a}	2.54^{a}	1.54^{b}	1.18^{c}	1.30^{bc}	0.124	0.004	0.036
9	*Butyrivibrio*	2.70^{c}	3.11^{b}	4.35^{a}	4.71^{a}	3.89^{b}	0.197	0.002	0.031
10	*Christensenellaceae_R-7_group*	2.69^{c}	2.77^{c}	3.84^{b}	4.29^{a}	3.75^{b}	0.182	0.004	0.038
11	*Ruminococcaceae_NK4A214_group*	2.63	2.74	2.64	3.07	3.04	0.273	0.075	0.232
12	*Bacteroidales_BS11_gut_group*	2.33^{b}	2.40^{b}	3.81^{a}	3.46^{a}	3.65^{a}	0.234	0.011	0.048
13	*Rikenellaceae_RC9_gut_group*	2.22^{c}	2.47^{c}	3.72^{b}	4.23^{a}	4.04^{a}	0.168	0.006	0.039
14	*Ruminococcus*	2.14^{a}	2.76^{a}	2.30^{a}	0.77^{b}	0.83^{b}	0.241	0.012	0.043
15	*F082*	1.74	1.39	0.75	0.56	0.73	0.093	0.394	0.547
16	*Prevotellaceae_UCG-001*	1.58	1.45	1.47	1.02	1.29	0.071	0.109	0.271
17	*Eubacterium_ruminantium_group*	1.47^{c}	1.32^{c}	2.53^{b}	2.46^{b}	3.16^{a}	0.137	0.002	0.031
18	*Bacteroidales_RF16_group*	1.42^{b}	1.37^{b}	2.22^{a}	2.60^{a}	2.35^{a}	0.112	0.017	0.045
19	*Selenomonas*	1.38	1.55	1.40	1.22	1.71	0.121	0.784	0.806
20	*Lachnospiraceae_NK3A20_group*	1.33^{b}	1.23^{b}	1.56^{ab}	1.89^{a}	1.80^{a}	0.106	0.015	0.046
21	*Shuttleworthia*	1.31	0.73	0.86	0.95	0.18	0.144	0.136	0.305
22	*Lactobacillus*	1.23^{c}	1.40^{c}	2.36^{a}	2.35^{a}	1.98^{b}	0.111	0.013	0.042
23	*Ruminococcus_gauvreauii_group*	1.19	1.07	1.07	0.79	1.24	0.069	0.267	0.440
24	*Prevotellaceae_UCG-003*	1.11	1.17	1.03	1.18	1.36	0.072	0.711	0.763
25	*Absconditabacteriales_SR1*	1.08	0.98	0.99	0.94	0.47	0.079	0.101	0.264
26	*Prevotellaceae*	1.06	0.73	0.47	0.70	0.90	0.090	0.093	0.258
27	*Acetitomaculum*	1.01^{a}	0.67^{b}	0.52^{b}	0.92^{b}	1.29^{a}	0.088	0.016	0.044
28	*Bifidobacterium*	0.96^{c}	0.97^{c}	1.41^{b}	1.65^{a}	1.25^{bc}	0.080	0.013	0.043
29	*Streptococcus*	0.94^{a}	0.73^{a}	0.43^{b}	0.39^{b}	0.41^{b}	0.022	0.017	0.048
30	*Lachnospiraceae*	0.93	0.50	0.50	0.61	0.47	0.043	0.362	0.515
31	*Candidatus_Saccharimonas*	0.90	0.87	0.53	0.50	0.89	0.052	0.103	0.265
32	*RF39*	0.88	0.77	0.51	0.58	0.68	0.046	0.080	0.242
33	*Eubacterium_coprostanoligenes_group*	0.85	0.89	0.80	0.50	0.84	0.059	0.881	0.896
34	*Pseudobutyrivibrio*	0.80	0.64	0.55	0.52	0.45	0.049	0.192	0.367
35	*Prevotellaceae*	0.74	0.59	0.58	0.23	0.34	0.050	0.023	0.052
36	*Treponema*	0.74	0.47	0.49	0.39	0.55	0.037	0.031	0.057
37	*Schwartzia*	0.71	0.59	0.28	0.30	0.54	0.051	0.032	0.077
38	*UCG-011*	0.70	0.41	0.53	0.39	0.69	0.049	0.259	0.420
39	*Selenomonadaceae*	0.70	0.37	0.34	0.38	0.11	0.071	0.109	0.271
40	*Saccharofermentans*	0.62	0.61	0.44	0.37	0.53	0.027	0.181	0.345
41	*Selenomonadaceae*	0.62	0.57	0.32	0.39	0.05	0.087	0.311	0.477
42	*Lachnospiraceae_NK4A136_group*	0.61	0.51	0.23	0.27	0.11	0.040	0.073	0.132
43	*Neisseriaceae*	0.60^{a}	0.48^{a}	0.19^{b}	0.11^{b}	0.12^{b}	0.012	0.015	0.046
44	*Succinivibrionaceae_UCG-002*	0.55	0.52	0.46	0.07	0.23	0.080	0.316	0.479
45	*Syntrophococcus*	0.53	0.54	0.47	0.39	0.11	0.077	0.427	0.564
46	*Escherichia-Shigella*	0.64^{a}	0.53^{a}	0.25^{b}	0.16^{c}	0.21^{b}	0.041	0.014	0.045

47	*Lachnospira*	0.43	0.48	0.34	0.24	0.16	0.035	0.124	0.281
48	*Bacteroidales*	0.40	0.42	0.20	0.18	0.19	0.023	0.044	0.089
49	*Gastranaerophilales*	0.37	0.46	0.17	0.15	0.06	0.037	0.097	0.164
50	*Olsenella*	0.35	0.45	0.24	0.20	0.25	0.038	0.815	0.849
51	*UCG-005*	0.34	0.42	0.11	0.18	0.55	0.040	0.271	0.440
52	*Oribacterium*	0.33	0.49	0.37	0.14	0.27	0.028	0.114	0.273
53	*Eubacterium_hallii_group*	0.32	0.47	0.27	0.13	0.28	0.014	0.480	0.630
54	*Prevotellaceae_UCG-004*	0.31	0.40	0.20	0.16	0.21	0.019	0.116	0.273
55	*Prevotellaceae_Ga6A1_group*	0.30	0.28	0.36	0.12	0.07	0.035	0.064	0.166
56	*Family_XIII_AD3011_group*	0.30	0.22	0.18	0.11	0.31	0.020	0.099	0.174
57	*Lachnospiraceae_AC2044_group*	0.29	0.15	0.20	0.32	0.15	0.023	0.036	0.078
58	*Mogibacterium*	0.27	0.17	0.17	0.21	0.25	0.020	0.362	0.515
59	*Lachnospiraceae*	0.26	0.16	0.22	0.08	0.20	0.026	0.255	0.420
60	*UCG-001*	0.25	0.46	0.36	0.18	0.16	0.040	0.092	0.161
61	*Nocardioides*	0.24	0.17	0.14	0.08	0.17	0.015	0.025	0.063
62	*Eubacterium_nodatum_group*	0.24	0.19	0.18	0.10	0.26	0.013	0.213	0.381
63	*p-251-o5*	0.22	0.10	0.15	0.19	0.26	0.029	0.473	0.618
64	*FD2005*	0.11	0.18	0.12	0.06	0.04	0.015	0.062	0.126
65	*Anaerovibrio*	0.21	0.20	0.26	0.45	0.45	0.042	0.208	0.380
66	*Colidextribacter*	0.21	0.55	0.45	0.22	0.38	0.041	0.022	0.063
67	*probable_genus_10*	0.20	0.08	0.07	0.12	0.10	0.012	0.044	0.199
68	*Lachnobacterium*	0.19	0.12	0.09	0.06	0.05	0.014	0.023	0.058
69	*Veillonellaceae_UCG-001*	0.18	0.11	0.05	0.14	0.12	0.017	0.231	0.389
70	*Anaeroplasma*	0.18	0.32	0.21	0.12	0.34	0.024	0.186	0.368
71	*Lachnospiraceae_XPB1014_group*	0.18	0.08	0.08	0.14	0.12	0.012	0.026	0.059
72	*Moryella*	0.17	0.11	0.11	0.06	0.13	0.012	0.022	0.057
73	*Eubacterium_ventriosum_group*	0.17	0.16	0.06	0.04	0.02	0.013	0.061	0.162
74	*Staphylococcus*	0.17[a]	0.12[b]	0.11[b]	0.09[c]	0.07[c]	0.014	0.014	0.045
75	*UCG-002*	0.15	0.20	0.13	0.06	0.20	0.017	0.093	0.162
76	*Lachnospiraceae_UCG-008*	0.15	0.16	0.10	0.05	0.13	0.012	0.072	0.173
77	*UCG-004*	0.14	0.25	0.25	0.28	0.31	0.022	0.146	0.205
78	*Eubacterium_cellulosolvens_group*	0.14	0.08	0.11	0.10	0.08	0.008	0.283	0.360
79	*UCG-010*	0.13	0.13	0.15	0.20	0.18	0.017	0.578	0.687
80	*Bacteroidales*	0.13	0.16	0.05	0.06	0.03	0.011	0.032	0.074
81	*Oscillospiraceae*	0.12	0.12	0.10	0.12	0.11	0.009	0.871	0.896
82	*Marvinbryantia*	0.12	0.11	0.14	0.07	0.18	0.015	0.602	0.699
83	*Succinivibrio*	0.02	0.04	0.02	0.05	0.04	0.009	0.104	0.231
84	*Bacteroidales_UCG-001*	0.11	0.06	0.08	0.01	0.18	0.009	0.302	0.472
85	*Pseudoscardovia*	0.11	0.06	0.05	0.04	0.00	0.011	0.094	0.163
86	*Anaerovoracaceae*	0.10	0.09	0.07	0.07	0.09	0.007	0.392	0.447
87	*Desulfovibrio*	0.10	0.06	0.05	0.05	0.07	0.006	0.121	0.281
88	*CAG-352*	0.09	0.07	0.40	0.06	0.04	0.063	0.320	0.481
89	*Roseburia*	0.08	0.07	0.04	0.09	0.05	0.008	0.166	0.221
90	*Acetobacter*	0.08[b]	0.14[a]	0.14[a]	0.17[a]	0.17[a]	0.005	0.017	0.048
91	*Lachnospiraceae_UCG-008*	0.08	0.06	0.05	0.04	0.08	0.004	0.104	0.264
92	*Lachnospiraceae_ND3007_group*	0.08	0.09	0.11	0.11	0.15	0.013	0.549	0.668
93	*WCHB1-41*	0.08	0.07	0.05	0.11	0.08	0.010	0.387	0.428
94	*Howardella*	0.07	0.06	0.05	0.06	0.07	0.005	0.381	0.428
95	*Dialister*	0.07	0.06	0.01	0.07	0.07	0.009	0.023	0.057
96	*Ruminococcaceae*	0.06	0.09	0.10	0.09	0.12	0.012	0.729	0.772

97	*Alloprevotella*	0.06	0.05	0.06	0.04	0.03	0.005	0.087	0.152
98	*Anaerovorax*	0.06	0.05	0.06	0.05	0.07	0.005	0.692	0.735
99	*DNF00809*	0.06	0.05	0.03	0.08	0.09	0.008	0.102	0.264
100	*Defluviitaleaceae_UCG-011*	0.06	0.05	0.04	0.05	0.08	0.006	0.142	0.205
101	*p-2534-18B5_gut_group*	0.06	0.08	0.01	0.05	0.05	0.058	0.120	0.281
102	*Eubacterium_brachy_group*	0.05	0.03	0.03	0.04	0.06	0.004	0.198	0.268
103	*Lachnospiraceae_UCG-002*	0.05	0.03	0.03	0.03	0.04	0.003	0.124	0.293
104	*Blautia*	0.05	0.05	0.04	0.08	0.13	0.009	0.033	0.078
105	*Papillibacter*	0.05	0.03	0.05	0.06	0.04	0.005	0.634	0.715
106	*Bradymonadales*	0.05	0.02	0.03	0.02	0.03	0.005	0.192	0.268
107	*Atopobium*	0.05	0.05	0.02	0.04	0.07	0.005	0.069	0.116
108	*U29-B03*	0.04	0.08	0.07	0.06	0.09	0.006	0.084	0.132
109	*Erysipelotrichaceae_UCG-002*	0.04	0.04	0.28	0.18	0.00	0.057	0.112	0.271
110	*Fibrobacter*	0.04	0.04	0.05	0.02	0.03	0.004	0.464	0.508
111	*Lachnospiraceae_FE2018_group*	0.04	0.03	0.02	0.02	0.04	0.003	0.496	0.539
112	*Pyramidobacter*	0.04	0.02	0.00	0.01	0.02	0.003	0.036	0.083
113	*Rhodospirillales*	0.03	0.03	0.01	0.01	0.01	0.003	0.380	0.427
114	*Family_XIII_UCG-001*	0.03	0.04	0.02	0.02	0.02	0.002	0.385	0.428
115	*Bacillus*	0.03	0.03	0.02	0.01	0.01	0.002	0.472	0.518
116	*Erysipelotrichaceae*	0.03	0.03	0.03	0.02	0.02	0.003	0.147	0.210
117	*Tyzzerella*	0.03	0.06	0.04	0.01	0.04	0.005	0.315	0.479
118	*Clostridia*	0.03	0.02	0.03	0.00	0.01	0.003	0.879	0.896
119	*Eggerthellaceae*	0.03	0.02	0.03	0.01	0.02	0.004	0.207	0.380
120	*Others*	0.27^{b}	0.34^{a}	0.28^{b}	0.18^{c}	0.25^{b}	0.023	0.011	0.046

Con = control group; I-1 = inulin-1 group, the inulin addition level was 100 g/d per cow; I-2 = inulin-2 group, the inulin addition level was 200 g/d per cow; I-3 = inulin-3 group, the inulin addition level was 300 g/d per cow; I-4 = inulin-4 group, the inulin addition level was 400 g/d per cow; FDR = false discovery rate; $^{a, b, c}$ = within a row, different letters differed significantly (FDR-adjusted $P < 0.05$).

Table S5 Correlation analysis between significantly different ruminal microbiota and rumen fermentation paraments as well as milk compositions

Microbiota	Items	R	FDR
Acetobacter	Acetate	0.625	0.028
Rikenellaceae_RC9_gut_group	Acetate	0.429	0.018
Christensenellaceae_R-7_group	Acetate	0.547	0.044
Muribaculaceae	Propionate	0.546	0.021
Prevotella	Propionate	0.611	0.000
Bacteroidales_RF16_group	Propionate	0.673	0.000
Bacteroidales_BS11_gut_group	Propionate	0.551	0.002
Bifidobacterium	Butyrate	0.541	0.040
Lachnospiraceae_NK3A20_group	Butyrate	0.574	0.021
Butyrivibrio	Butyrate	0.533	0.022
Eubacterium_ruminantium_group	Butyrate	0.525	0.023
Christensenellaceae_R-7_group	Butyrate	0.341	0.065
Bifidobacterium	LA	0.603	0.042
Lachnospiraceae_NK3A20_group	LA	0.654	0.009
Lactobacillus	LA	0.577	0.001
Streptococcus	LA	-0.621	0.025
Staphylococcus	LA	-0.561	0.033
Escherichia-Shigella	LA	-0.683	0.032
Muribaculaceae	Milk lactose	0.297	0.111
Rikenellaceae_RC9_gut_group	Milk lactose	0.549	0.040

Bacteroidales_RF16_group	Milk lactose	0.145	0.443
Bacteroidales_BS11_gut_group	Milk lactose	0.483	0.007
Clostridia_UCG-014	Milk lactose	-0.521	0.003
Staphylococcus	Milk lactose	-0.450	0.012
Muribaculaceae	Milk SCC	-0.575	0.041
Butyrivibrio	Milk SCC	-0.563	0.001
Lactobacillus	Milk SCC	-0.607	0.037
Clostridia_UCG-014	Milk SCC	0.597	0.049
Streptococcus	Milk SCC	0.621	0.025
Staphylococcus	Milk SCC	0.447	0.013
Ruminococcaceae	Milk SCC	0.462	0.010
Neisseriaceae	Milk SCC	0.630	0.000
Escherichia-Shigella	Milk SCC	0.463	0.040

LA = lactic acid; SCC = somatic cell counts; R = Correlation coefficient; FDR = false discovery rate

Table S6 The significantly differential rumen metabolites (Top 50 of VIP value) between control and inulin-1 group

Metabolite	M/Z	Mode	Formula	Retention time	HMDB Superclass	HMDB Class	HMDB Subclass	VIP	FC (I-1/Con)	*P*-value	FDR
Melibiose	377.09	neg	$C_{12}H_{22}O_{11}$	0.61	Organic oxygen compounds	Organooxygen compounds	Carbohydrates and carbohydrate conjugates	4.113	3.183	0.003	0.026
N-Acetyllactosamine	348.13	pos	$C_{14}H_{25}NO_{11}$	2.55	Organic oxygen compounds	Organooxygen compounds	Carbohydrates and carbohydrate conjugates	4.067	2.682	0.002	0.024
D-Fructose	179.06	neg	$C_6H_{12}O_6$	0.61	Organic oxygen compounds	Organooxygen compounds	Carbohydrates and carbohydrate conjugates	3.805	2.116	4.44×10^{-5}	0.003
N-acetyl-S-(3-oxo-3-carboxy-n-propyl) cysteine	305.08	pos	$C_9H_{13}NO_6S$	2.39	Organic acids and derivatives	Carboxylic acids and derivatives	Amino acids, peptides, and analogues	3.513	3.183	0.004	0.032
Cer (d18:0/13:0)	403.18	neg	$C_{21}H_{26}O_5$	5.09	Organic oxygen compounds	Organooxygen compounds	Carbonyl compounds	3.473	0.879	0.004	0.037
Cer (d18:0/15:0)	213.16	pos	$C_{11}H_{22}N_2O_3$	4.66	Organic acids and derivatives	Carboxylic acids and derivatives	Amino acids, peptides, and analogues	3.389	0.890	0.001	0.034
LysoPC (18:0)	185.13	pos	$C_9H_{18}N_2O_3$	2.98	Organic acids and derivatives	Carboxylic acids and derivatives	Amino acids, peptides, and analogues	3.157	0.687	0.002	0.048
Isoleucyl-Alanine	383.21	pos	$C_{20}H_{30}O_7$	5.97	Lipids and lipid-like molecules	Fatty Acyls	Eicosanoids	3.089	0.714	0.003	0.040
12-Oxo-20-trihydroxy-leukotriene B4	327.07	neg	$C_{19}H_{14}O_4$	0.50	Benzenoids	Naphthalenes	Phenylnaphthalenes	3.038	0.612	0.010	0.058
PC (15:0/20:2(11Z,14Z))	151.08	neg	$C_9H_{14}O_3$	4.16	Organic acids and derivatives	Keto acids and derivatives	Alpha-keto acids and derivatives	2.974	1.312	0.002	0.028
4-Pentenyl acetate	149.06	neg	$C_7H_{12}O_2$	4.16	Organic acids and derivatives	Carboxylic acids and derivatives	Carboxylic acid derivatives	2.974	1.312	0.002	0.027
Phenylalanyl-Lysine	746.57	pos	$C_{41}H_{80}NO_8P$	7.56	Lipids and lipid-like molecules	Glycerophospholipids	Glycerophosphoethanola mines	2.691	0.798	0.012	0.058
Valyl-Isoleucine	794.57	pos	$C_{43}H_{82}NO_8P$	7.53	Lipids and lipid-like molecules	Glycerophospholipids	Glycerophosphocholines	2.660	0.642	0.018	0.043
PC (16:0/18:1(11Z))	782.57	pos	$C_{42}H_{82}NO_8P$	7.73	Lipids and lipid-like molecules	Glycerophospholipids	Glycerophosphocholines	2.582	1.146	0.008	0.048
8-Hydroxy-2-methoxy-6-methyl-1,4-naphthoquinone	263.06	neg	$C_{12}H_{10}O_4$	0.62	Benzenoids	Naphthalenes	Naphthoquinones	2.582	1.146	0.001	0.018
(±)-Enterolactone	297.11	neg	$C_{18}H_{18}O_4$	5.90	-	-	-	2.544	1.268	0.001	0.033
LysoPE (0:0/20:3(11Z,14Z,17Z))	548.30	neg	$C_{25}H_{46}NO_7P$	6.74	Lipids and lipid-like molecules	Glycerophospholipids	Glycerophosphoethanola mines	2.544	1.268	0.006	0.046
Isoleucyl-Glutamine	242.15	pos	$C_{11}H_{21}N_3O_4$	2.35	Organic acids and derivatives	Carboxylic acids and derivatives	Amino acids, peptides, and analogues	2.544	1.268	0.020	0.049
Benzoic acid	315.07	neg	$C_{13}H_{16}O_9$	1.69	Benzenoids	Benzene and substituted derivatives	Benzoic acids and derivatives	2.457	1.209	0.002	0.025
Zeranol	343.15	neg	$C_{18}H_{26}O_5$	5.09	Phenylpropanoids and polyketides	Macrolides and analogues	Not Available	2.457	1.209	0.011	0.042

L-Glutamate	146.04	neg	$C_5H_9NO_4$	0.61	Organoheterocyclic compounds	Indoles and derivatives	Indolyl carboxylic acids and derivatives	2.430	1.142	3.14×10^{-6}	0.001
9,12,13-TriHOME	338.17	neg	$C_{15}H_{23}N_3O_3$	3.45	Organic acids and derivatives	Carboxylic acids and derivatives	Amino acids, peptides, and analogues	2.410	0.835	0.002	0.022
Tyrosyl-Isoleucine	472.41	pos	$C_{29}H_{50}O_2$	5.90	Lipids and lipid-like molecules	Prenol lipids	Triterpenoids	2.408	0.895	0.001	0.019
1-(2-Furyl) butan-3-one	137.06	neg	$C_8H_{10}O_2$	2.54	Organoheterocyclic compounds	Heteroaromatic compounds	Not Available	2.382	1.098	0.016	0.055
L-Tyrosine	146.06	pos	$C_9H_{11}NO_3$	2.21	Organic acids and derivatives	Carboxylic acids and derivatives	Amino acids, peptides, and analogues	2.382	1.098	0.005	0.034
PC (15:0/20:5(5Z,8Z,11Z,14Z,17Z))	798.56	pos	$C_{43}H_{76}NO_8P$	6.93	Lipids and lipid-like molecules	Glycerophospholipids	Glycerophosphocholines	2.379	1.160	0.012	0.048
PC (16:0/18:2(9Z,12Z))	758.57	pos	$C_{42}H_{80}NO_8P$	7.43	Lipids and lipid-like molecules	Glycerophospholipids	Glycerophosphocholines	2.363	1.125	0.007	0.047
PG (18:0/22:4(7Z,10Z,13Z,16Z))	865.53	pos	$C_{46}H_{83}O_{10}P$	7.44	Lipids and lipid-like molecules	Glycerophospholipids	Glycerophosphoglycerols	2.363	1.125	0.015	0.067
{[1-(2-hydroxyphenyl)-3-oxopropan-2-yl] oxy} sulfonic acid	245.01	neg	$C_9H_{10}O_6S$	2.81	Benzenoids	Phenols	1-hydroxy-4-unsubstituted benzenoids	2.357	1.208	0.015	0.043
DHAP (10:0)	325.14	pos	$C_{13}H_{25}O_7P$	3.21	Organic oxygen compounds	Organooxygen compounds	Carbonyl compounds	2.357	1.208	0.001	0.031
Phosphocholine	184.07	pos	$C_5H_{14}NO_4P$	6.42	-	-	-	2.357	1.208	0.000	0.009
17-phenyl-18,19,20-trinor-prostaglandin E2	407.19	neg	$C_{23}H_{30}O_5$	8.32	Lipids and lipid-like molecules	Fatty Acyls	Eicosanoids	2.336	0.903	2.75×10^{-6}	0.0013
{3-[(1E)-3-hydroxyprop-1-en-1-yl] phenyl} oxidenes ulfonic acid	275.02	neg	$C_9H_{10}O_5S$	1.50	Organic acids and derivatives	Organic sulfuric acids and derivatives	Arylsulfates	2.282	1.256	4.72×10^{-5}	0.003
Tetraphyllin B	322.07	neg	$C_{12}H_{17}NO_7$	1.43	Organic oxygen compounds	Organooxygen compounds	Carbohydrates and carbohydrate conjugates	2.199	1.224	0.013	0.056
PENMe2(16:1(9Z)/20:5(5Z,8Z,11Z,14Z,17Z))	796.55	pos	$C_{43}H_{74}NO_8P$	6.80	Lipids and lipid-like molecules	Glycerophospholipids	Glycerophosphoethanolamines	2.192	1.119	0.017	0.052
N-[1-(4-methoxy-6-oxopyran-2-yl)-2-methylbutyl] acetamide	234.11	neg	$C_{13}H_{19}NO_4$	5.97	-	-	-	2.191	1.160	0.007	0.048
FAHFA (18:1(9Z)/12-O-18:0)	609.49	pos	$C_{36}H_{68}O_4$	6.92	Lipids and lipid-like molecules	Fatty Acyls	Fatty acids and conjugates	2.186	1.183	0.00832	0.0304
Fumonisin B1	722.40	pos	$C_{34}H_{59}NO_{15}$	5.83	Organic acids and derivatives	Carboxylic acids and derivatives	Fumonisins	2.178	0.894	0.001	0.016
D-Vacciniin	283.08	neg	$C_{13}H_{16}O_7$	3.32	Benzenoids	Benzene and substituted derivatives	Benzoic acids and derivatives	2.178	1.116	0.001	0.019
5'-O-Methylmelledonal	464.23	pos	$C_{24}H_{30}O_8$	2.66	Lipids and lipid-like molecules	Prenol lipids	Sesquiterpenoids	2.16	1.24	0.032	0.105
Gamma-linolenyl carnitine	458.27	neg	$C_{25}H_{43}NO_4$	6.33	-	-	-	2.16	1.14	0.010	0.057

3-[1-hydroxy-2-(2-hydroxyphenyl) ethyl]-5-methoxyphenol	259.10	neg	$C_{15}H_{16}O_4$	4.19	Phenylpropanoids and polyketides	Stilbenes	Not Available	2.15	1.15	0.013	0.048
L-Fucose	163.06	neg	$C_6H_{12}O_5$	0.68	Organic oxygen compounds	Organooxygen compounds	Carbohydrates and carbohydrate conjugates	2.14	1.09	9.68×10^{-5}	0.005
2-Phenyl-1,3-dioxolane-4-methanol	405.15	neg	$C_{10}H1_2O_3$	4.75	Benzenoids	Benzene and substituted derivatives	Not Available	2.14	1.15	0.004	0.035
Kukoamine D	495.30	pos	$C_{28}H_{42}N_4O_6$	2.56	Benzenoids	Phenols	Benzenediols	2.13	1.14	0.013	0.051
2,4-Dihydroxyacetophenone 5-sulfate	231.00	neg	$C_8H_8O_6S$	2.23	Organic oxygen compounds	Organooxygen compounds	Carbonyl compounds	2.10	1.13	0.002	0.027
Ochratoxin A	402.07	neg	$C_{20}H_{18}C_lNO_6$	4.51	Phenylpropanoids and polyketides	Ochratoxins and related substances	Not Available	2.09	1.13	0.012	0.045
Retinoyl b-glucuronide	509.28	pos	$C_{26}H_{36}O_8$	1.898	Lipids and lipid-like molecules	Prenol lipids	Terpene glycosides	2.07	1.20	0.013	0.042
Conhydrinone	186.11	neg	$C_8H_{15}NO$	4.19	Organoheterocyclic compounds	Piperidines	Not Available	2.06	1.11	0.006	0.035
Alpha-Hydrojuglone 4-O-b-D-glucoside	337.09	neg	$C_{16}H_{18}O_8$	0.61	Organic oxygen compounds	Organooxygen compounds	Carbohydrates and carbohydrate conjugates	2.05	1.10	0.000	0.012

VIP = variable importance in the projection; pos = positive ion; neg = negative ion; FC = fold change; FDR = false discovery rate; I-1 = inulin-1, inulin addition level was 100 g/d per cow; - = the compound classification was unknown

Table S7 The significantly differential rumen metabolites (Top 50 of VIP value) between control and inulin-2 group

**Metabolite**	**M/Z**	**Mode**	**Formula**	**Retention time**	**HMDB Superclass**	**HMDB Class**	**HMDB Subclass**	**VIP**	**FC (I-2/Con)**	***P*-value**	**FDR**
Cer (d18:0/13:0)	498.49	pos	$C_{31}H_{63}NO_3$	7.47	-	-	-	4.32	0.27	0.005	0.026
Cer (d18:0/15:0)	275.02	neg	$C_{14}H_{12}O_2S_2$	3.45	Organoheterocyclic compounds	Bi- and oligothiophenes	Not Available	3.92	0.69	0.000	0.001
N-Acetylserotonin	199.09	neg	$C_{12}H_{14}N_2O_2$	4.26	Organoheterocyclic compounds	Indoles and derivatives	Hydroxyindoles	3.70	3.33	0.008	0.034
9,12,13-TriHOME	236.10	pos	$C_{11}H_{10}N_2O_3$	1.96	Organic acids and derivatives	Carboxylic acids and derivatives	Amino acids, peptides, and analogues	3.42	0.53	0.0021	0.030
Melibiose	377.09	neg	$C_{12}H_{22}O_{11}$	0.61	Organic oxygen compounds	Organooxygen compounds	Carbohydrates and carbohydrate conjugates	3.28	4.35	0.005	0.037
D-Fructose	179.06	neg	$C_6H_{12}O_6$	0.61	Organic oxygen compounds	Organooxygen compounds	Carbohydrates and carbohydrate conjugates	3.19	3.28	0.000	0.005
Valyl-Isoleucine	657.20	neg	$C_{28}H_{36}O_{15}$	5.74	Phenylpropanoids and polyketides	Flavonoids	Flavonoid glycosides	2.91	0.68	0.013	0.047
17-phenyl-18,19,20-trinor-prostaglandin E2	407.19	neg	$C_{23}H_{30}O_5$	8.32	Lipids and lipid-like molecules	Fatty Acyls	Eicosanoids	2.88	0.81	0.005	0.023
LysoPC (18:0)	375.24	neg	$C_{18}H_{34}O_5$	6.39	Lipids and lipid-like molecules	Fatty Acyls	Fatty acids and conjugates	2.79	0.77	0.003	0.016
Medicarpin 3-O-(6'-malon	539.12	neg	$C_{25}H_{26}O_{12}$	5.22	Phenylpropanoids	Isoflavonoids	Furanoisoflavonoids	2.74	1.33	0.004	0.021

ylglucoside)					and polyketides						
Demethyloleuropein	507.15	neg	$C_{24}H_{30}O_{13}$	4.34	Phenylpropanoids and polyketides	Flavonoids	Flavonoid glycosides	2.72	0.56	0.014	0.052
Lycoperdic acid	198.04	neg	$C_8H_{11}NO_6$	2.24	Organic acids and derivatives	Carboxylic acids and derivatives	Amino acids, peptides, and analogues	2.72	1.40	0.002	0.013
Phenylalanyl-Lysine	338.17	neg	$C_{15}H_{23}N_3O_3$	3.45	Organic acids and derivatives	Carboxylic acids and derivatives	Amino acids, peptides, and analogues	2.70	0.70	0.003	0.017
Oxypinnatanine	295.07	neg	$C_{10}H_{16}N_2O_6$	6.18	Organic acids and derivatives	Carboxylic acids and derivatives	Amino acids, peptides, and analogues	2.70	0.71	0.013	0.047
PC (16:0/18:1(11Z))	782.57	pos	$C_{42}H_{82}NO_8P$	7.73	Lipids and lipid-like molecules	Glycerophospholipids	Glycerophosphocholines	2.70	2.16	0.005	0.038
Harmaline	195.09	neg	$C_{13}H_{14}N_2O$	5.53	Alkaloids and derivatives	Harmala alkaloids	Not Available	2.65	1.27	0.000	0.002
2'-Hydroxyenterolactone	332.15	pos	$C_{18}H_{18}O_5$	2.67	Lignans, neolignans and related compounds	Furanoid lignans	Tetrahydrofuran lignans	2.64	1.18	2.197×10^{-6}	0.001
4-Pentenyl acetate	149.06	neg	$C_7H_{12}O_2$	4.16	Organic acids and derivatives	Carboxylic acids and derivatives	Carboxylic acid derivatives	2.62	1.20	0.000	0.004
FAHFA (18:1(9Z)/12-O-18:0)	609.49	pos	$C_{36}H_{68}O_4$	6.92	Lipids and lipid-like molecules	Fatty Acyls	Fatty acids and conjugates	2.58	1.25	0.012	0.044
L-Glutamate	146.04	neg	$C_5H_9NO_4$	0.61	Organoheterocyclic compounds	Indoles and derivatives	Indolyl carboxylic acids and derivatives	2.52	2.16	0.000	0.003
Thymidine 3'-monophosphate	367.06	neg	$C_{10}H_{15}N_2O_8P$	3.14	Nucleosides, nucleotides, and analogues	Ribonucleoside 3'-phosphates	Not Available	2.52	1.29	0.006	0.029
L-Tyrosine	146.06	pos	$C_9H_{11}NO_3$	2.21	Organic acids and derivatives	Carboxylic acids and derivatives	Amino acids, peptides, and analogues	2.51	1.20	0.002	0.012
2-Hydroxy-3-(4 hydroxyphenyl) propen-oic acid	539.12	neg	$C_9H_8O_4$	5.52	Benzenoids	Benzene and substituted derivatives	Phenylpyruvic acid derivatives	2.48	1.32	0.019	0.043
PE (15:0/P-16:0)	151.08	neg	$C_9H_{14}O_3$	4.16	Organic acids and derivatives	Keto acids and derivatives	Alpha-keto acids and derivatives	2.48	0.77	0.000	0.001
LysoPC (18:1(11Z))	522.36	pos	$C_{26}H_{52}NO_7P$	6.46	Lipids and lipid-like molecules	Glycerophospholipids	Glycerophosphocholines	2.48	0.77	0.007	0.046
DHAP (10:0)	325.14	pos	$C_{13}H_{25}O_7P$	3.21	Organic oxygen compounds	Organooxygen compounds	Carbonyl compounds	2.45	1.22	0.001	0.006
3,4,5-trihydroxy-6-{[3-(3-methoxyphenyl) prop-2-enoyl] oxy} oxane-2-carboxylic acid	375.07	neg	$C_{16}H_{18}O_9$	4.90	Phenylpropanoids and polyketides	Cinnamic acids and derivatives	Cinnamic acid esters	2.42	1.30	0.003	0.019
Gibberellin A72	345.13	neg	$C_{19}H_{24}O_7$	4.35	Lipids and lipid-like molecules	Prenol lipids	Diterpenoids	2.41	1.22	0.003	0.017
Benzoic acid	315.07	neg	$C_{13}H_{16}O_9$	1.69	Benzenoids	Benzene and substituted derivatives	Benzoic acids and derivatives	2.41	2.21	8.61×10^{-6}	0.0005

Vasicinone	185.07	pos	$C_{11}H_{10}N_2O_2$	4.22	-	-	-	2.40	1.30	0.009	0.045
4-hydroxy ketorolac	316.08	neg	$C_{15}H_{13}NO_4$	4.89	Organic oxygen compounds	Organooxygen compounds	Carbonyl compounds	2.37	0.79	0.013	0.043
PC (16:0/18:2(9Z,12Z))	758.57	pos	$C_{42}H_{80}NO_8P$	7.43	Lipids and lipid-like molecules	Glycerophospholipids	Glycerophosphocholines	2.36	1.13	0.009	0.052
PC (16:0/16:0)	734.57	pos	$C_{40}H_{80}NO_8P$	7.75	Lipids and lipid-like molecules	Glycerophospholipids	Glycerophosphocholines	2.35	1.15	0.005	0.037
PC (15:0/20:2(11Z,14Z))	684.50	pos	$C_{36}H_{72}NO_7P$	7.81	Lipids and lipid-like molecules	Glycerophospholipids	Glycerophosphoethanolamines	2.33	0.89	0.004	0.035
12-Oxo-20-trihydroxy-leukotriene B4	383.21	pos	$C_{20}H_{30}O_7$	5.97	Lipids and lipid-like molecules	Fatty Acyls	Eicosanoids	2.33	0.89	0.000	0.004
(±)-Enterolactone	297.11	neg	$C_{18}H_{18}O_4$	5.90	-	-	-	2.33	2.24	0.009	0.039
PE (14:0/P-16:0)	605.24	neg	$C_{35}H_{34}N_4O_6$	6.35	Organoheterocyclic compounds	Tetrapyrroles and derivatives	Chlorins	2.31	0.83	0.010	0.040
Isoleucyl-Alanine	185.13	pos	$C_9H_{18}N_2O_3$	2.98	Organic acids and derivatives	Carboxylic acids and derivatives	Amino acids, peptides, and analogues	2.31	0.83	0.004	0.046
Edulisin I	471.14	neg	$C_{28}H_{26}O_8$	6.155	Phenylpropanoids and polyketides	Coumarins and derivatives	Furanocoumarins	2.31	0.83	0.010	0.046
Licoisoflavone A	372.14	pos	$C_{20}H_{18}O_6$	1.94355	Phenylpropanoids and polyketides	Isoflavonoids	Isoflav-2-enes	2.31	0.82	0.017	0.048
4-hydroxy-3-(sulfooxy)benzoic acid	232.98	neg	$C_7H_6O_7S$	0.8989	Organic acids and derivatives	Organic sulfuric acids and derivatives	Arylsulfates	2.30	1.16	0.004	0.021
PSOROMIC ACID	357.06	neg	$C_{18}H_{14}O_8$	4.708217	-	-	-	2.28	0.81	0.005	0.023
8-Hydroxy-2-methoxy-6-methyl-1,4-naphthoquinone	263.06	neg	$C_{12}H_{10}O_4$	0.621817	Benzenoids	Naphthalenes	Naphthoquinones	2.27	1.15	0.001	0.007
2-Phenyl-1,3-dioxolane-4-methanol	405.15	neg	$C_{10}H_{12}O_3$	4.746583	Benzenoids	Benzene and substituted derivatives	Not Available	2.25	1.18	0.001	0.007
Isoleucyl-Glutamine	242.15	pos	$C_{11}H_{21}N_3O_4$	2.3483	Organic acids and derivatives	Carboxylic acids and derivatives	Amino acids, peptides, and analogues	2.25	1.25	0.017	0.078
Pseudobaptigenin	317.02	neg	$C_{16}H_{10}O_5$	5.7405	Phenylpropanoids and polyketides	Isoflavonoids	Isoflav-2-enes	2.25	1.28	0.017	0.059
DG (15:0/20:3(5Z,8Z,11Z)/0:0)	649.48	pos	$C_{38}H_{68}O_5$	7.290333	Lipids and lipid-like molecules	Glycerolipids	Diradylglycerols	2.25	1.14	0.014	0.071
3,4,5-trihydroxy-6-[2-(3-hydroxyprop-1-en-1-yl) phenoxy] oxane-2-carboxy lic acid	344.14	pos	$C_{15}H_{18}O_8$	3.386633	Organic oxygen compounds	Organooxygen compounds	Carbohydrates and carbohydrate conjugates	2.24	1.18	0.000	0.009
Glutamyltryptophan	356.12	pos	$C_{16}H_{19}N_3O_5$	0.976367	Organic acids and derivatives	Carboxylic acids and derivatives	Amino acids, peptides, and analogues	2.24	1.20	0.003	0.026

VIP = variable importance in the projection; pos = positive ion; neg = negative ion; FC = fold change; FDR = false discovery rate; I-2 = inulin-2, inulin addition level was 200 g/d per cow; - = the compound classification was unknown

Table S8 The significantly differential rumen metabolites (Top 50 of VIP value) between control and inulini-3 group

**Metabolite**	**M/Z**	**Mode**	**Formula**	**Retention time**	**HMDB Superclass**	**HMDB Class**	**HMDB Subclass**	**VIP**	**FC (I-3/Con)**	***P*-value**	**FDR**
Cer(d18:0/15:0)	526.52	pos	$C_{33}H_{67}NO_3$	7.75	-	-	-	4.92	0.42	0.001	0.015
Melibiose	377.09	neg	$C_{12}H_{22}O_{11}$	0.61	Organic oxygen compounds	Organooxygen compounds	Carbohydrates and carbohydrate conjugates	4.56	4.00	0.002	0.047
Cer (d18:0/13:0)	498.49	pos	$C_{31}H_{63}NO_3$	7.47	-	-	-	4.50	0.70	0.013	0.046
N-Acetyllactosamine	348.13	pos	$C_{14}H_{25}NO_{11}$	2.55	Organic oxygen compounds	Organooxygen compounds	Carbohydrates and carbohydrate conjugates	4.03	3.19	0.002	0.026
12-Oxo-20-trihydroxy-leukotriene B4	383.21	pos	$C_{20}H_{30}O_7$	5.97	Lipids and lipid-like molecules	Fatty Acyls	Eicosanoids	3.64	0.80	0.000	0.008
D-Fructose	179.06	neg	$C_6H_{12}O_6$	0.61	Organic oxygen compounds	Organooxygen compounds	Carbohydrates and carbohydrate conjugates	3.37	3.17	0.000	0.005
9,12,13-TriHOME	375.24	neg	$C_{18}H_{34}O_5$	6.39	Lipids and lipid-like molecules	Fatty Acyls	Fatty acids and conjugates	3.36	0.76	0.003	0.028
Apterin	469.13	neg	$C_{20}H_{24}O_{10}$	2.54	Phenylpropanoids and polyketides	Coumarins and derivatives	Furanocoumarins	3.29	2.22	0.004	0.025
9-O-Methylglyceofuran	369.13	pos	$C_{21}H_{20}O_6$	4.52	Phenylpropanoids and polyketides	Isoflavonoids	Furanoisoflavonoids	3.23	1.92	0.002	0.022
L-Tyrosine	146.06	pos	$C_9H_{11}NO_3$	2.21	Organic acids and derivatives	Carboxylic acids and derivatives	Amino acids, peptides, and analogues	3.03	1.37	0.000	0.006
(±)-Enterolactone	297.11	neg	$C_{18}H_{18}O_4$	5.90	-	-	-	2.93	2.48	0.000	0.006
5'-O-Methylmelledonal	464.23	pos	$C_{24}H_{30}O_8$	2.66	Lipids and lipid-like molecules	Prenol lipids	Sesquiterpenoids	2.86	1.46	0.001	0.016
17-phenyl-18,19,20-trinor-prostaglandin E2	407.19	neg	$C_{23}H_{30}O_5$	8.32	Lipids and lipid-like molecules	Fatty Acyls	Eicosanoids	2.75	0.77	0.002	0.035
LysoPE(0:0/15:0)	476.22	neg	$C_{20}H_{42}NO_7P$	3.02	Lipids and lipid-like molecules	Glycerophospholipids	Glycerophosphoethanola mines	2.72	2.16	0.043	0.113
Aucubin	364.16	pos	$C_{15}H_{22}O_9$	1.04	Lipids and lipid-like molecules	Prenol lipids	Terpene glycosides	2.72	1.55	0.017	0.046
Apiosylglucosyl 4-hydroxyben zoate	431.12	neg	$C_{18}H_{24}O_{12}$	1.69	Organic oxygen compounds	Organooxygen compounds	Carbohydrates and carbohydrate conjugates	2.72	1.65	0.010	0.046
Cis-3-Hexenyl pyruvate	151.08	neg	$C_9H_{14}O_3$	4.16	Organic acids and derivatives	Keto acids and derivatives	Alpha-keto acids and derivatives	2.70	1.30	0.000	0.005
Noralfentanil	294.22	pos	$C_{16}H_{24}N_2O_2$	3.36	Benzenoids	Benzene and substituted derivatives	Anilides	2.69	2.01	0.020	0.051
LysoPE (0:0/20:3(11Z,14Z,17Z))	548.30	neg	$C_{25}H_{46}NO_7P$	6.74	Lipids and lipid-like molecules	Glycerophospholipids	Glycerophosphoethanola mines	2.67	0.60	0.006	0.034
N-Acetyl-D-phenylalanine	206.08	neg	$C_{11}H_{13}NO_3$	3.68	-	-	-	2.62	1.31	0.002	0.018
Monascoflavin	403.18	neg	$C_{21}H_{26}O_5$	5.09	Organic oxygen compounds	Organooxygen compounds	Carbonyl compounds	2.58	1.48	0.002	0.018

Benzoic acid	315.07	neg	$C_{13}H_{16}O_9$	1.69	Benzenoids	Benzene and substituted derivatives	Benzoic acids and derivatives	2.55	2.96	0.004	0.011
N-Acetyl-L-glutamate 5-semialdehyde	208.04	neg	$C_7H_{11}NO_4$	3.93	Organic acids and derivatives	Carboxylic acids and derivatives	Amino acids, peptides, and analogues	2.54	1.59	0.012	0.041
11-keto Fluprostenol	457.18	pos	$C_{23}H_{27}F_3O_6$	2.59	-	-	-	2.50	1.37	0.013	0.045
DHAP (10:0)	325.14	pos	$C_{13}H_{25}O_7P$	3.21	Organic oxygen compounds	Organooxygen compounds	Carbonyl compounds	2.50	2.60	0.004	0.041
Gibberellin A72	345.13	neg	$C_{19}H_{24}O_7$	4.35	Lipids and lipid-like molecules	Prenol lipids	Diterpenoids	2.47	1.34	0.002	0.015
LPC (18:1)	544.34	pos	$C_{26}H_{52}NO_7P$	6.45	-	-	-	2.44	0.85	0.000	0.009
Retinoyl b-glucuronide	509.28	pos	$C_{26}H_{36}O_8$	1.90	Lipids and lipid-like molecules	Prenol lipids	Terpene glycosides	2.44	1.42	0.018	0.049
N-Carboxyacetyl-D-phenylalanine	252.09	pos	$C_{12}H_{13}NO_5$	3.79	Organic acids and derivatives	Carboxylic acids and derivatives	Amino acids, peptides, and analogues	2.42	1.27	0.004	0.032
LysoPC (18:1(11Z))	522.36	pos	$C_{26}H_{52}NO_7P$	6.46	Lipids and lipid-like molecules	Glycerophospholipids	Glycerophosphocholines	2.42	0.85	0.001	0.012
L-Glutamate	146.04	neg	$C_5H_9NO_4$	0.61	Organoheterocyclic compounds	Indoles and derivatives	Indolyl carboxylic acids and derivatives	2.42	2.22	0.000	0.005
Tricin 7-diglucuronoside	681.13	neg	$C_{29}H_{30}O_{19}$	3.74	Phenylpropanoids and polyketides	Flavonoids	Flavonoid glycosides	2.41	1.36	0.016	0.043
LysoPC (18:1(9Z))	544.34	pos	$C_{26}H_{52}NO_7P$	6.76	Lipids and lipid-like molecules	Glycerophospholipids	Glycerophosphocholines	2.41	0.81	0.001	0.014
PC (15:0/20:2(11Z,14Z))	794.57	pos	$C_{43}H_{82}NO_8P$	7.53	Lipids and lipid-like molecules	Glycerophospholipids	Glycerophosphocholines	2.39	2.41	0.004	0.027
Oxypinnatanine	295.07	neg	$C_{10}H_{16}N_2O_6$	6.18	Organic acids and derivatives	Carboxylic acids and derivatives	Amino acids, peptides, and analogues	2.37	0.71	0.012	0.051
LysoPC (18:0)	568.36	neg	$C_{26}H_{54}NO_7P$	6.61	Lipids and lipid-like molecules	Glycerophospholipids	Glycerophosphocholines	2.36	0.79	0.002	0.036
Zeranol	343.15	neg	$C_{18}H_{26}O_5$	5.09	Phenylpropanoids and polyketides	Macrolides and analogues	Not Available	2.35	1.45	0.004	0.027
Glutamyltryptophan	356.12	pos	$C_{16}H_{19}N_3O_5$	0.98	Organic acids and derivatives	Carboxylic acids and derivatives	Amino acids, peptides, and analogues	2.32	1.34	0.017	0.077
Phosphocholine	184.07	pos	$C_5H_{14}NO_4P$	6.42	-	-	-	2.30	0.76	0.006	0.040
Phenylalanyl-Lysine	338.17	neg	$C_{15}H_{23}N_3O_3$	3.45	Organic acids and derivatives	Carboxylic acids and derivatives	Amino acids, peptides, and analogues	2.30	0.78	2.79×10^{-5}	0.002
PC (16:0/18:1(11Z))	782.57	pos	$C_{42}H_{82}NO_8P$	7.73	Lipids and lipid-like molecules	Glycerophospholipids	Glycerophosphocholines	2.29	2.55	0.003	0.039
4-Pentenyl acetate	149.06	neg	$C_7H_{12}O_2$	4.16	Organic acids and derivatives	Carboxylic acids and derivatives	Carboxylic acid derivatives	2.26	1.21	5.12×10^{-5}	0.003
Gamma-linolenyl carnitine	458.27	neg	$C_{25}H_{43}NO_4$	6.32	-	-	-	2.23	0.75	0.004	0.025
Tyrosyl-Isoleucine	277.16	pos	$C_{15}H_{22}N_2O_4$	3.27	Organic acids and derivatives	Carboxylic acids and derivatives	Amino acids, peptides, and analogues	2.22	0.82	0.007	0.047

Isoleucyl-Alanine	185.13	pos	$C_9H_{18}N_2O_3$	2.98	Organic acids and derivatives	Carboxylic acids and derivatives	Amino acids, peptides, and analogues	2.19	0.78	0.005	0.032
Gibberellin A22	345.13	neg	$C_{19}H_{22}O_6$	4.62	Lipids and lipid-like molecules	Prenol lipids	Diterpenoids	2.18	1.22	0.001	0.008
DG (18:1n7/0:0/18:2n6)	633.54	pos	$C_{40}H_{72}O_5$	7.14	Lipids and lipid-like molecules	Glycerolipids	Diradylglycerols	2.16	0.80	0.007	0.047
Valyl-Isoleucine	213.16	pos	$C_{11}H_{22}N_2O_3$	4.66	Organic acids and derivatives	Carboxylic acids and derivatives	Amino acids, peptides, and analogues	2.13	0.81	0.000	0.006
CE (11:1D3)	669.56	neg	$C_{47}H_{76}O_3$	7.19	Lipids and lipid-like molecules	Steroids and steroid derivatives	Steroid esters	2.04	0.80	0.012	0.042
Sorbitan oleate	901.63	neg	$C_{24}H_{44}O_6$	6.58	Lipids and lipid-like molecules	Fatty Acyls	Fatty acid esters	2.01	0.82	0.018	0.046

VIP = variable importance in the projection; pos = positive ion; neg = negative ion; FC = fold change; FDR = false discovery rate; I-3 = inulin-3, inulin addition level was 300 g/d per cow; - = the compound classification was unknown

Table S9 The significantly differential rumen metabolites (Top 50 of VIP value) between control and inulini-4 group

**Metabolite**	**M/Z**	**Mode**	**Formula**	**Retention time**	**HMDB Superclass**	**HMDB Class**	**HMDB Subclass**	**VIP**	**FC (I-4/Con)**	***P*-value**	**FDR**
Cer(d18:0/15:0)	526.52	pos	$C_{33}H_{67}NO_3$	7.75	-	-	-	4.32	0.24	0.010	0.038
Melibiose	377.09	neg	$C_{12}H_{22}O_{11}$	0.61	Organic oxygen compounds	Organooxygen compounds	Carbohydrates and carbohydrate conjugates	3.83	3.78	0.000	0.003
Silymonin	513.14	neg	$C_{25}H_{24}O_9$	3.12	Organoheterocyclic compounds	Benzodioxoles	Not Available	3.62	0.46	0.013	0.052
12-Oxo-20-trihydroxy-leukotriene B4	383.21	pos	$C_{20}H_{30}O_7$	5.97	Lipids and lipid-like molecules	Fatty Acyls	Eicosanoids	3.55	0.59	0.011	0.032
Cer(d18:0/13:0)	498.49	pos	$C_{31}H_{63}NO_3$	7.47	-	-	-	3.15	0.55	0.005	0.028
Salvianolic acid D	339.05	neg	$C_{18}H_{12}O_7$	4.07	Organoheterocyclic compounds	Benzoxepines	Dibenzoxepines	3.13	0.56	0.035	0.101
N-Acetyllactosamine	348.13	pos	$C_{14}H_{25}NO_{11}$	2.55	Organic oxygen compounds	Organooxygen compounds	Carbohydrates and carbohydrate conjugates	3.12	2.16	0.002	0.018
Cysteinyl-Arginine	314.07	neg	$C_9H_{19}N_5O_3S$	3.34	Organic acids and derivatives	Carboxylic acids and derivatives	Amino acids, peptides, and analogues	2.95	0.67	0.008	0.038
Apterin	469.13	neg	$C_{20}H_{24}O_{10}$	2.54	Phenylpropanoids and polyketides	Coumarins and derivatives	Furanocoumarins	2.81	1.65	0.003	0.020
3,4,5-trihydroxy-6-[2-(3-hydroxyprop-1-en-1-yl) phenoxy] oxa-ne-2-carboxylic acid	344.14	pos	$C_{15}H_{18}O_8$	3.39	Organic oxygen compounds	Organooxygen compounds	Carbohydrates and carbohydrate conjugates	2.78	1.29	0.000	0.005
9,12,13-TriHOME	375.24	neg	$C_{18}H_{34}O_5$	6.39	Lipids and lipid-like molecules	Fatty Acyls	Fatty acids and conjugates	2.76	0.60	0.004	0.021

L-Tyrosine	146.06	pos	$C_9H_{11}NO_3$	2.21	Organic acids and derivatives	Carboxylic acids and derivatives	Amino acids, peptides, and analogues	2.64	1.29	0.003	0.021
Pseudobaptigenin	317.02	neg	$C_{16}H_{10}O_5$	5.74	Phenylpropanoids and polyketides	Isoflavonoids	Isoflav-2-enes	2.61	1.32	0.007	0.034
Demethyloleuropein	507.15	neg	$C_{24}H_{30}O_{13}$	4.34	Phenylpropanoids and polyketides	Flavonoids	Flavonoid glycosides	2.58	0.58	0.014	0.054
N-(1-Deoxy-1-fructosyl) alanine	296.10	neg	$C_9H_{17}NO_7$	0.61	Organic oxygen compounds	Organooxygen compounds	Carbohydrates and carbohydrate conjugates	2.53	1.24	0.003	0.018
LysoPC (18:1(11Z))	522.36	pos	$C_{26}H_{52}NO_7P$	6.46	Lipids and lipid-like molecules	Glycerophospholipids	Glycerophosphocholin es	2.53	0.74	0.006	0.031
Ginkgolide C	475.10	neg	$C_{20}H_{24}O_{11}$	5.01	Lipids and lipid-like molecules	Prenol lipids	Terpene lactones	2.52	0.74	0.029	0.088
Benzoic acid	315.07	neg	$C_{13}H_{16}O_9$	1.69	Benzenoids	Benzene and substituted derivatives	Benzoic acids and derivatives	2.50	1.32	0.006	0.036
17-phenyl-18,19,20-trinor-prostaglandin E2	407.19	neg	$C_{23}H_{30}O_5$	8.32	Lipids and lipid-like molecules	Fatty Acyls	Eicosanoids	2.50	0.73	0.003	0.010
Medicarpin 3-O-(6'-malonylglucoside)	539.12	neg	$C_{25}H_{26}O_{12}$	5.22	Phenylpropanoids and polyketides	Isoflavonoids	Furanoisoflavonoids	2.46	1.33	0.036	0.102
Indolelactic acid	204.07	neg	$C_{11}H_{11}NO_3$	4.26	Organoheterocyclic compounds	Indoles and derivatives	Indolyl carboxylic acids and derivatives	2.44	1.16	2.87×10^{-5}	0.001
Tyrosyl-Isoleucine	277.16	pos	$C_{15}H_{22}N_2O_4$	3.27	Organic acids and derivatives	Carboxylic acids and derivatives	Amino acids, peptides, and analogues	2.40	0.76	0.013	0.047
D-Fructose	179.06	neg	$C_6H_{12}O_6$	0.61	Organic oxygen compounds	Organooxygen compounds	Carbohydrates and carbohydrate conjugates	2.37	2.37	0.014	0.048
Crosatoside B	491.18	neg	$C_{20}H_{30}O_{11}$	4.12	Organic oxygen compounds	Organooxygen compounds	Carbohydrates and carbohydrate conjugates	2.35	0.60	0.012	0.050
PC (15:0/20:2(11Z,14Z))	794.57	pos	$C_{43}H_{82}NO_8P$	7.53	Lipids and lipid-like molecules	Glycerophospholipids	Glycerophosphocholin es	2.32	1.24	0.002	0.014
5'-CMP	322.04	neg	$C_9H_{14}N_3O_8P$	0.64	-	-	-	2.29	1.15	0.002	0.015
PC (16:0/18:1(11Z))	782.57	pos	$C_{42}H_{82}NO_8P$	7.73	Lipids and lipid-like molecules	Glycerophospholipids	Glycerophosphocholin es	2.26	1.21	0.002	0.012
LysoPC (18:0)	568.36	neg	$C_{26}H_{54}NO_7P$	6.61	Lipids and lipid-like molecules	Glycerophospholipids	Glycerophosphocholin es	2.19	0.75	0.009	0.039
PE (15:0/P-16:0)	684.50	pos	$C_{36}H_{72}NO_7P$	7.81	Lipids and lipid-like molecules	Glycerophospholipids	Glycerophosphoethano lamines	2.12	1.19	0.025	0.088
Valyl-Isoleucine	213.16	pos	$C_{11}H_{22}N_2O_3$	4.66	Organic acids and derivatives	Carboxylic acids and derivatives	Amino acids, peptides, and analogues	2.11	0.80	0.003	0.011
PE (14:0/P-16:0)	670.48	pos	$C_{35}H_{70}NO_7P$	7.68	Lipids and lipid-like molecules	Glycerophospholipids	Glycerophosphoethano lamines	2.10	1.17	0.017	0.068
Phenylalanyl-Lysine	338.17	neg	$C_{15}H_{23}N_3O_3$	3.45	Organic acids and derivatives	Carboxylic acids and derivatives	Amino acids, peptides, and analogues	2.08	0.38	0.005	0.035

DHAP (10:0)	325.14	pos	$C_{13}H_{25}O_7P$	3.21	Organic oxygen compounds	Organooxygen compounds	Carbonyl compounds	2.03	1.18	0.014	0.045
FAHFA (18:1(9Z)/12-O-18:0)	609.49	pos	$C_{36}H_{68}O_4$	6.92	Lipids and lipid-like molecules	Fatty Acyls	Fatty acids and conjugates	2.02	1.20	0.049	0.137
L-Glutamate	146.04	neg	$C_5H_9NO_4$	0.61	Organoheterocyclic compounds	Indoles and derivatives	Indolyl carboxylic acids and derivatives	2.00	1.26	0.002	0.030
Physagulin F	589.27	neg	$C_{30}H_{40}O_9$	4.86	Lipids and lipid-like molecules	Steroids and steroid derivatives	Steroid lactones	1.93	1.18	0.046	0.121
Inosine 5'-monophosphate (IMP)	347.04	neg	$C_{10}H_{13}N_4O_8P$	0.78	-	-	-	1.91	1.11	0.006	0.030
8-Hydroxy-2-methoxy-6-methyl-1,4-naphthoquinone	263.06	neg	$C_{12}H_{10}O_4$	0.62	Benzenoids	Naphthalenes	Naphthoquinones	1.88	1.12	0.000	0.004
Isoleucyl-Alanine	185.13	pos	$C_9H_{18}N_2O_3$	2.98	Organic acids and derivatives	Carboxylic acids and derivatives	Amino acids, peptides, and analogues	1.86	0.75	0.004	0.040
Lucidenic acid N	459.27	neg	$C_{27}H_{40}O_6$	4.89	Lipids and lipid-like molecules	Prenol lipids	Triterpenoids	1.84	1.10	0.011	0.046
Gamma-Glutamylproline	289.10	neg	$C_{10}H_{16}N_2O_5$	1.52	Organic acids and derivatives	Carboxylic acids and derivatives	Amino acids, peptides, and analogues	1.80	1.12	0.003	0.018
Adenosine 3'-monophosphate	348.07	pos	$C_{10}H_{14}N_5O_7P$	0.93	-	-	-	1.78	1.10	0.017	0.069
(S)-3-Sulfonatolactate	168.98	neg	$C_3H_6O_6S$	0.73	Organic acids and derivatives	Hydroxy acids and derivatives	Alpha hydroxy acids and derivatives	1.75	1.12	0.006	0.032
Crocin 3	633.26	neg	$C_{32}H_{44}O_{14}$	4.12	Lipids and lipid-like molecules	Prenol lipids	Diterpenoids	1.74	1.12	0.020	0.070
Ascorbic acid	391.02	pos	$C_6H_8O_6$	1.07	Organoheterocyclic compounds	Dihydrofurans	Furanones	1.70	1.12	0.030	0.100
2-amino-octadecanoic acid	300.29	pos	$C_{18}H_{37}NO_2$	6.24	-	-	-	1.57	1.11	0.013	0.058
4-Pentenyl acetate	149.06	neg	$C_7H_{12}O_2$	4.16	Organic acids and derivatives	Carboxylic acids and derivatives	Carboxylic acid derivatives	1.52	1.11	0.036	0.102
(±)-Enterolactone	297.11	neg	$C_{18}H_{18}O_4$	5.90	-	-	-	1.49	1.17	0.004	0.032
2-Phenyl-1,3-dioxolane-4-methanol	405.15	neg	$C_{10}H_{12}O_3$	4.75	Benzenoids	Benzene and substituted derivatives	Not Available	1.48	1.11	0.028	0.087
Neohesperidin dihydrochalcone	657.20	neg	$C_{28}H_{36}O_{15}$	5.74	Phenylpropanoids and polyketides	Flavonoids	Flavonoid glycosides	1.22	0.44	0.002	0.015

VIP = variable importance in the projection; pos = positive ion; neg = negative ion; FC = fold change; FDR = false discovery rate; I-4 = inulin-4, inulin addition level was 400 g/d per cow; - = the compound classification was unknown

Table S10 Differential rumen metabolites (average relative abundance) among control and 4 inulin groups

**Metabolite**	**Groups (n = 6)**					**SEM**	***P*-value**	**FDR**
	**Con**	**I-1**	**I-2**	**I-3**	**I-4**			
D-Fructose	6.23^{c}	6.04^{c}	7.37^{a}	7.58^{a}	7.07^{b}	0.319	0.000555	0.01783
Melibiose	6.42^{b}	7.19^{ab}	7.40^{a}	7.65^{a}	7.23^{ab}	0.207	0.000467	0.00584
N-Acetyllactosamine	4.25^{b}	4.46^{b}	5.32^{a}	5.64^{a}	5.01^{ab}	0.259	0.003654	0.01812
L-Glutamate	4.74^{c}	4.75^{b}	5.65^{b}	6.05^{a}	5.76^{b}	0.184	0.002508	0.0163
L-Tyrosine	4.68^{c}	5.69^{b}	5.71^{b}	6.05^{a}	5.42^{b}	0.230	0.004878	0.02362
Benzoic acid	4.04^{c}	4.13^{c}	5.23^{b}	5.74^{a}	5.23^{b}	0.335	0.008736	0.02264
DHAP (10:0)	5.39^{c}	6.14^{b}	6.17^{b}	6.75^{a}	6.18^{b}	0.218	0.01603	0.04488
PC (15:0/20:2(11Z,14Z))	4.39^{b}	4.76^{b}	5.84^{a}	5.69^{a}	5.09^{ab}	0.274	0.01669	0.04581
PC (16:0/18:1(11Z))	4.46^{b}	4.41^{b}	5.50^{a}	5.50^{a}	5.01^{ab}	0.239	0.005974	0.02765
(±)-Enterolactone	5.90^{c}	6.27^{b}	7.63^{a}	7.31^{a}	6.55^{b}	0.323	0.000628	0.00742
Cer(d18:0/15:0)	6.41^{a}	6.45^{a}	4.73^{c}	4.24^{c}	5.59^{b}	0.441	0.000396	0.004273
Cer(d18:0/13:0)	6.04^{a}	5.87^{ab}	4.84^{c}	4.66^{c}	5.32^{b}	0.273	0.000859	0.001358
12-Oxo-20-trihydroxy-leukotriene B4	7.56^{a}	7.45^{a}	5.49^{c}	5.49^{c}	5.95^{bc}	0.465	0.001201	0.01861
17-phenyl-18,19,20-trinor-prostaglandin E2	6.61^{a}	6.57^{a}	5.59^{b}	5.49^{b}	5.60^{b}	0.252	0.01847	0.03893
LysoPC (18:1(11Z))	7.22^{a}	7.66^{a}	6.04^{b}	6.14^{b}	6.77^{ab}	0.310	0.000679	0.01775
LysoPC (18:0)	6.90^{a}	6.31^{ab}	5.01^{b}	4.35^{c}	4.60^{c}	0.498	0.000514	0.01386
9,12,13-TriHOME	6.81^{a}	6.22^{a}	5.47^{b}	5.29^{b}	5.84^{b}	0.273	0.003217	0.04575
Valyl-Isoleucine	6.52^{a}	6.14^{a}	4.50^{b}	4.85^{b}	4.87^{b}	0.401	0.01158	0.03761
Phenylalanyl-Lysine	6.47^{a}	6.35^{a}	4.65^{b}	4.58^{b}	4.79^{b}	0.427	0.003746	0.02055

DHAP = dihydroxyacetone phosphate; PC = phosphatidyl choline; Cer = ceramide; LysoPC = lysophosphatidycholine; FDR = false discovery rate; - = the compound classification was unknown; Con = control group; I-1 = inulin-1 group, the inulin addition level was 100 g/d per cow; I-2 = inulin-2 group, the inulin addition level was 200 g/d per cow; I-3 = inulin-3 group, the inulin addition level was 300 g/d per cow; I-4 = inulin-4 group, the inulin addition level was 400 g/d per cow; $^{a, b, c}$ = within a row, different letters differed significantly (FDR-adjusted $P < 0.05$).

Table S11 Correlation analysis between significantly differential ruminal bacteria and metabolites

**Bacteria**	**Metabolites**	**R**	**FDR**
Muribaculaceae	D-Fructose	0.4379	0.0481
Bifidobacterium	D-Fructose	0.2007	0.4348
Lachnospiraceae_NK3A20_group	D-Fructose	0.2677	0.2471
Prevotella	D-Fructose	0.4123	0.0542
Lactobacillus	D-Fructose	0.3088	0.2241
Succinivibrionaceae_UCG-001	D-Fructose	0.3974	0.0711
Eubacterium_ruminantium_group	D-Fructose	0.4024	0.0843
Lachnospiraceae_NK3A20_group	N-Acetyllactosamine	0.4296	0.0578
Rikenellaceae_RC9_gut_group	N-Acetyllactosamine	0.4097	0.0779
Eubacterium_ruminantium_group	N-Acetyllactosamine	0.4489	0.0568
Bifidobacterium	Melibiose	0.6104	0.0142
Prevotella	Melibiose	0.3326	0.2456
Butyrivibrio	Melibiose	0.3146	0.1723
Lactobacillus	Melibiose	0.5812	0.0331

Rikenellaceae_RC9_gut_group	Melibiose	0.2781	0.3159
Christensenellaceae_R-7_group	Melibiose	0.4633	0.0572
Clostridia_UCG-014	Melibiose	-0.3468	0.1021
Streptococcus	Melibiose	-0.5236	0.0463
Staphylococcus	Melibiose	-0.3778	0.0908
Neisseriaceae	Melibiose	-0.5056	0.0442
Escherichia-Shigella	Melibiose	-0.5942	0.0245
Muribaculaceae	L-Glutamate	0.2687	0.4347
Bifidobacterium	L-Glutamate	0.4444	0.0517
Christensenellaceae_R-7_group	L-Glutamate	0.3373	0.2263
Butyrivibrio	L-Glutamate	0.6069	0.0204
Staphylococcus	L-Glutamate	-0.4454	0.0428
Neisseriaceae	L-Glutamate	-0.4394	0.0551
Muribaculaceae	L-Tyrosine	0.4230	0.0199
Bifidobacterium	L-Tyrosine	0.6143	0.0307
Bacteroidales_RF16_group	L-Tyrosine	0.3508	0.0810
Lactobacillus	L-Tyrosine	0.5935	0.0268
Bacteroidales_BS11_gut_group	L-Tyrosine	0.0397	0.0841
Streptococcus	L-Tyrosine	-0.5215	0.0458
Staphylococcus	L-Tyrosine	-0.4908	0.0424
Neisseriaceae	L-Tyrosine	-0.4702	0.0532
Escherichia-Shigella	L-Tyrosine	-0.5196	0.0406
Muribaculaceae	Benzoic acid	0.3162	0.2507
Prevotella	Benzoic acid	0.3053	0.2114
Butyrivibrio	Benzoic acid	0.5806	0.0357
Lactobacillus	Benzoic acid	0.3247	0.1065
Rikenellaceae_RC9_gut_group	Benzoic acid	0.4184	0.0687
Christensenellaceae_R-7_group	Benzoic acid	0.4561	0.0529
Streptococcus	Benzoic acid	-0.5773	0.0328
Staphylococcus	Benzoic acid	-0.3834	0.1068
Neisseriaceae	Benzoic acid	-0.3137	0.2037
Escherichia-Shigella	Benzoic acid	-0.6065	0.0204
Muribaculaceae	DHAP (10:0)	0.3105	0.1243
Lachnospiraceae_NK3A20_group	DHAP (10:0)	0.3845	0.0616
Prevotella	DHAP (10:0)	0.3157	0.0818
Butyrivibrio	DHAP (10:0)	0.4269	0.0586
Christensenellaceae_R-7_group	DHAP (10:0)	0.4021	0.0633
Oscillospira	DHAP (10:0)	-0.3702	0.1510
Bifidobacterium	(±)-Enterolactone	0.2552	0.3609
Lachnospiraceae_NK3A20_group	(±)-Enterolactone	0.3023	0.1407
Streptococcus	(±)-Enterolactone	-0.3637	0.2098
Staphylococcus	(±)-Enterolactone	-0.3057	0.2112
Oscillospira	(±)-Enterolactone	-0.3452	0.2021
Escherichia-Shigella	(±)-Enterolactone	0.2013	0.3541
Muribaculaceae	Cer(d18:0/15:0)	-0.5140	0.0443
Bifidobacterium	Cer(d18:0/15:0)	-0.4655	0.0574
Prevotella	Cer(d18:0/15:0)	-0.4854	0.0587
Lactobacillus	Cer(d18:0/15:0)	-0.5202	0.0410
Clostridia_UCG-014	Cer(d18:0/15:0)	0.3970	0.0638
Streptococcus	Cer(d18:0/15:0)	0.5616	0.0372

Staphylococcus	Cer(d18:0/15:0)	0.5768	0.0341
Neisseriaceae	Cer(d18:0/15:0)	0.4714	0.0557
Escherichia-Shigella	Cer(d18:0/15:0)	0.4319	0.0545
Muribaculaceae	Cer(d18:0/13:0)	-0.5073	0.0475
Bifidobacterium	Cer(d18:0/13:0)	-0.4633	0.0579
Prevotella	Cer(d18:0/13:0)	-0.4909	0.0487
Butyrivibrio	Cer(d18:0/13:0)	-0.4426	0.0539
Lactobacillus	Cer(d18:0/13:0)	-0.5154	0.0429
Streptococcus	Cer(d18:0/13:0)	0.5851	0.0349
Neisseriaceae	Cer(d18:0/13:0)	0.4847	0.0460
Escherichia-Shigella	Cer(d18:0/13:0)	0.4163	0.0618
Staphylococcus	Cer(d18:0/13:0)	0.4835	0.0476
Muribaculaceae	12-Oxo-20-trihydroxy-leukotriene B4	-0.4692	0.0396
Bifidobacterium	12-Oxo-20-trihydroxy-leukotriene B4	-0.4225	0.0485
Butyrivibrio	12-Oxo-20-trihydroxy-leukotriene B4	0.4020	0.0499
Lactobacillus	12-Oxo-20-trihydroxy-leukotriene B4	0.4128	0.0525
Christensenellaceae_R-7_group	12-Oxo-20-trihydroxy-leukotriene B4	0.4263	0.0669
Streptococcus	12-Oxo-20-trihydroxy-leukotriene B4	0.4312	0.0435
Staphylococcus	12-Oxo-20-trihydroxy-leukotriene B4	0.4449	0.0427
Ruminococcaceae	12-Oxo-20-trihydroxy-leukotriene B4	0.4645	0.0562
Neisseriaceae	12-Oxo-20-trihydroxy-leukotriene B4	0.4528	0.0563
Escherichia-Shigella	12-Oxo-20-trihydroxy-leukotriene B4	0.5402	0.0304
Muribaculaceae	17-phenyl-18,19,20-trinor-prostaglandin E2	-0.5058	0.0405
Bifidobacterium	17-phenyl-18,19,20-trinor-prostaglandin E2	-0.4844	0.0433
Prevotella	17-phenyl-18,19,20-trinor-prostaglandin E2	-0.4022	0.0584
Butyrivibrio	17-phenyl-18,19,20-trinor-prostaglandin E2	-0.4152	0.0527
Lactobacillus	17-phenyl-18,19,20-trinor-prostaglandin E2	-0.4177	0.0535
Streptococcus	17-phenyl-18,19,20-trinor-prostaglandin E2	0.4362	0.0508
Staphylococcus	17-phenyl-18,19,20-trinor-prostaglandin E2	0.4783	0.0482
Escherichia-Shigella	17-phenyl-18,19,20-trinor-prostaglandin E2	0.6045	0.0304
Muribaculaceae	LysoPC(18:0)	-0.4958	0.0304
Bifidobacterium	LysoPC(18:0)	-0.4631	0.0420
Lachnospiraceae_NK3A20_group	LysoPC(18:0)	-0.4551	0.0445
Butyrivibrio	LysoPC(18:0)	-0.4711	0.0460
Bacteroidales_RF16_group	LysoPC(18:0)	-0.4205	0.0502
Clostridia_UCG-014	LysoPC(18:0)	0.4286	0.0495
Streptococcus	LysoPC(18:0)	0.4174	0.0503
Bifidobacterium	LysoPC(18:1(11Z))	-0.4880	0.0465
Lachnospiraceae_NK3A20_group	LysoPC(18:1(11Z))	-0.5099	0.0407
Christensenellaceae_R-7_group	LysoPC(18:1(11Z))	-0.4237	0.0669
Clostridia_UCG-014	LysoPC(18:1(11Z))	0.4301	0.0426
Neisseriaceae	LysoPC(18:1(11Z))	0.4496	0.0486
Streptococcus	LysoPC(18:1(11Z))	0.4239	0.0492
Lachnospiraceae_NK3A20_group	9,12,13-TriHOME	-0.4229	0.0650
Butyrivibrio	9,12,13-TriHOME	-0.4031	0.0582
Christensenellaceae_R-7_group	9,12,13-TriHOME	-0.4137	0.0551
Ruminococcaceae	9,12,13-TriHOME	0.3841	0.0856
Neisseriaceae	9,12,13-TriHOME	0.3978	0.0736
Streptococcus	Valyl-Isoleucine	0.3823	0.1142
Staphylococcus	Valyl-Isoleucine	0.4324	0.0710

Escherichia-Shigella	Valyl-Isoleucine	0.5414	0.0417
Streptococcus	Phenylalanyl-Lysine	0.3662	0.149
Staphylococcus	Phenylalanyl-Lysine	0.4082	0.0851
Ruminococcaceae	Phenylalanyl-Lysine	0.0422	0.0730
Escherichia-Shigella	Phenylalanyl-Lysine	0.5273	0.0471

R = Correlation coefficient; FDR = false discovery rate

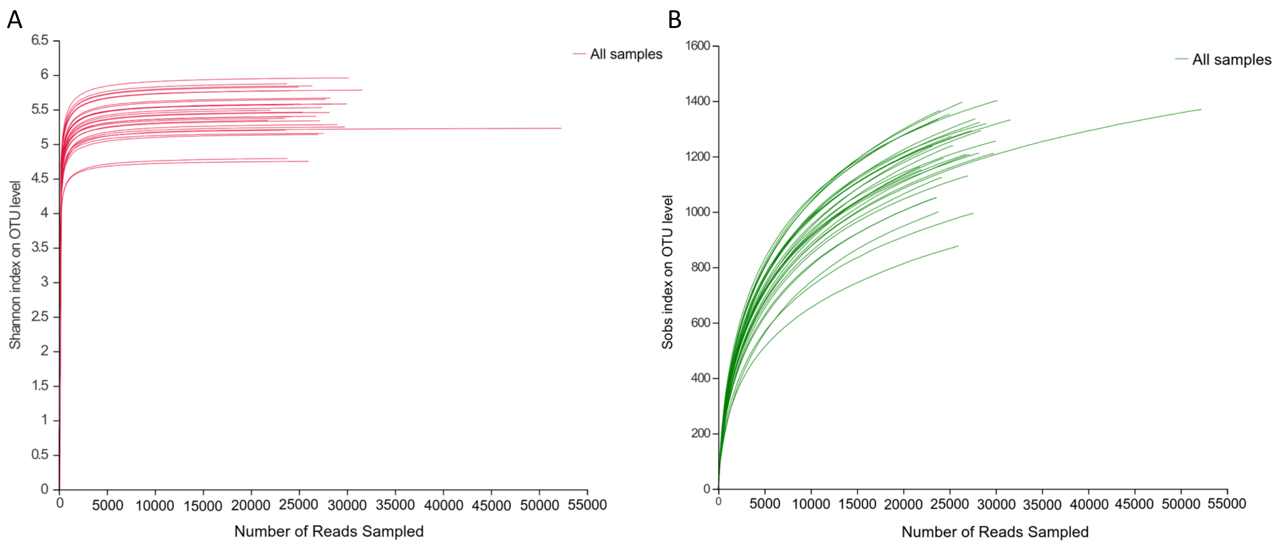

Fig. S1 Rarefaction curves based on (A)Shannon and (B) Sobs indexes

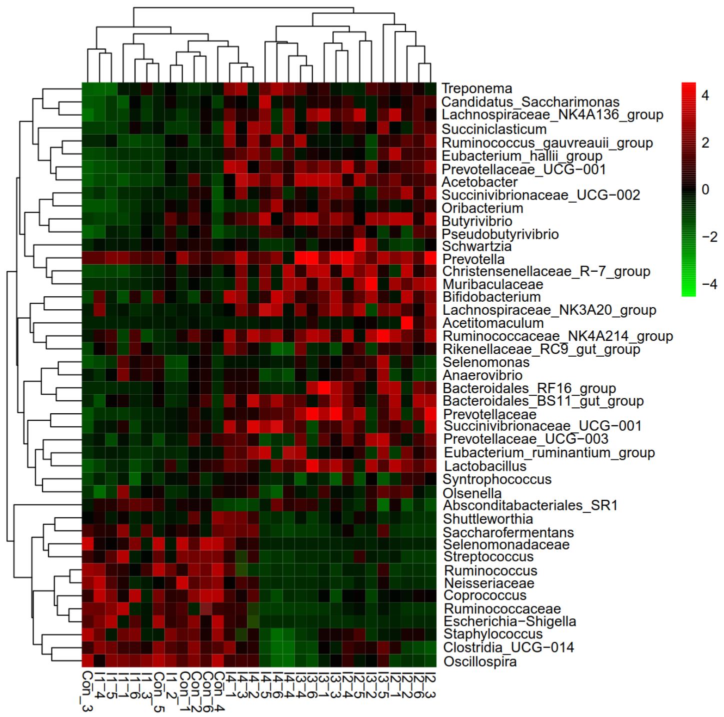

Fig. S2 Hierarchical clustering analysis (HCA) of the relative abundances of ruminal bacteria on the level of genera between control and different inulin addition groups. Each row represents a genus of bacteria and each column represents a sample. The color in each cell means the relative abundance of this bacteria measured in rumen, red represents high level of relative abundance while green means the low relative abundance. Con = control group; I-1 = inulin-1 group, the inulin addition level was 100 g/d per cow; I-2 = inulin-2 group, the inulin addition level was 200 g/d per cow; I-3 = inulin-3 group, the inulin addition level was 300 g/d per cow; I-4 = inulin-4 group, the inulin addition level was 400 g/d per cow.

A

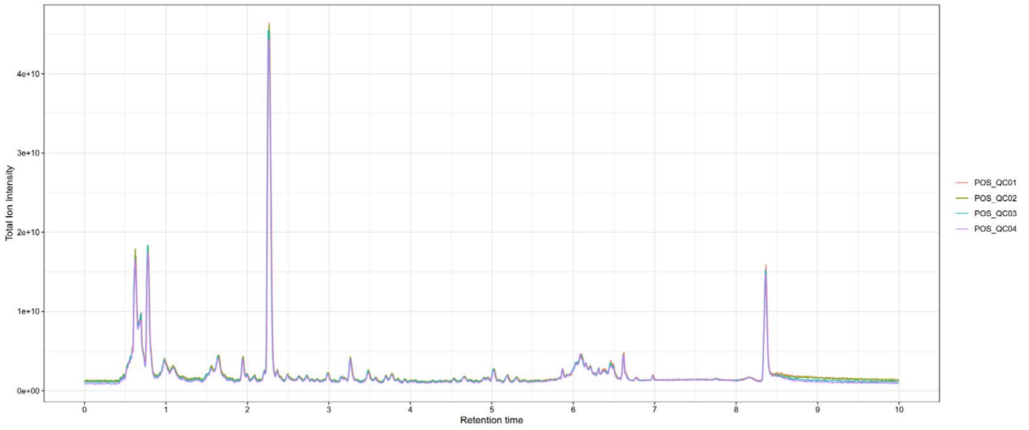

B

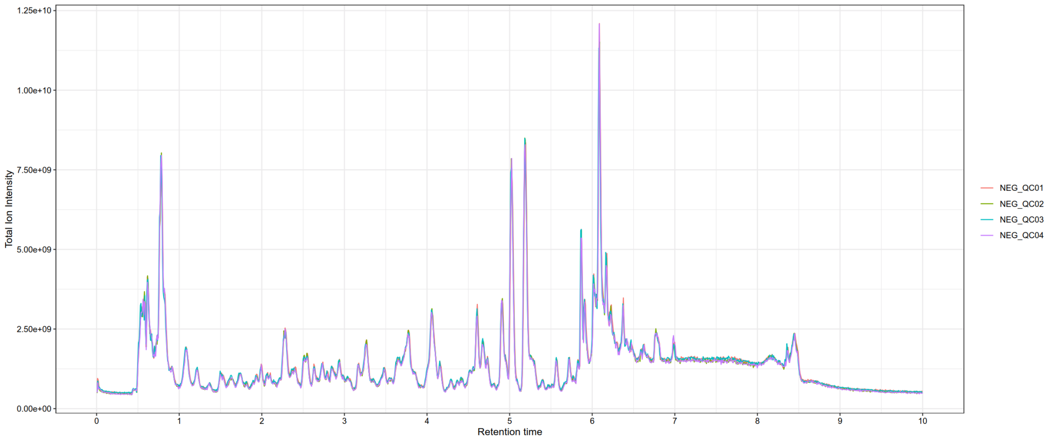

Fig. S3 Total ion chromatogram (TIC) of the quality control (QC) sample in the (A) positive and (B) negative ion modes.

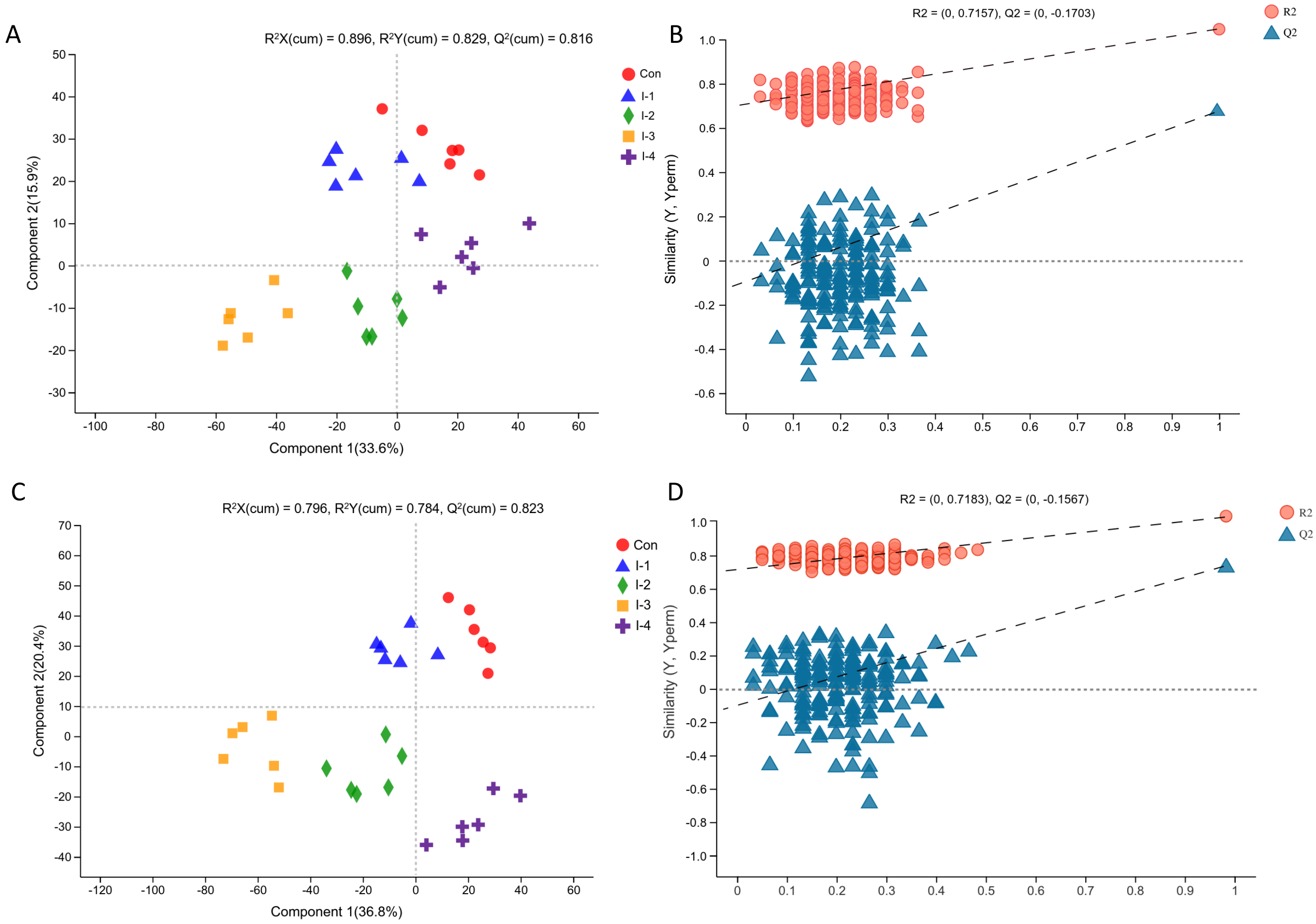

Fig. S4 Partial least squares discrimination analysis (PLS-DA) score plot (A, C) and PLS-DA permutation testing (B, D) of rumen metabolites in control and different inulin addition groups under (A, B) positive and (C, D) negative mode ionization. R^2X(cum) and R^2Y(cum) represent the cumulative interpretation rate to the X and Y matrices of the model, respectively; Q^2(cum) represents the predictive ability of the model. The closer these three indicators are to 1, the more stable and reliable the model is. Q^2(cum) > 0.5 indicates that the predictive ability of the model is better. Con = control group; I-1 = inulin-1 group, the inulin addition level was 100 g/d per cow; I-2 = inulin-2 group, the inulin addition level was 200 g/d per cow; I-3 = inulin-3 group, the inulin addition level was 300 g/d per cow; I-4 = inulin-4 group, the inulin addition level was 400 g/d per cow.

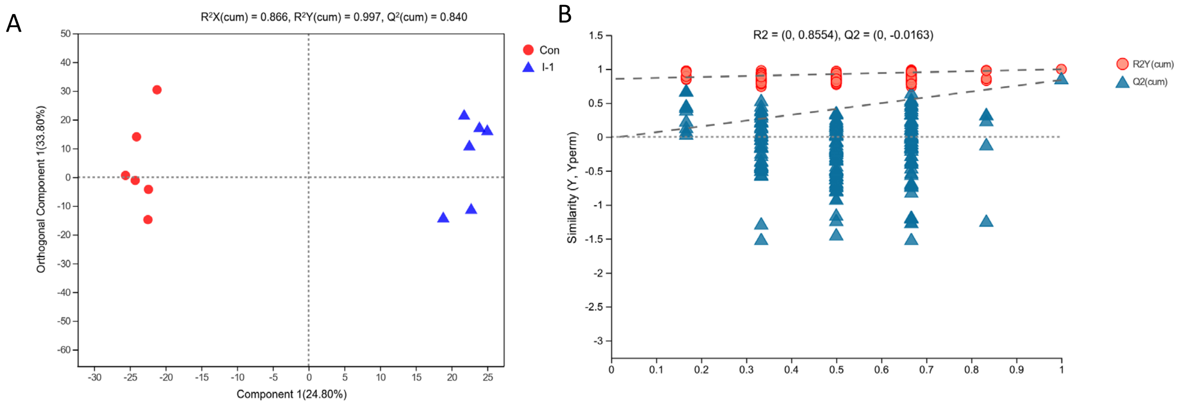

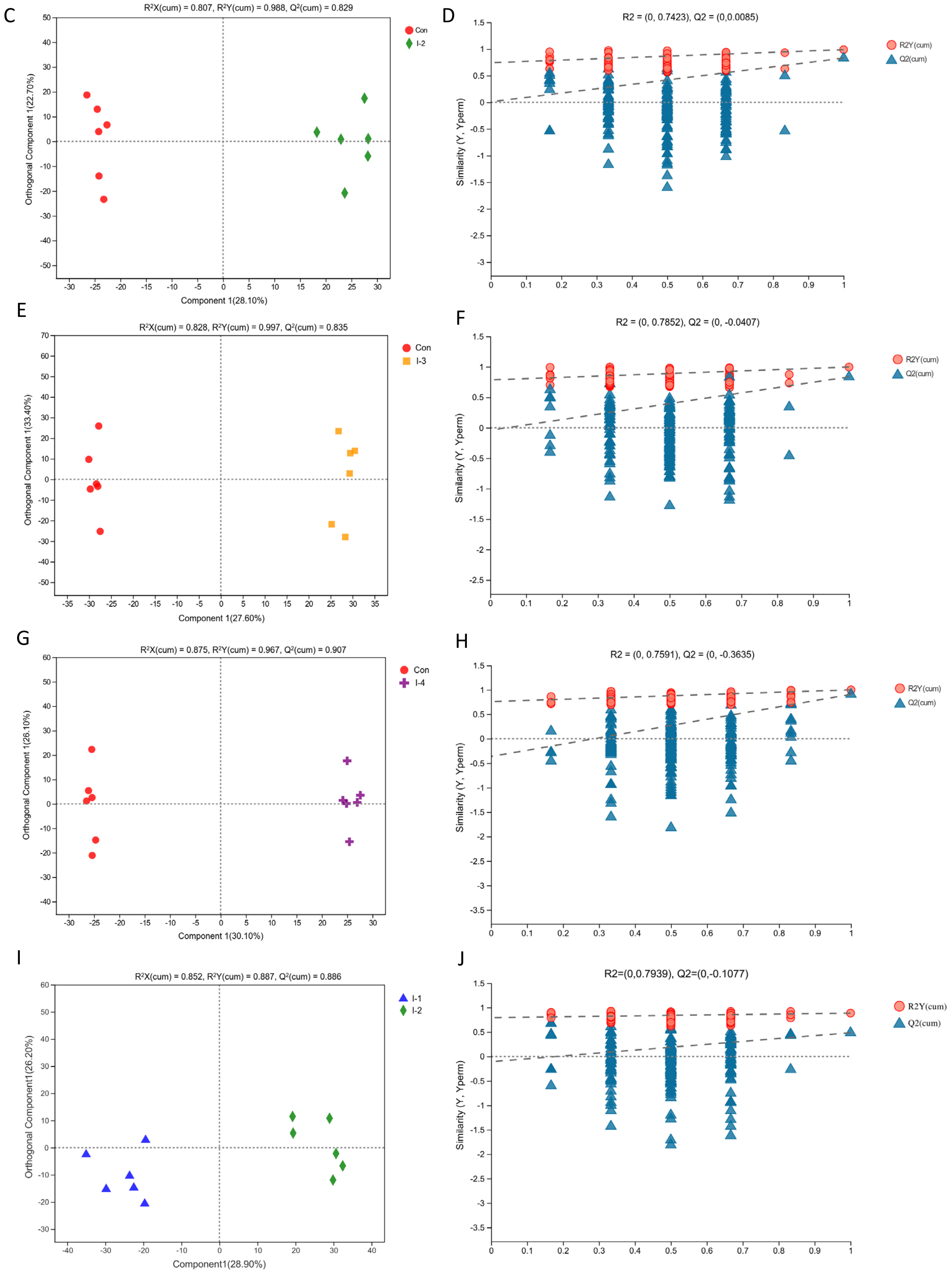
C
R²X(cum) = 0.807, R²Y(cum) = 0.988, Q²(cum) = 0.829
Con
I-2
Orthogonal Component 1(22.70%)
Component 1(28.10%)
D
R2 = (0, 0.7423), Q2 = (0,0.0085)
R2Y(cum)
Q2(cum)
Similarity (Y, Yperm)
E
R²X(cum) = 0.828, R²Y(cum) = 0.997, Q²(cum) = 0.835
Con
I-3
Orthogonal Component 1(33.40%)
Component 1(27.60%)
F
R2 = (0, 0.7852), Q2 = (0, -0.0407)
R2Y(cum)
Q2(cum)
Similarity (Y, Yperm)
G
R²X(cum) = 0.875, R²Y(cum) = 0.967, Q²(cum) = 0.907
Con
I-4
Orthogonal Component 1(26.10%)
Component 1(30.10%)
H
R2 = (0, 0.7591), Q2 = (0, -0.3635)
R2Y(cum)
Q2(cum)
Similarity (Y, Yperm)
I
R²X(cum) = 0.852, R²Y(cum) = 0.887, Q²(cum) = 0.886
I-1
I-2
Orthogonal Component1(26.20%)
Component1(28.90%)
J
R2=(0,0.7939), Q2=(0,-0.1077)
R2Y(cum)
Q2(cum)
Similarity (Y, Yperm)

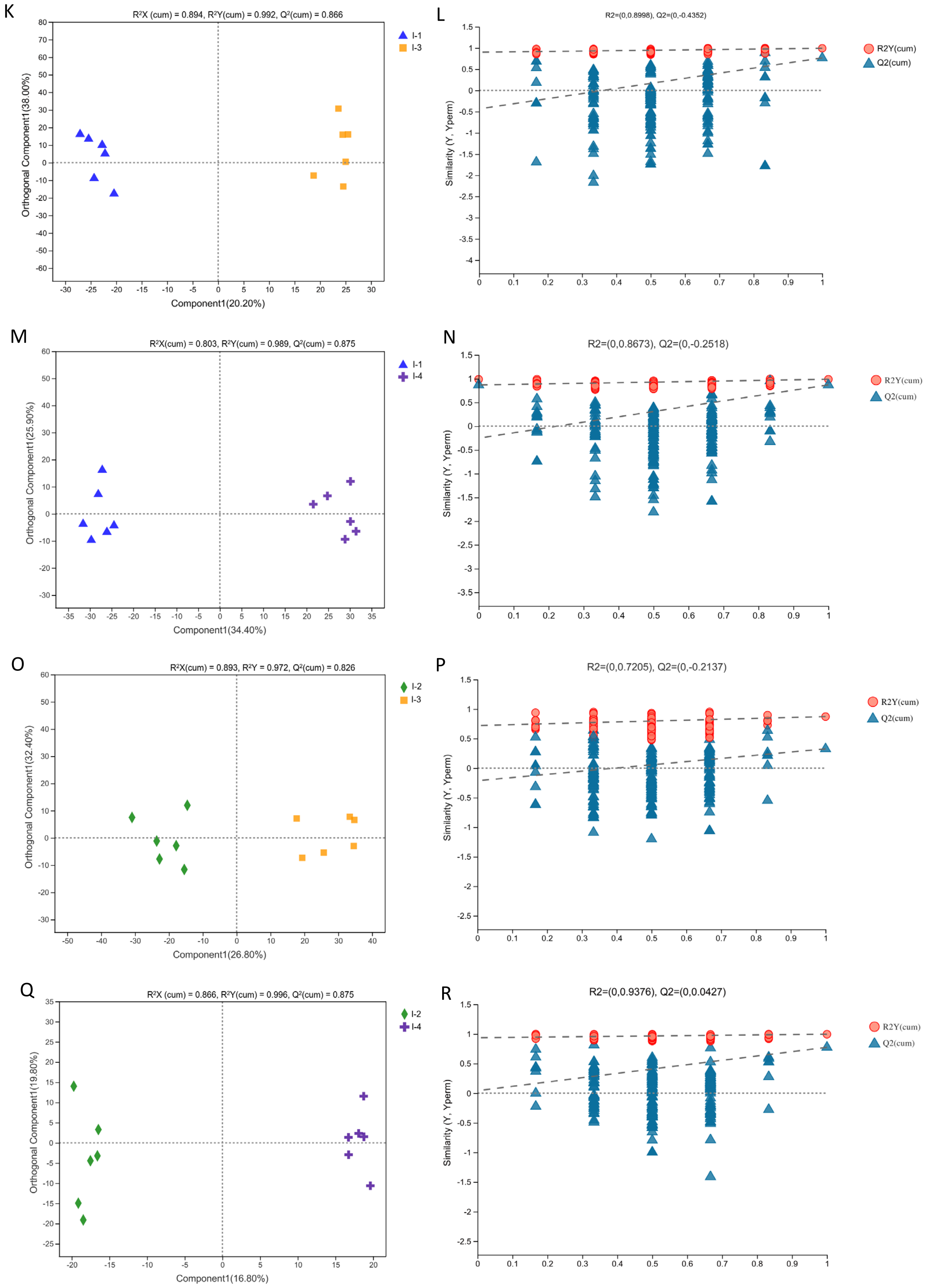
K
R²X (cum) = 0.894, R²Y(cum) = 0.992, Q²(cum) = 0.866
I-1
I-3
Orthogonal Component1(38.00%)
Component1(20.20%)
L
R2=(0,0.8998), Q2=(0,-0.4352)
R2Y(cum)
Q2(cum)
Similarity (Y, Yperm)
M
R²X(cum) = 0.803, R²Y(cum) = 0.989, Q²(cum) = 0.875
I-1
I-4
Orthogonal Component1(25.90%)
Component1(34.40%)
N
R2=(0,0.8673), Q2=(0,-0.2518)
R2Y(cum)
Q2(cum)
Similarity (Y, Yperm)
O
R²X(cum) = 0.893, R²Y = 0.972, Q²(cum) = 0.826
I-2
I-3
Orthogonal Component1(32.40%)
Component1(26.80%)
P
R2=(0,0.7205), Q2=(0,-0.2137)
R2Y(cum)
Q2(cum)
Similarity (Y, Yperm)
Q
R²X (cum) = 0.866, R²Y(cum) = 0.996, Q²(cum) = 0.875
I-2
I-4
Orthogonal Component1(19.80%)
Component1(16.80%)
R
R2=(0,0.9376), Q2=(0,0.0427)
R2Y(cum)
Q2(cum)
Similarity (Y, Yperm)

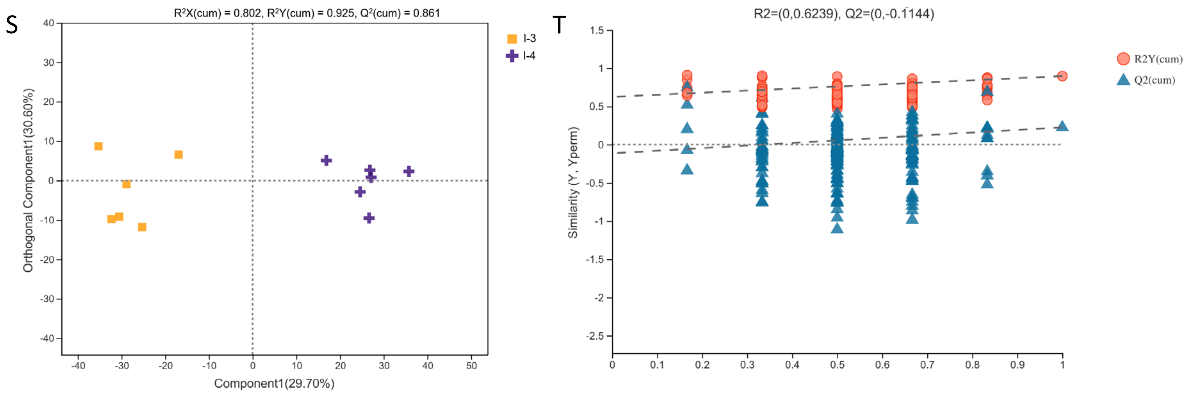

Fig. S5 Orthogonal partial least squares discrimination analysis (OPLS-DA) score plot (A, C, E, G, I, K, M, O, Q, S) and OPLS-DA permutation testing (B, D, F, H, J, L, N, P, R, T) of rumen metabolites in control and different inulin addition groups under positive ion mode. R^2X(cum) and R^2Y(cum) represent the cumulative interpretation rate to the X and Y matrices of the model, respectively; Q^2(cum) represents the predictive ability of the model. The closer these three indicators are to 1, the more stable and reliable the model is. Q^2(cum) > 0.5 indicates that the predictive ability of the model is better. Con = control group; I-1 = inulin-1 group, the inulin addition level was 100 g/d per cow; I-2 = inulin-2 group, the inulin addition level was 200 g/d per cow; I-3 = inulin-3 group, the inulin addition level was 300 g/d per cow; I-4 = inulin-4 group, the inulin addition level was 400 g/d per cow.

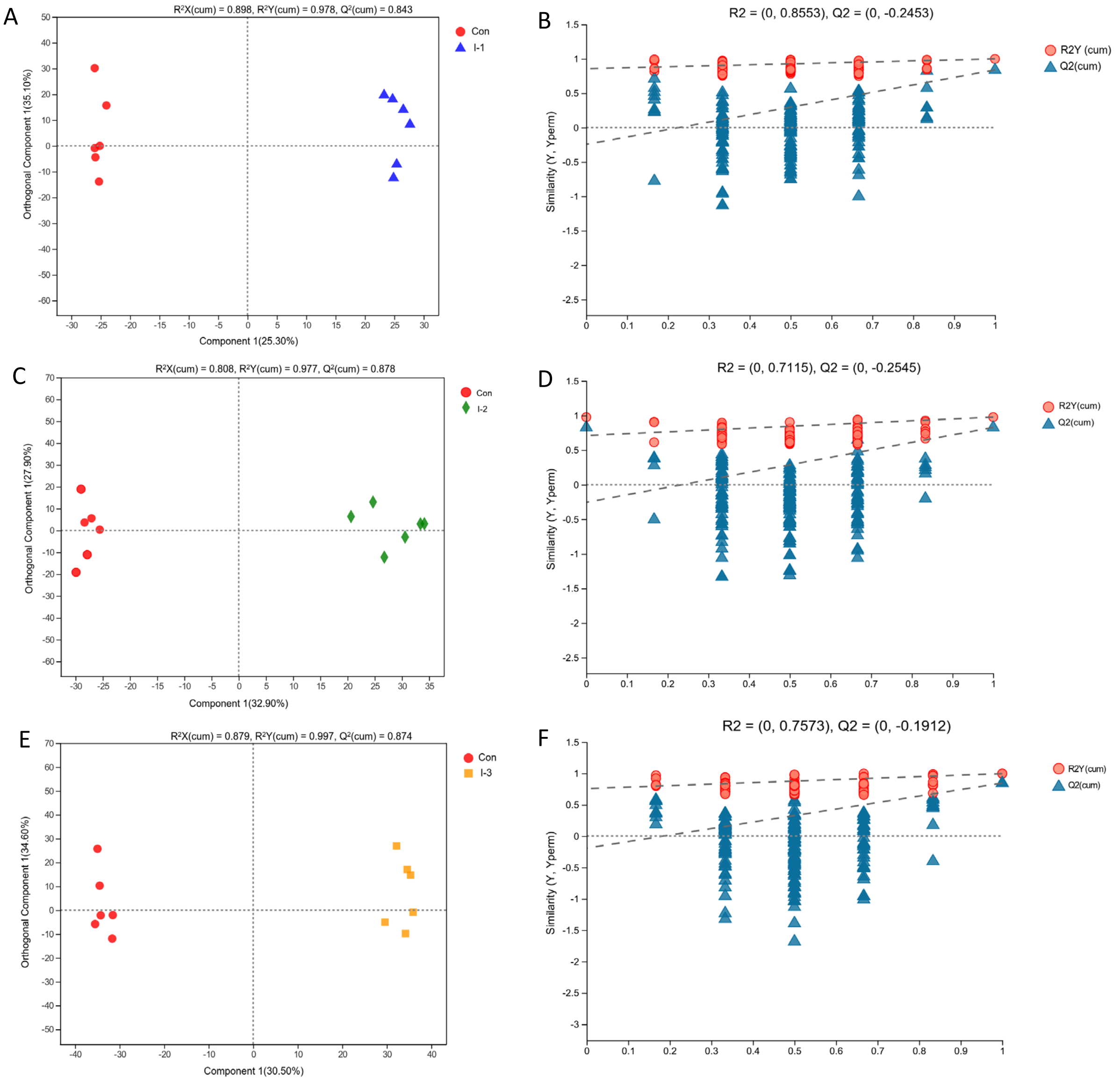

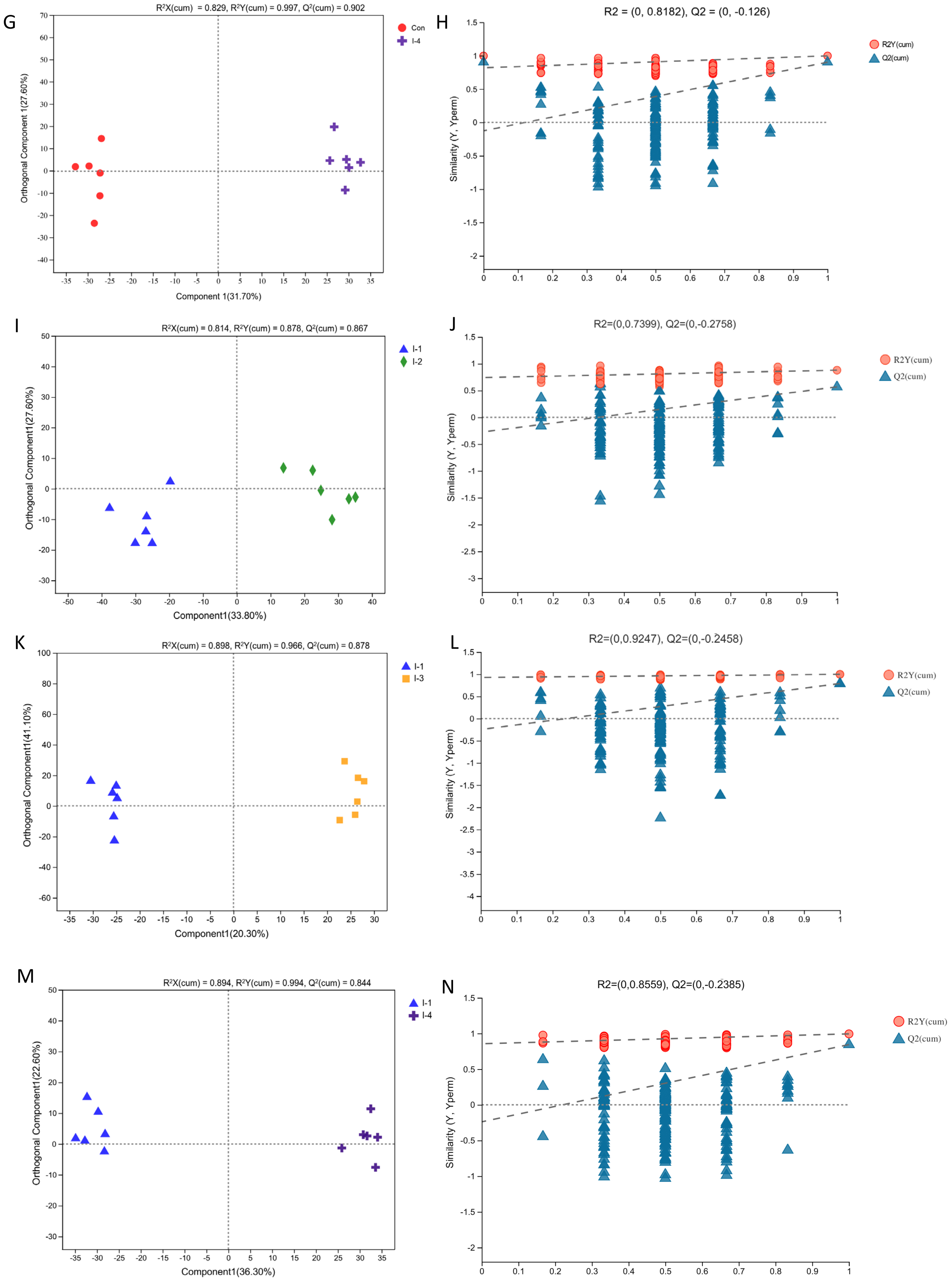

G
R²X(cum) = 0.829, R²Y(cum) = 0.997, Q²(cum) = 0.902
Con
I-4
Orthogonal Component 1(27.60%)
Component 1(31.70%)
H
R2 = (0, 0.8182), Q2 = (0, -0.126)
R2Y(cum)
Q2(cum)
Similarity (Y, Yperm)
I
R²X(cum) = 0.814, R²Y(cum) = 0.878, Q²(cum) = 0.867
I-1
I-2
Orthogonal Component1(27.60%)
Component1(33.80%)
J
R2=(0,0.7399), Q2=(0,-0.2758)
R2Y(cum)
Q2(cum)
Similarity (Y, Yperm)
K
R²X(cum) = 0.898, R²Y(cum) = 0.966, Q²(cum) = 0.878
I-1
I-3
Orthogonal Component1(41.10%)
Component1(20.30%)
L
R2=(0,0.9247), Q2=(0,-0.2458)
R2Y(cum)
Q2(cum)
Similarity (Y, Yperm)
M
R²X(cum) = 0.894, R²Y(cum) = 0.994, Q²(cum) = 0.844
I-1
I-4
Orthogonal Component1(22.60%)
Component1(36.30%)
N
R2=(0,0.8559), Q2=(0,-0.2385)
R2Y(cum)
Q2(cum)
Similarity (Y, Yperm)

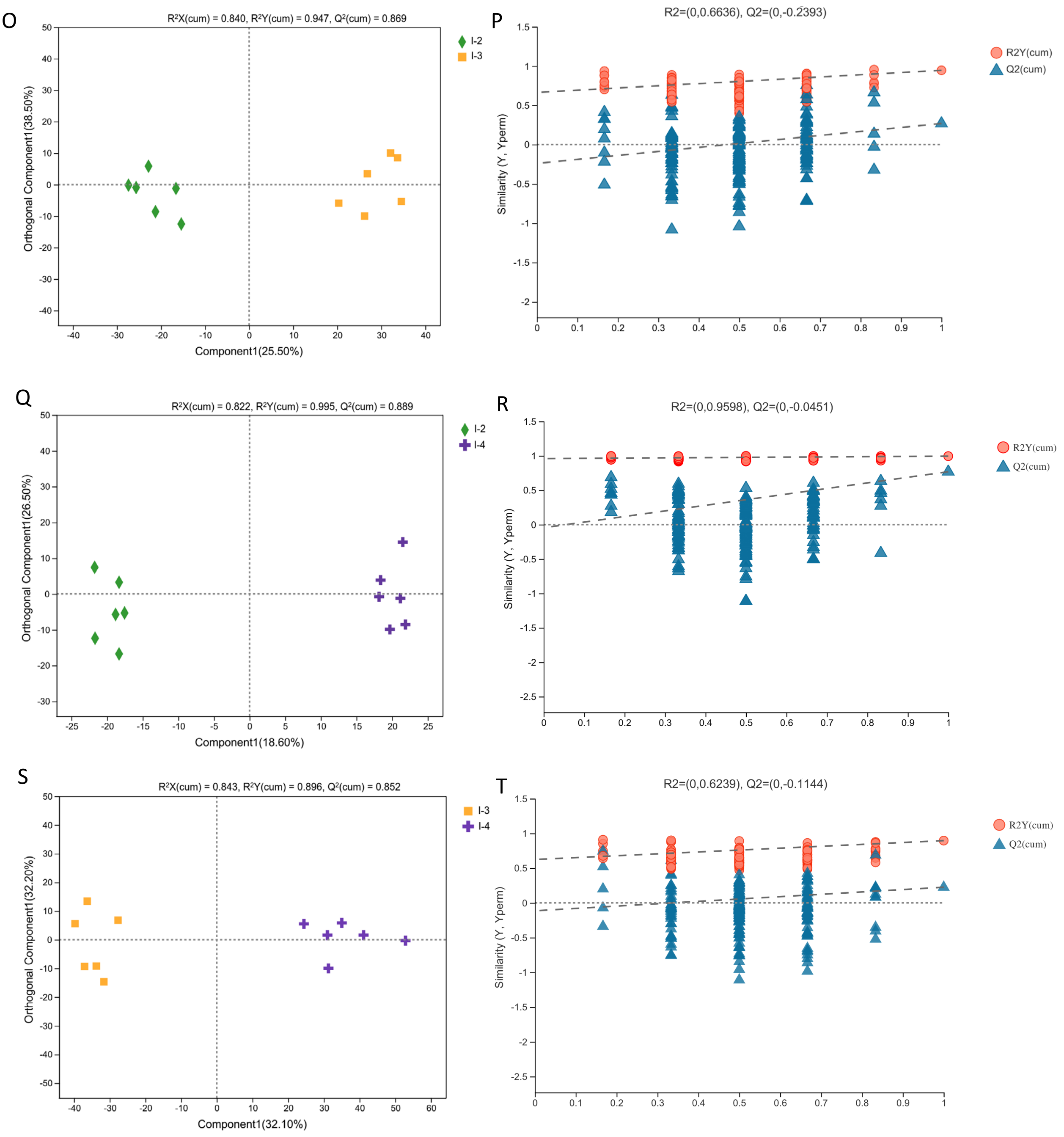

Fig. S6 Orthogonal partial least squares discrimination analysis (OPLS-DA) score plot (A, C, E, G, I, K, M, O, Q, S) and OPLS-DA response permutation testing (B, D, F, H, J, L, N, P, R, T) of rumen metabolites among control and different inulin addition groups under negative ion mode. R^2X(cum) and R^2Y(cum) represent the cumulative interpretation rate to the X and Y matrices of the model, respectively; Q^2(cum) represents the predictive ability of the model. The closer these three indicators are to 1, the more stable and reliable the model is. Q^2(cum) > 0.5 indicates that the predictive ability of the model is better. Con = control group; I-1 = inulin-1 group, the inulin addition level was 100 g/d per cow; I-2 = inulin-2 group, the inulin addition level was 200 g/d per cow; I-3 = inulin-3 group, the inulin addition level was 300 g/d per cow; I-4 = inulin-4 group, the inulin addition level was 400 g/d per cow.

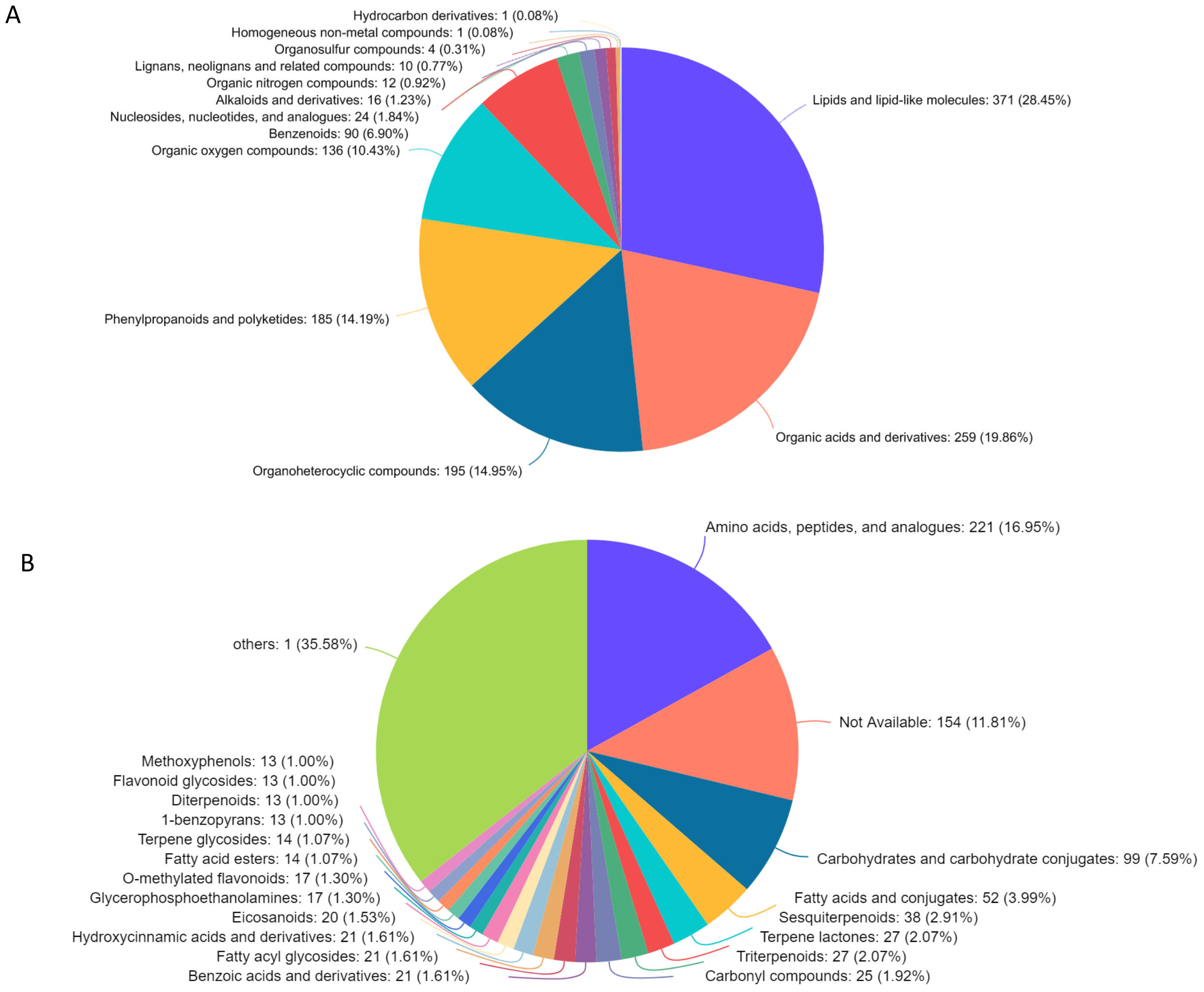

Fig. S7 Classification of metabolites based on Human metabolome database (HMDB) at (A) superclass and (B) subclass level.
